# Supplementary material for: Behavioral and brain anatomical analysis of Foxg1 heterozygous mice
Source: PLoS One. 2022 Oct 12;17(10):e0266861. doi: 10.1371/journal.pone.0266861 (PMC9555627; doi:10.1371/journal.pone.0266861)
Supplement: S1 Raw images — (PDF) [file pone.0266861.s001.pdf]

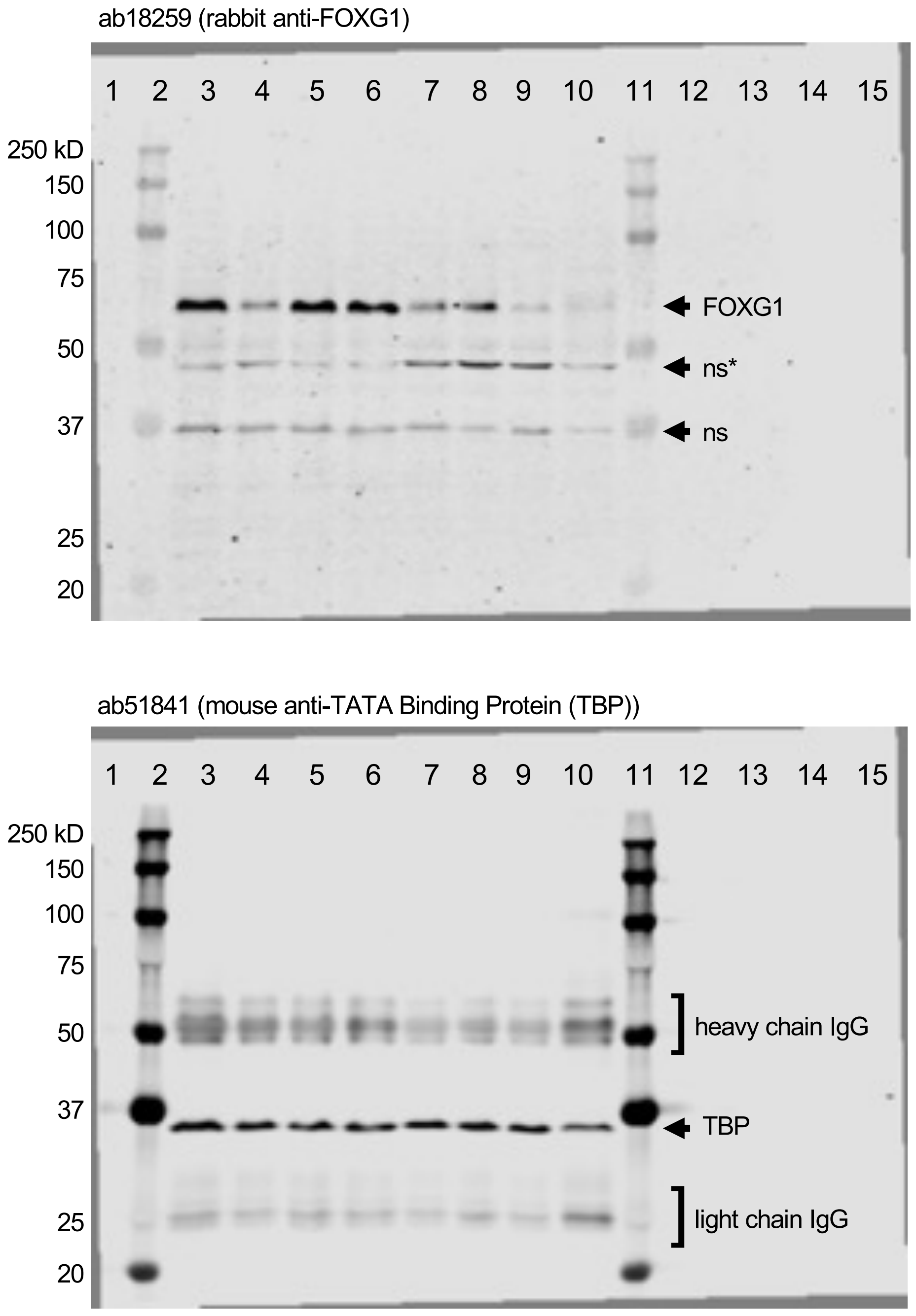

Figure S1. Original western blot scans **from Figure 1b**. **Lanes 1, 12-15:** blank; **Lanes 2, 11:** molecular weight marker; **Lanes 3-6:** WT e16.5 embryonic mouse brain lysates; **Lanes 7-10:** Foxg1-MUT e16.5 embryonic mouse brain lysates (littermates to WT); **ns:** nonspecific band; **ns*:** The ~48 kD band in the FOXG1 blot has been reported as nonspecific reactivity to ab18259 (Verginelli *et al.*, 2013) and showed a similar shift in intensity following shRNA knockdown of FOXG1 in cultured brain tumor initiating cells.
