# Supplementary material for: Behavioral and brain anatomical analysis of Foxg1 heterozygous mice
Source: PLoS One. 2022 Oct 12;17(10):e0266861. doi: 10.1371/journal.pone.0266861 (PMC9555627; doi:10.1371/journal.pone.0266861)
Supplement: S1 File — (PDF) [file pone.0266861.s003.pdf]

Supplement 2: S2_Interaction_Statistical_Output. Bold indicates p<0.05

**Weight**	**Genotype**		**Sex**		**Genotype*Sex**	
	F [1,26]	p-value	F [1,26]	p-value	F [1,26]	p-value
BODY WEIGHT (g)	1.785	0.193	105.671	**0.000**	1.017	0.322
BRAIN WEIGHT (mg)	22.485	**0.000**	6.183	**0.019**	0.512	0.480
**Rotarod**	**Genotype**		**Sex**		**Genotype*Sex**	
	F [1,26]	p-value	F [1,26]	p-value	F [1,26]	p-value
Day 1 Average	0.000	0.993	1.833	0.187	0.001	0.971
Day 2 Average	0.005	0.942	0.963	0.336	0.640	0.431
Day 3 Average	0.260	0.614	9.373	**0.005**	0.943	0.340
**Open Field Analysis**	**Genotype**		**Sex**		**Genotype*Sex**	
	F [1,53]	p-value	F [1,53]	p-value	F [1,53]	p-value
Total Ambulatory Distance	7.874	0.007	2.073	0.156	0.837	0.365
% Distance Center	53.135	0.000	0.077	0.782	0.977	0.327
% Time Center	6.876	0.011	0.009	0.926	0.155	0.696
Total Vertical Counts	6.864	0.011	6.554	0.013	3.043	0.087
**Elevated Zero Maze**	**Genotype**		**Sex**		**Genotype*Sex**	
	F [1,21]	p-value	F [1,21]	p-value	F [1,21]	p-value
Distance (Total)	0.101	0.754	0.012	0.914	0.410	0.529
% Distance Open	6.747	0.017	0.072	0.792	1.296	0.268
% Time Open	0.663	0.425	0.003	0.956	0.134	0.718
**Gait**	**Genotype**		**Sex**		**Genotype*Sex**	
	F [1,52]	p-value	F [1,52]	p-value	F [1,52]	p-value
Forepaw Stride (ms)	2.762	0.103	1.260	0.267	0.501	0.482
Forepaw % Swing	7.036	**0.011**	0.735	0.395	0.006	0.938
Forepaw Stride Length (mm)	1.922	0.172	2.162	0.147	1.034	0.314
Forepaw Print Area	6.512	**0.014**	5.615	**0.022**	0.004	0.949
Hindpaw Stride (ms)	0.025	0.876	0.224	0.638	0.727	0.398
Hindpaw % Swing	11.629	**0.001**	2.130	0.150	1.064	0.307
Hindpaw Stride Length (mm)	5.574	**0.022**	0.305	0.583	1.003	0.321
Hindpaw Print Area	4.194	**0.046**	0.012	0.914	0.007	0.932
Front Track Width (mm)	0.044	0.835	1.135	0.292	0.166	0.685
Rear Track Width (mm)	4.323	**0.043**	4.519	**0.038**	0.946	0.335
Overall Avg Run Speed (mm/s)	1.184	0.282	0.350	0.556	0.182	0.672
Forepaw Homolateral Coupling	3.518	0.066	1.502	0.226	0.272	0.604
Forepaw Homologous Coupling	0.468	0.497	2.292	0.136	0.147	0.703
Forepaw Diagonal Coupling	1.561	0.217	0.597	0.443	0.040	0.842
Hindpaw Homolateral Coupling	6.803	**0.012**	0.216	0.644	0.027	0.870
Hindpaw Homologous Coupling	5.380	**0.024**	0.217	0.644	0.340	0.562
Hindpaw Diagonal Coupling	0.515	0.476	0.052	0.821	1.132	0.292
Forepaw Gait Angle	0.178	0.675	0.584	0.448	2.365	0.130
Hindpaw Gait Angle	0.370	0.546	0.283	0.597	0.315	0.577
Body Rotation (deg)	10.580	**0.002**	0.033	0.857	0.697	0.408
Longitudinal Position (mm)	0.447	0.507	0.615	0.436	0.691	0.410
Lateral Position (mm)	0.122	0.728	0.147	0.703	0.043	0.836
**Fear Conditioning**	**Genotype**		**Sex**		**Genotype*Sex**	
	F [1,26]	p-value	F [1,26]	p-value	F [1,26]	p-value
Pre-stim	12.801	**0.001**	1.561	0.223	0.001	0.979
Context-Prestim	24.417	**0.000**	0.195	0.663	0.037	0.848
Cue-Prestim	25.389	**0.000**	1.316	0.262	0.219	0.644
**Marble Burying**	**Genotype**		**Sex**		**Genotype*Sex**	
	F [1,26]	p-value	F [1,26]	p-value	F [1,26]	p-value
Marble (% burried)	4.501	**0.044**	0.389	0.538	0.148	0.703
**Tube test**	**Genotype**		**Sex**		**Genotype*Sex**	
	F [1,26]	p-value	F [1,26]	p-value	F [1,26]	p-value
Avg wins	6.834	**0.015**	2.162	0.153	2.162	0.153
**Nest Building**	**Genotype**		**Sex**		**Genotype*Sex**	
	F [1,26]	p-value	F [1,26]	p-value	F [1,26]	p-value
Nest Score	6.189	**0.020**	0.007	0.933	0.548	0.466
% shredded	3.764	0.063	0.126	0.725	1.392	0.249
**3 Chamber**	**Genotype**		**Sex**		**Genotype*Sex**	
	F [1,23]	p-value	F [1,23]	p-value	F [1,23]	p-value
Social Index Cup	3.903	0.060	0.808	0.378	0.013	0.910
Social Index Chamber	4.637	0.042	0.238	0.630	0.135	0.716
Novelty Index Cup	3.597	0.071	0.013	0.910	1.032	0.320
Novelty Index Chamber	5.234	**0.032**	0.654	0.427	0.739	0.399
