# Supplementary material for: Behavioral and brain anatomical analysis of Foxg1 heterozygous mice
Source: PLoS One. 2022 Oct 12;17(10):e0266861. doi: 10.1371/journal.pone.0266861 (PMC9555627; doi:10.1371/journal.pone.0266861)
Supplement: S2 File — (PDF) [file pone.0266861.s004.pdf]

Supplement 3: S3_Two_Way_Statistical_Output. Bold indicates p<0.05. η2 values only presented for p<0.05.

**Weight**	**Genotype**			**Sex**		
	F [1,27]	p-value	η^2	F [1,27]	p-value	η^2
BODY WEIGHT (g)	1.977	0.171		105.604	**0.000**	**0.796**
BRAIN WEIGHT (mg)	22.339	**0.000**	**0.365**	6.282	**0.018**	**0.155**
**Rotarod**	**Genotype**			**Sex**		
	F [1,27]	p-value	η^2	F [1,27]	p-value	η^2
Day 1 Average	0.000	0.991		1.903	0.179	
Day 2 Average	0.000	0.984		0.976	0.332	
Day 3 Average	0.333	0.569		9.393	**0.005**	**0.258**
**Open Field Analysis**	**Genotype**			**Sex**		
	F [1,54]	p-value	η^2	F [1,54]	p-value	η^2
Total Ambulatory Distance	7.885	**0.007**	**0.127**	2.329	0.133	
% Distance Center	53.193	**0.000**	**0.496**	0.133	0.717	
% Time Center	6.991	**0.011**	**0.115**	0.017	0.898	
Total Vertical Counts	6.592	**0.013**	**0.109**	7.130	0.010	**0.117**
**Elevated Zero Maze**	**Genotype**			**Sex**		
	F [1,22]	p-value	η^2	F [1,22]	p-value	η^2
Distance (Total)	0.326	0.574		0.069	0.796	
% Distance Open	9.838	**0.005**	**0.309**	0.000	0.988	
% Time Open	1.027	0.322		0.001	0.979	
**Gait**	**Genotype**			**Sex**		
	F [1,53]	p-value	η^2	F [1,53]	p-value	η^2
Forepaw Stride (ms)	2.840	0.098		1.174	0.283	
Forepaw % Swing	7.183	**0.010**	**0.119**	0.743	0.393	
Forepaw Stride Length (mm)	1.862	0.178		1.978	0.165	
Forepaw Print Area	6.647	**0.013**	**0.111**	5.726	**0.020**	**0.098**
Hindpaw Stride (ms)	0.031	0.861		0.283	0.597	
Hindpaw % Swing	11.770	**0.001**	**0.182**	1.943	0.169	
Hindpaw Stride Length (mm)	5.476	**0.023**	**0.094**	0.384	0.538	
Hindpaw Print Area	4.269	**0.044**	**0.075**	0.013	0.909	
Front Track Width (mm)	0.041	0.840		1.217	0.275	
Rear Track Width (mm)	4.417	**0.040**	**0.077**	4.275	**0.044**	**0.075**
Overall Avg Run Speed (mm/s)	1.223	0.274		0.324	0.571	
Forepaw Homolateral Coupling	3.611	0.063		1.446	0.235	
Forepaw Homologous Coupling	0.487	0.488		2.418	0.126	
Forepaw Diagonal Coupling	1.601	0.211		0.590	0.446	
Hindpaw Homolateral Coupling	6.952	**0.011**	**0.116**	0.211	0.648	
Hindpaw Homologous Coupling	5.392	**0.024**	**0.092**	0.258	0.614	
Hindpaw Diagonal Coupling	0.547	0.463		0.088	0.768	
Forepaw Gait Angle	0.202	0.655		0.431	0.514	
Hindpaw Gait Angle	0.390	0.535		0.249	0.620	
Body Rotation (deg)	10.529	**0.002**	**0.166**	0.056	0.814	
Longitudinal Position (mm)	0.426	0.517		0.538	0.466	
Lateral Position (mm)	0.127	0.722		0.140	0.710	
**Fear Conditioning**	**Genotype**			**Sex**		
	F [1,27]	p-value	η^2	F [1,27]	p-value	η^2
Pre-stim	13.365	**0.001**	**0.331**	1.621	0.214	
Context-Prestim	25.566	**0.000**	**0.486**	0.202	0.657	
Cue-Prestim	26.588	**0.000**	**0.496**	1.355	0.255	
**Marble Burying**	**Genotype**			**Sex**		
	F [1,27]	p-value	η^2	F [1,27]	p-value	η^2
Marble (% burried)	4.782	**0.038**	**0.150**	0.402	0.531	
**Tube test**	**Genotype**			**Sex**		
	F [1,27]	p-value	η^2	F [1,27]	p-value	η^2
Avg wins	7.105	**0.013**	**0.208**	2.150	0.154	
**Nest Building**	**Genotype**			**Sex**		
	F [1,27]	p-value	η^2	F [1,27]	p-value	η^2
Nest Score	6.5757	**0.0162**	**0.1958**	0.0073	0.9324	
% shredded	4.0349	0.0547		0.1244	0.7270	
**3 Chamber**	**Genotype**			**Sex**		
	F [1,24]	p-value	η^2	F [1,24]	p-value	η^2
Social Index Cup	4.071	0.055		0.836	0.370	
Social Index Chamber	4.684	**0.041**	**0.163**	0.182	0.674	
Novelty Index Cup	3.201	0.086		0.011	0.916	
Novelty Index Chamber	4.905	**0.037**	**0.170**	1.046	0.317	
