# Supplementary material for: Behavioral and brain anatomical analysis of Foxg1 heterozygous mice
Source: PLoS One. 2022 Oct 12;17(10):e0266861. doi: 10.1371/journal.pone.0266861 (PMC9555627; doi:10.1371/journal.pone.0266861)
Supplement: S3 File — (PDF) [file pone.0266861.s005.pdf]

Supplement S4_SPSS_Statistical Output

The full output of the statistical analysis performed in SPSS follows, with title sheets indicating the specific behavioral measure output on subsequent pages.

Weights (Body and Brain)

```
UNIANOVA BODYWEIGHTg BY GENOTYPE SEX
/METHOD=SSTYPE(3)
/INTERCEPT=INCLUDE
/CRITERIA=ALPHA(0.05)
/DESIGN=GENOTYPE SEX GENOTYPE*SEX.
```

Univariate Analysis of Variance

Notes

Output Created		22-AUG-2022 19:43:...
Comments		
Input	Data	/Users/jeffneul/Jeff's Research/Neul Manuscripts/FoxG1/June_Aug 2022 stat sex and effect size/SPSS files/SPSS Data Files/Aug 2022 BrainBodyWT.sav
	Active Dataset	DataSet2
	Filter	<none>
	Weight	<none>
	Split File	<none>
	N of Rows in Working Data File	34
Missing Value Handling	Definition of Missing	User-defined missing values are treated as missing.
	Cases Used	Statistics are based on all cases with valid data for all variables in the model.
Syntax		UNIANOVA BODYWEIGHTg BY GENOTYPE SEX /METHOD=SSTYPE(3) /INTERCEPT=INCLUDE /CRITERIA=ALPHA(0.05) /DESIGN=GENOTYPE SEX GENOTYPE*SEX.
Resources	Processor Time	00:00:00.01
	Elapsed Time	00:00:00.00

Between-Subjects Factors

		N
GENOTYPE	KI	15
	WT	15
SEX	F	14
	M	16

Tests of Between-Subjects Effects

Dependent Variable: BODY WEIGHT (g)

Source	Type III Sum of Squares	df	Mean Square	F	Sig.
Corrected Model	186.783[a]	3	62.261	36.222	<.001
Intercept	14010.728	1	14010.728	8151.175	<.001
GENOTYPE	3.069	1	3.069	1.785	.193
SEX	181.634	1	181.634	105.671	<.001
GENOTYPE * SEX	1.749	1	1.749	1.017	.322
Error	44.690	26	1.719		
Total	14519.210	30			
Corrected Total	231.474	29			

a. R Squared = .807 (Adjusted R Squared = .785)

```
UNIANOVA BRAINWEIGHTmg BY GENOTYPE SEX
/METHOD=SSTYPE(3)
/INTERCEPT=INCLUDE
/CRITERIA=ALPHA(0.05)
/DESIGN=GENOTYPE SEX GENOTYPE*SEX.
```

Univariate Analysis of Variance

Notes

Output Created		22-AUG-2022 19:43:...
Comments		
Input	Data	/Users/jeffneul/Jeff's Research/Neul Manuscripts/FoxG1/June_Aug 2022 stat sex and effect size/SPSS files/SPSS Data Files/Aug 2022 BrainBodyWT.sav
	Active Dataset	DataSet2
	Filter	<none>
	Weight	<none>
	Split File	<none>
	N of Rows in Working Data File	34
Missing Value Handling	Definition of Missing	User-defined missing values are treated as missing.
	Cases Used	Statistics are based on all cases with valid data for all variables in the model.
Syntax		UNIANOVA BRAINWEIGHTmg BY GENOTYPE SEX /METHOD=SSTYPE(3) /INTERCEPT=INCLUDE /CRITERIA=ALPHA(0.05) /DESIGN=GENOTYPE SEX GENOTYPE*SEX.
Resources	Processor Time	00:00:00.01
	Elapsed Time	00:00:00.00

Between-Subjects Factors

		N
GENOTYPE	KI	17
	WT	17
SEX	F	14
	M	20

Tests of Between-Subjects Effects

Dependent Variable: BRAIN WEIGHT (mg)

Source	Type III Sum of Squares	df	Mean Square	F	Sig.
Corrected Model	10302.897 [a]	3	3434.299	9.561	<.001
Intercept	6328113.52	1	6328113.52	17617.164	<.001
GENOTYPE	8076.507	1	8076.507	22.485	<.001
SEX	2220.950	1	2220.950	6.183	.019
GENOTYPE * SEX	183.969	1	183.969	.512	.480
Error	10776.048	30	359.202		
Total	6509480.94	34			
Corrected Total	21078.945	33			

a. R Squared = .489 (Adjusted R Squared = .438)

```
UNIANOVA BODYWEIGHTg BY GENOTYPE SEX
/METHOD=SSTYPE(3)
/INTERCEPT=INCLUDE
/EMMEANS=TABLES(GENOTYPE)
/EMMEANS=TABLES(SEX)
/CRITERIA=ALPHA(0.05)
/DESIGN=GENOTYPE SEX.
```

Univariate Analysis of Variance

Notes

Output Created		22-AUG-2022 19:44:...
Comments		
Input	Data	/Users/jeffneul/Jeff's Research/Neul Manuscripts/FoxG1/June _Aug 2022 stat sex and effect size/SPSS files/SPSS Data Files/Aug 2022 BrainBodyWT.sav
	Active Dataset	DataSet2
	Filter	<none>
	Weight	<none>
	Split File	<none>
	N of Rows in Working Data File	34
Missing Value Handling	Definition of Missing	User-defined missing values are treated as missing.
	Cases Used	Statistics are based on all cases with valid data for all variables in the model.
Syntax		UNIANOVA BODYWEIGHTg BY GENOTYPE SEX /METHOD=SSTYPE(3) /INTERCEPT=INCLUDE /EMMEANS=TABLES (GENOTYPE) /EMMEANS=TABLES (SEX) /CRITERIA=ALPHA (0.05) /DESIGN=GENOTYPE SEX.
Resources	Processor Time	00:00:00.01
	Elapsed Time	00:00:00.00

Between-Subjects Factors

		N
GENOTYPE	KI	15
	WT	15
SEX	F	14
	M	16

Tests of Between-Subjects Effects

Dependent Variable: BODY WEIGHT (g)

Source	Type III Sum of Squares	df	Mean Square	F	Sig.
Corrected Model	185.035[a]	2	92.517	53.790	<.001
Intercept	14010.728	1	14010.728	8145.956	<.001
GENOTYPE	3.400	1	3.400	1.977	.171
SEX	181.634	1	181.634	105.604	<.001
Error	46.439	27	1.720		
Total	14519.210	30			
Corrected Total	231.474	29			

a. R Squared = .799 (Adjusted R Squared = .785)

Estimated Marginal Means

1. GENOTYPE

Dependent Variable: BODY WEIGHT (g)

GENOTYPE	Mean	Std. Error	95% Confidence Interval	
			Lower Bound	Upper Bound
KI	21.996	.339	21.300	22.691
WT	21.322	.339	20.627	22.018

2. SEX

Dependent Variable: BODY WEIGHT (g)

SEX	Mean	Std. Error	95% Confidence Interval	
			Lower Bound	Upper Bound
F	19.193	.351	18.474	19.912
M	24.125	.328	23.452	24.798

```
UNIANOVA BRAINWEIGHTmg BY GENOTYPE SEX
/METHOD=SSTYPE(3)
/INTERCEPT=INCLUDE
/EMMEANS=TABLES(GENOTYPE)
/EMMEANS=TABLES(SEX)
/CRITERIA=ALPHA(0.05)
/DESIGN=GENOTYPE SEX.
```

Univariate Analysis of Variance

Notes

Output Created		22-AUG-2022 19:49:...
Comments		
Input	Data	/Users/jeffneul/Jeff's Research/Neul Manuscripts/FoxG1/June _Aug 2022 stat sex and effect size/SPSS files/SPSS Data Files/Aug 2022 BrainBodyWT.sav
	Active Dataset	DataSet2
	Filter	<none>
	Weight	<none>
	Split File	<none>
	N of Rows in Working Data File	34
Missing Value Handling	Definition of Missing	User-defined missing values are treated as missing.
	Cases Used	Statistics are based on all cases with valid data for all variables in the model.
Syntax		UNIANOVA BRAINWEIGHTmg BY GENOTYPE SEX /METHOD=SSTYPE(3) /INTERCEPT=INCLUDE /EMMEANS=TABLES (GENOTYPE) /EMMEANS=TABLES (SEX) /CRITERIA=ALPHA (0.05) /DESIGN=GENOTYPE SEX.
Resources	Processor Time	00:00:00.01
	Elapsed Time	00:00:00.00

Between-Subjects Factors

		N
GENOTYPE	KI	17
	WT	17
SEX	F	14
	M	20

Tests of Between-Subjects Effects

Dependent Variable: BRAIN WEIGHT (mg)

Source	Type III Sum of Squares	df	Mean Square	F	Sig.
Corrected Model	10118.928 [a]	2	5059.464	14.311	<.001
Intercept	6328113.52	1	6328113.52	17898.833	<.001
GENOTYPE	7897.978	1	7897.978	22.339	<.001
SEX	2220.950	1	2220.950	6.282	.018
Error	10960.017	31	353.549		
Total	6509480.94	34			
Corrected Total	21078.945	33			

a. R Squared = .480 (Adjusted R Squared = .447)

Estimated Marginal Means

1. GENOTYPE

Dependent Variable: BRAIN WEIGHT (mg)

GENOTYPE	Mean	Std. Error	95% Confidence Interval	
			Lower Bound	Upper Bound
KI	423.055	4.597	413.680	432.430
WT	453.537	4.597	444.162	462.913

2. SEX

Dependent Variable: BRAIN WEIGHT (mg)

SEX	Mean	Std. Error	95% Confidence Interval	
			Lower Bound	Upper Bound
F	446.507	5.025	436.258	456.756
M	430.085	4.204	421.510	438.660

Rotarod

```
UNIANOVA Day1Average BY SEX GENOTYPE
/METHOD=SSTYPE(3)
/INTERCEPT=INCLUDE
/CRITERIA=ALPHA(.05)
/DESIGN=SEX GENOTYPE GENOTYPE*SEX.
```

Univariate Analysis of Variance

Notes

Output Created		19-AUG-2022 11:51:...
Comments		
Input	Data	/Users/jeffneul/Jeff's Research/Neul Manuscripts/FoxG1/June 2022 stat sex and effect size/SPSS files/SPSS Data Files/June 2022 RR sex EF data.sav
	Active Dataset	DataSet1
	Filter	<none>
	Weight	<none>
	Split File	<none>
	N of Rows in Working Data File	30
Missing Value Handling	Definition of Missing	User-defined missing values are treated as missing.
	Cases Used	Statistics are based on all cases with valid data for all variables in the model.
Syntax		UNIANOVA Day1Average BY SEX GENOTYPE /METHOD=SSTYPE(3) /INTERCEPT=INCLUDE /CRITERIA=ALPHA(.05) /DESIGN=SEX GENOTYPE GENOTYPE*SEX.
Resources	Processor Time	00:00:00.01
	Elapsed Time	00:00:00.00

```
[DataSet1] /Users/jeffneul/Jeff's Research/Neul Manuscripts/FoxG1/June 2022 st
at sex and effect size/SPSS files/SPSS Data Files/June 2022 RR sex EF data.sav
```

Between-Subjects Factors

		N
SEX	F	14
	M	16
GENOTYPE	KI	15
	WT	15

Tests of Between-Subjects Effects

Dependent Variable: Day 1 Average

Source	Type III Sum of Squares	df	Mean Square	F	Sig.
Corrected Model	8916.031 [a]	3	2972.010	.611	.614
Intercept	1055841.44	1	1055841.44	217.186	<.001
SEX	8908.680	1	8908.680	1.833	.187
GENOTYPE	.343	1	.343	.000	.993
SEX * GENOTYPE	6.772	1	6.772	.001	.971
Error	126398.107	26	4861.466		
Total	1208898.03	30			
Corrected Total	135314.138	29			

a. R Squared = .066 (Adjusted R Squared = -.042)

```
UNIANOVA Day2Average BY SEX GENOTYPE
/METHOD=SSTYPE(3)
/INTERCEPT=INCLUDE
/CRITERIA=ALPHA(.05)
/DESIGN=SEX GENOTYPE GENOTYPE*SEX.
```

Univariate Analysis of Variance

Notes

Output Created		19-AUG-2022 11:52:...
Comments		
Input	Data	/Users/jeffneul/Jeff's Research/Neul Manuscripts/FoxG1/June 2022 stat sex and effect size/SPSS files/SPSS Data Files/June 2022 RR sex EF data.sav
	Active Dataset	DataSet1
	Filter	<none>
	Weight	<none>
	Split File	<none>
	N of Rows in Working Data File	30
Missing Value Handling	Definition of Missing	User-defined missing values are treated as missing.
	Cases Used	Statistics are based on all cases with valid data for all variables in the model.
Syntax		UNIANOVA Day2Average BY SEX GENOTYPE<br>/METHOD=SSTYPE(3)<br>/INTERCEPT=INCLUDE<br>/CRITERIA=ALPHA(.05)<br>/DESIGN=SEX GENOTYPE GENOTYPE*SEX.
Resources	Processor Time	00:00:00.01
	Elapsed Time	00:00:00.00

Between-Subjects Factors

		N
SEX	F	14
	M	16
GENOTYPE	KI	15
	WT	15

Tests of Between-Subjects Effects

Dependent Variable: Day 2 Average

Source	Type III Sum of Squares	df	Mean Square	F	Sig.
Corrected Model	4820.426 [a]	3	1606.809	.534	.663
Intercept	1458875.03	1	1458875.03	485.153	<.001
SEX	2894.938	1	2894.938	.963	.336
GENOTYPE	16.140	1	16.140	.005	.942
SEX * GENOTYPE	1924.288	1	1924.288	.640	.431
Error	78183.115	26	3007.043		
Total	1539700.67	30			
Corrected Total	83003.541	29			

a. R Squared = .058 (Adjusted R Squared = -.051)

```
UNIANOVA Day3Average BY SEX GENOTYPE
/METHOD=SSTYPE(3)
/INTERCEPT=INCLUDE
/CRITERIA=ALPHA(.05)
/DESIGN=SEX GENOTYPE GENOTYPE*SEX.
```

Univariate Analysis of Variance

Notes

Output Created		19-AUG-2022 11:52:...
Comments		
Input	Data	/Users/jeffneul/Jeff's Research/Neul Manuscripts/FoxG1/June 2022 stat sex and effect size/SPSS files/SPSS Data Files/June 2022 RR sex EF data.sav
	Active Dataset	DataSet1
	Filter	<none>
	Weight	<none>
	Split File	<none>
	N of Rows in Working Data File	30
Missing Value Handling	Definition of Missing	User-defined missing values are treated as missing.
	Cases Used	Statistics are based on all cases with valid data for all variables in the model.
Syntax		UNIANOVA Day3Average BY SEX GENOTYPE<br>/METHOD=SSTYPE(3)<br>/INTERCEPT=INCLUDE<br>/CRITERIA=ALPHA(.05)<br>/DESIGN=SEX GENOTYPE GENOTYPE*SEX.
Resources	Processor Time	00:00:00.01
	Elapsed Time	00:00:00.00

Between-Subjects Factors

		N
SEX	F	14
	M	16
GENOTYPE	KI	15
	WT	15

Tests of Between-Subjects Effects

Dependent Variable: Day 3 Average

Source	Type III Sum of Squares	df	Mean Square	F	Sig.
Corrected Model	25689.412 [a]	3	8563.137	3.549	.028
Intercept	1685785.74	1	1685785.74	698.720	<.001
SEX	22613.564	1	22613.564	9.373	.005
GENOTYPE	627.815	1	627.815	.260	.614
SEX * GENOTYPE	2275.015	1	2275.015	.943	.340
Error	62729.629	26	2412.678		
Total	1755682.33	30			
Corrected Total	88419.041	29			

a. R Squared = .291 (Adjusted R Squared = .209)

```
UNIANOVA Day1Average BY SEX GENOTYPE
/METHOD=SSTYPE(3)
/INTERCEPT=INCLUDE
/EMMEANS=TABLES(SEX)
/EMMEANS=TABLES(GENOTYPE)
/PRINT ETASQ
/CRITERIA=ALPHA(.05)
/DESIGN=SEX GENOTYPE.
```

Univariate Analysis of Variance

Notes

Output Created		19-AUG-2022 11:55:...
Comments		
Input	Data	/Users/jeffneul/Jeff's Research/Neul Manuscripts/FoxG1/June 2022 stat sex and effect size/SPSS files/SPSS Data Files/June 2022 RR sex EF data.sav
	Active Dataset	DataSet1
	Filter	<none>
	Weight	<none>
	Split File	<none>
	N of Rows in Working Data File	30
Missing Value Handling	Definition of Missing	User-defined missing values are treated as missing.
	Cases Used	Statistics are based on all cases with valid data for all variables in the model.
Syntax		UNIANOVA Day1Average BY SEX GENOTYPE<br>/METHOD=SSTYPE(3)<br>/INTERCEPT=INCLUDE<br>/EMMEANS=TABLES (SEX)<br>/EMMEANS=TABLES (GENOTYPE)<br>/PRINT ETASQ<br>/CRITERIA=ALPHA(.05)<br>/DESIGN=SEX GENOTYPE.
Resources	Processor Time	00:00:00.01
	Elapsed Time	00:00:00.00

Between-Subjects Factors

		N
SEX	F	14
	M	16
GENOTYPE	KI	15
	WT	15

Tests of Between-Subjects Effects

Dependent Variable: Day 1 Average

Source	Type III Sum of Squares	df	Mean Square	F	Sig.	Partial Eta Squared
Corrected Model	8909.258 [a]	2	4454.629	.952	.399	.066
Intercept	1055841.44	1	1055841.44	225.527	<.001	.893
SEX	8908.680	1	8908.680	1.903	.179	.066
GENOTYPE	.579	1	.579	.000	.991	.000
Error	126404.880	27	4681.662			
Total	1208898.03	30				
Corrected Total	135314.138	29				

a. R Squared = .066 (Adjusted R Squared = -.003)

Estimated Marginal Means

1. SEX

Dependent Variable: Day 1 Average

SEX	Mean	Std. Error	95% Confidence Interval Lower Bound	Upper Bound
F	170.750	18.287	133.229	208.271
M	205.292	17.106	170.194	240.390

2. GENOTYPE

Dependent Variable: Day 1 Average

GENOTYPE	Mean	Std. Error	95% Confidence Interval Lower Bound	Upper Bound
KI	188.160	17.686	151.870	224.449
WT	187.882	17.686	151.593	224.171

```
UNIANOVA Day2Average BY SEX GENOTYPE
/METHOD=SSTYPE(3)
/INTERCEPT=INCLUDE
/EMMEANS=TABLES(SEX)
/EMMEANS=TABLES(GENOTYPE)
/PRINT ETASQ
/CRITERIA=ALPHA(.05)
/DESIGN=SEX GENOTYPE.
```

Univariate Analysis of Variance

Notes

Output Created		19-AUG-2022 11:55:...
Comments		
Input	Data	/Users/jeffneul/Jeff's Research/Neul Manuscripts/FoxG1/June 2022 stat sex and effect size/SPSS files/SPSS Data Files/June 2022 RR sex EF data.sav
	Active Dataset	DataSet1
	Filter	<none>
	Weight	<none>
	Split File	<none>
	N of Rows in Working Data File	30
Missing Value Handling	Definition of Missing	User-defined missing values are treated as missing.
	Cases Used	Statistics are based on all cases with valid data for all variables in the model.
Syntax		UNIANOVA Day2Average BY SEX GENOTYPE<br>/METHOD=SSTYPE(3)<br>/INTERCEPT=INCLUDE<br>/EMMEANS=TABLES (SEX)<br>/EMMEANS=TABLES (GENOTYPE)<br>/PRINT ETASQ<br>/CRITERIA=ALPHA(.05)<br>/DESIGN=SEX GENOTYPE.
Resources	Processor Time	00:00:00.02
	Elapsed Time	00:00:00.00

Between-Subjects Factors

		N
SEX	F	14
	M	16
GENOTYPE	KI	15
	WT	15

Tests of Between-Subjects Effects

Dependent Variable: Day 2 Average

Source	Type III Sum of Squares	df	Mean Square	F	Sig.	Partial Eta Squared
Corrected Model	2896.138[a]	2	1448.069	.488	.619	.035
Intercept	1458875.03	1	1458875.03	491.710	<.001	.948
SEX	2894.938	1	2894.938	.976	.332	.035
GENOTYPE	1.200	1	1.200	.000	.984	.000
Error	80107.403	27	2966.941			
Total	1539700.67	30				
Corrected Total	83003.541	29				

a. R Squared = .035 (Adjusted R Squared = -.037)

Estimated Marginal Means

1. SEX

Dependent Variable: Day 2 Average

SEX	Mean	Std. Error	95% Confidence Interval Lower Bound	95% Confidence Interval Upper Bound
F	230.857	14.558	200.987	260.727
M	211.167	13.617	183.226	239.107

2. GENOTYPE

Dependent Variable: Day 2 Average

GENOTYPE	Mean	Std. Error	95% Confidence Interval Lower Bound	95% Confidence Interval Upper Bound
KI	221.212	14.080	192.323	250.101
WT	220.812	14.080	191.923	249.701

```
UNIANOVA Day3Average BY SEX GENOTYPE
/METHOD=SSTYPE(3)
/INTERCEPT=INCLUDE
/EMMEANS=TABLES(SEX)
/EMMEANS=TABLES(GENOTYPE)
/PRINT ETASQ
/CRITERIA=ALPHA(.05)
/DESIGN=SEX GENOTYPE.
```

Univariate Analysis of Variance

Notes

Output Created		19-AUG-2022 11:56:...
Comments		
Input	Data	/Users/jeffneul/Jeff's Research/Neul Manuscripts/FoxG1/June 2022 stat sex and effect size/SPSS files/SPSS Data Files/June 2022 RR sex EF data.sav
	Active Dataset	DataSet1
	Filter	<none>
	Weight	<none>
	Split File	<none>
	N of Rows in Working Data File	30
Missing Value Handling	Definition of Missing	User-defined missing values are treated as missing.
	Cases Used	Statistics are based on all cases with valid data for all variables in the model.
Syntax		UNIANOVA Day3Average BY SEX GENOTYPE<br>/METHOD=SSTYPE(3)<br>/INTERCEPT=INCLUDE<br>/EMMEANS=TABLES (SEX)<br>/EMMEANS=TABLES (GENOTYPE)<br>/PRINT ETASQ<br>/CRITERIA=ALPHA(.05)<br>/DESIGN=SEX GENOTYPE.
Resources	Processor Time	00:00:00.02
	Elapsed Time	00:00:00.00

Between-Subjects Factors

		N
SEX	F	14
	M	16
GENOTYPE	KI	15
	WT	15

Tests of Between-Subjects Effects

Dependent Variable: Day 3 Average

Source	Type III Sum of Squares	df	Mean Square	F	Sig.	Partial Eta Squared
Corrected Model	23414.397 [a]	2	11707.198	4.863	.016	.265
Intercept	1685785.74	1	1685785.74	700.199	<.001	.963
SEX	22613.564	1	22613.564	9.393	.005	.258
GENOTYPE	800.833	1	800.833	.333	.569	.012
Error	65004.644	27	2407.579			
Total	1755682.33	30				
Corrected Total	88419.041	29				

a. R Squared = .265 (Adjusted R Squared = .210)

Estimated Marginal Means

1. SEX

Dependent Variable: Day 3 Average

SEX	Mean	Std. Error	95% Confidence Interval Lower Bound	Upper Bound
F	265.095	13.114	238.188	292.002
M	210.063	12.267	184.893	235.232

2. GENOTYPE

Dependent Variable: Day 3 Average

GENOTYPE	Mean	Std. Error	95% Confidence Interval Lower Bound	Upper Bound
KI	232.412	12.683	206.388	258.436
WT	242.746	12.683	216.722	268.769

Open Field Analysis

Univariate Analysis of Variance

Notes

Output Created		19-AUG-2022 12:07:...
Comments		
Input	Data	/Users/jeffneul/Jeff's Research/Neul Manuscripts/FoxG1/June 2022 stat sex and effect size/SPSS files/SPSS Data Files/June 2022 OFA sex EF data.sav
	Active Dataset	DataSet2
	Filter	<none>
	Weight	<none>
	Split File	<none>
	N of Rows in Working Data File	57
Missing Value Handling	Definition of Missing	User-defined missing values are treated as missing.
	Cases Used	Statistics are based on all cases with valid data for all variables in the model.
Syntax		UNIANOVA TotalAmbulatoryDistance BY GENDER GENOTYPE /METHOD=SSTYPE(3) /INTERCEPT=INCLUDE /CRITERIA=ALPHA(.05) /DESIGN=GENDER GENOTYPE GENDER*GENOTYPE.
Resources	Processor Time	00:00:00.01
	Elapsed Time	00:00:00.00

Between-Subjects Factors

		N
GENDER	F	28
	M	29
GENOTYPE	KI	31
	WT	26

Tests of Between-Subjects Effects

Dependent Variable: Total Ambulatory Distance

Source	Type III Sum of Squares	df	Mean Square	F	Sig.
Corrected Model	14973286 [a]	3	4991095.18	3.451	.023
Intercept	998010650	1	998010650	690.018	<.001
GENDER	2997675.76	1	2997675.76	2.073	.156
GENOTYPE	11388810.0	1	11388810.0	7.874	.007
GENDER * GENOTYPE	1210068.15	1	1210068.15	.837	.365
Error	76656825.9	53	1446355.21		
Total	1.094E+9	57			
Corrected Total	91630111.4	56			

a. R Squared = .163 (Adjusted R Squared = .116)

```
UNIANOVA DistanceCenter BY GENDER GENOTYPE
/METHOD=SSTYPE(3)
/INTERCEPT=INCLUDE
/CRITERIA=ALPHA(.05)
/DESIGN=GENDER GENOTYPE GENDER*GENOTYPE.
```

Univariate Analysis of Variance

Notes

Output Created		19-AUG-2022 12:07:...
Comments		
Input	Data	/Users/jeffneul/Jeff's Research/Neul Manuscripts/FoxG1/June 2022 stat sex and effect size/SPSS files/SPSS Data Files/June 2022 OFA sex EF data.sav
	Active Dataset	DataSet2
	Filter	<none>
	Weight	<none>
	Split File	<none>
	N of Rows in Working Data File	57
Missing Value Handling	Definition of Missing	User-defined missing values are treated as missing.
	Cases Used	Statistics are based on all cases with valid data for all variables in the model.
Syntax		UNIANOVA DistanceCenter BY GENDER GENOTYPE /METHOD=SSTYPE(3) /INTERCEPT=INCLUDE /CRITERIA=ALPHA(.05) /DESIGN=GENDER GENOTYPE GENDER*GENOTYPE.
Resources	Processor Time	00:00:00.01
	Elapsed Time	00:00:00.00

Between-Subjects Factors

		N
GENDER	F	28
	M	29
GENOTYPE	KI	31
	WT	26

Tests of Between-Subjects Effects

Dependent Variable: % Distance Center

Source	Type III Sum of Squares	df	Mean Square	F	Sig.
Corrected Model	4028.722 [a]	3	1342.907	18.382	<.001
Intercept	166310.545	1	166310.545	2276.514	<.001
GENDER	5.660	1	5.660	.077	.782
GENOTYPE	3881.746	1	3881.746	53.135	<.001
GENDER * GENOTYPE	71.398	1	71.398	.977	.327
Error	3871.911	53	73.055		
Total	171631.297	57			
Corrected Total	7900.633	56			

a. R Squared = .510 (Adjusted R Squared = .482)

```
UNIANOVA TimeCenter BY GENDER GENOTYPE
/METHOD=SSTYPE(3)
/INTERCEPT=INCLUDE
/CRITERIA=ALPHA(.05)
/DESIGN=GENDER GENOTYPE GENDER*GENOTYPE.
```

Univariate Analysis of Variance

Notes

Output Created		19-AUG-2022 12:08:...
Comments		
Input	Data	/Users/jeffneul/Jeff's Research/Neul Manuscripts/FoxG1/June 2022 stat sex and effect size/SPSS files/SPSS Data Files/June 2022 OFA sex EF data.sav
	Active Dataset	DataSet2
	Filter	<none>
	Weight	<none>
	Split File	<none>
	N of Rows in Working Data File	57
Missing Value Handling	Definition of Missing	User-defined missing values are treated as missing.
	Cases Used	Statistics are based on all cases with valid data for all variables in the model.
Syntax		UNIANOVA TimeCenter BY GENDER GENOTYPE<br>/METHOD=SSTYPE(3)<br>/INTERCEPT=INCLUDE<br>/CRITERIA=ALPHA(.05)<br>/DESIGN=GENDER GENOTYPE GENDER*GENOTYPE.
Resources	Processor Time	00:00:00.01
	Elapsed Time	00:00:00.00

Between-Subjects Factors

		N
GENDER	F	28
	M	29
GENOTYPE	KI	31
	WT	26

Tests of Between-Subjects Effects

Dependent Variable: % Time Center

Source	Type III Sum of Squares	df	Mean Square	F	Sig.
Corrected Model	697.821[a]	3	232.607	2.388	.079
Intercept	133499.524	1	133499.524	1370.325	<.001
GENDER	.849	1	.849	.009	.926
GENOTYPE	669.918	1	669.918	6.876	.011
GENDER * GENOTYPE	15.062	1	15.062	.155	.696
Error	5163.357	53	97.422		
Total	139469.391	57			
Corrected Total	5861.178	56			

a. R Squared = .119 (Adjusted R Squared = .069)

```
UNIANOVA TotalVerticleCountsBY GENDER GENOTYPE
/METHOD=SSTYPE(3)
/INTERCEPT=INCLUDE
/CRITERIA=ALPHA(.05)
/DESIGN=GENDER GENOTYPE GENDER*GENOTYPE.
```

Univariate Analysis of Variance

Notes

Output Created		19-AUG-2022 12:09:...
Comments		
Input	Data	/Users/jeffneul/Jeff's Research/Neul Manuscripts/FoxG1/June 2022 stat sex and effect size/SPSS files/SPSS Data Files/June 2022 OFA sex EF data.sav
	Active Dataset	DataSet2
	Filter	<none>
	Weight	<none>
	Split File	<none>
	N of Rows in Working Data File	57
Missing Value Handling	Definition of Missing	User-defined missing values are treated as missing.
	Cases Used	Statistics are based on all cases with valid data for all variables in the model.
Syntax		UNIANOVA TotalVerticleCounts BY GENDER GENOTYPE<br>/METHOD=SSTYPE(3)<br>/INTERCEPT=INCLUDE<br>/CRITERIA=ALPHA(.05)<br>/DESIGN=GENDER GENOTYPE GENDER*GENOTYPE.
Resources	Processor Time	00:00:00.01
	Elapsed Time	00:00:00.00

Between-Subjects Factors

		N
GENDER	F	28
	M	29
GENOTYPE	KI	31
	WT	26

Tests of Between-Subjects Effects

Dependent Variable: Total Verticle Counts

Source	Type III Sum of Squares	df	Mean Square	F	Sig.
Corrected Model	154657.52 [a]	3	51552.505	5.383	.003
Intercept	4763390.28	1	4763390.28	497.418	<.001
GENDER	62762.270	1	62762.270	6.554	.013
GENOTYPE	65730.435	1	65730.435	6.864	.011
GENDER * GENOTYPE	29136.155	1	29136.155	3.043	.087
Error	507540.625	53	9576.238		
Total	5484942.00	57			
Corrected Total	662198.140	56			

a. R Squared = .234 (Adjusted R Squared = .190)

```
UNIANOVA TotalAmbulatoryDistance BY GENOTYPE GENDER
/METHOD=SSTYPE(3)
/INTERCEPT=INCLUDE
/EMMEANS=TABLES(GENOTYPE)
/EMMEANS=TABLES(GENDER)
/PRINT ETASQ
/CRITERIA=ALPHA(.05)
/DESIGN=GENOTYPE GENDER.
```

Univariate Analysis of Variance

Notes

Output Created		19-AUG-2022 12:12:...
Comments		
Input	Data	/Users/jeffneul/Jeff's Research/Neul Manuscripts/FoxG1/June 2022 stat sex and effect size/SPSS files/SPSS Data Files/June 2022 OFA sex EF data.sav
	Active Dataset	DataSet2
	Filter	<none>
	Weight	<none>
	Split File	<none>
	N of Rows in Working Data File	57
Missing Value Handling	Definition of Missing	User-defined missing values are treated as missing.
	Cases Used	Statistics are based on all cases with valid data for all variables in the model.
Syntax		UNIANOVA TotalAmbulatoryDistance BY GENOTYPE GENDER /METHOD=SSTYPE(3) /INTERCEPT=INCLUDE /EMMEANS=TABLES (GENOTYPE) /EMMEANS=TABLES (GENDER) /PRINT ETASQ /CRITERIA=ALPHA(.05) /DESIGN=GENOTYPE GENDER.
Resources	Processor Time	00:00:00.01
	Elapsed Time	00:00:00.00

Between-Subjects Factors

		N
GENOTYPE	KI	31
	WT	26
GENDER	F	28
	M	29

Tests of Between-Subjects Effects

Dependent Variable: Total Ambulatory Distance

Source	Type III Sum of Squares	df	Mean Square	F	Sig.	Partial Eta Squared
Corrected Model	13763217 [a]	2	6881608.70	4.772	.012	.150
Intercept	1.011E+9	1	1.011E+9	701.450	<.001	.929
GENOTYPE	11370643.6	1	11370643.6	7.885	.007	.127
GENDER	3359032.52	1	3359032.52	2.329	.133	.041
Error	77866894.0	54	1441979.52			
Total	1.094E+9	57				
Corrected Total	91630111.4	56				

a. R Squared = .150 (Adjusted R Squared = .119)

Estimated Marginal Means

1. GENOTYPE

Dependent Variable: Total Ambulatory Distance

GENOTYPE	Mean	Std. Error	95% Confidence Interval	
			Lower Bound	Upper Bound
KI	3778.961	216.227	3345.451	4212.471
WT	4679.069	235.821	4206.276	5151.861

2. GENDER

Dependent Variable: Total Ambulatory Distance

GENDER	Mean	Std. Error	95% Confidence Interval	
			Lower Bound	Upper Bound
F	3985.308	226.935	3530.331	4440.284
M	4472.722	224.693	4022.239	4923.205

```
UNIANOVA DistanceCenter BY GENOTYPE GENDER
/METHOD=SSTYPE(3)
/INTERCEPT=INCLUDE
/EMMEANS=TABLES(GENOTYPE)
/EMMEANS=TABLES(GENDER)
/PRINT ETASQ
/CRITERIA=ALPHA(.05)
/DESIGN=GENOTYPE GENDER.
```

Univariate Analysis of Variance

Notes

Output Created		19-AUG-2022 12:12:...
Comments		
Input	Data	/Users/jeffneul/Jeff's Research/Neul Manuscripts/FoxG1/June 2022 stat sex and effect size/SPSS files/SPSS Data Files/June 2022 OFA sex EF data.sav
	Active Dataset	DataSet2
	Filter	<none>
	Weight	<none>
	Split File	<none>
	N of Rows in Working Data File	57
Missing Value Handling	Definition of Missing	User-defined missing values are treated as missing.
	Cases Used	Statistics are based on all cases with valid data for all variables in the model.
Syntax		UNIANOVA DistanceCenter BY GENOTYPE GENDER<br>/METHOD=SSTYPE(3)<br>/INTERCEPT=INCLUDE<br>/EMMEANS=TABLES (GENOTYPE)<br>/EMMEANS=TABLES (GENDER)<br>/PRINT ETASQ<br>/CRITERIA=ALPHA(.05)<br>/DESIGN=GENOTYPE GENDER.
Resources	Processor Time	00:00:00.02
	Elapsed Time	00:00:00.00

Between-Subjects Factors

		N
GENOTYPE	KI	31
	WT	26
GENDER	F	28
	M	29

Tests of Between-Subjects Effects

Dependent Variable: % Distance Center

Source	Type III Sum of Squares	df	Mean Square	F	Sig.	Partial Eta Squared
Corrected Model	3957.325[a]	2	1978.662	27.096	<.001	.501
Intercept	166953.057	1	166953.057	2286.269	<.001	.977
GENOTYPE	3884.357	1	3884.357	53.193	<.001	.496
GENDER	9.718	1	9.718	.133	.717	.002
Error	3943.309	54	73.024			
Total	171631.297	57				
Corrected Total	7900.633	56				

a. R Squared = .501 (Adjusted R Squared = .482)

Estimated Marginal Means

1. GENOTYPE

Dependent Variable: % Distance Center

GENOTYPE	Mean	Std. Error	95% Confidence Interval Lower Bound	95% Confidence Interval Upper Bound
KI	46.014	1.539	42.929	49.099
WT	62.651	1.678	59.286	66.015

2. GENDER

Dependent Variable: % Distance Center

GENDER	Mean	Std. Error	95% Confidence Interval Lower Bound	95% Confidence Interval Upper Bound
F	54.747	1.615	51.509	57.985
M	53.918	1.599	50.712	57.124

```
UNIANOVA TimeCenter BY GENOTYPE GENDER
/METHOD=SSTYPE(3)
/INTERCEPT=INCLUDE
/EMMEANS=TABLES(GENOTYPE)
/EMMEANS=TABLES(GENDER)
/PRINT ETASQ
/CRITERIA=ALPHA(.05)
/DESIGN=GENOTYPE GENDER.
```

Univariate Analysis of Variance

Notes

Output Created		19-AUG-2022 12:13:...
Comments		
Input	Data	/Users/jeffneul/Jeff's Research/Neul Manuscripts/FoxG1/June 2022 stat sex and effect size/SPSS files/SPSS Data Files/June 2022 OFA sex EF data.sav
	Active Dataset	DataSet2
	Filter	<none>
	Weight	<none>
	Split File	<none>
	N of Rows in Working Data File	57
Missing Value Handling	Definition of Missing	User-defined missing values are treated as missing.
	Cases Used	Statistics are based on all cases with valid data for all variables in the model.
Syntax		UNIANOVA TimeCenter BY GENOTYPE GENDER<br>/METHOD=SSTYPE(3)<br>/INTERCEPT=INCLUDE<br>/EMMEANS=TABLES (GENOTYPE)<br>/EMMEANS=TABLES (GENDER)<br>/PRINT ETASQ<br>/CRITERIA=ALPHA(.05)<br>/DESIGN=GENOTYPE GENDER.
Resources	Processor Time	00:00:00.02
	Elapsed Time	00:00:00.00

Between-Subjects Factors

		N
GENOTYPE	KI	31
	WT	26
GENDER	F	28
	M	29

Tests of Between-Subjects Effects

Dependent Variable: % Time Center

Source	Type III Sum of Squares	df	Mean Square	F	Sig.	Partial Eta Squared
Corrected Model	682.759[a]	2	341.380	3.560	.035	.116
Intercept	134248.538	1	134248.538	1399.929	<.001	.963
GENOTYPE	670.416	1	670.416	6.991	.011	.115
GENDER	1.587	1	1.587	.017	.898	.000
Error	5178.419	54	95.897			
Total	139469.391	57				
Corrected Total	5861.178	56				

a. R Squared = .116 (Adjusted R Squared = .084)

Estimated Marginal Means

1. GENOTYPE

Dependent Variable: % Time Center

GENOTYPE	Mean	Std. Error	95% Confidence Interval Lower Bound	95% Confidence Interval Upper Bound
KI	45.265	1.763	41.730	48.800
WT	52.177	1.923	48.321	56.032

2. GENDER

Dependent Variable: % Time Center

GENDER	Mean	Std. Error	95% Confidence Interval Lower Bound	95% Confidence Interval Upper Bound
F	48.888	1.851	45.178	52.599
M	48.553	1.832	44.880	52.227

```
UNIANOVA TotalVerticleCountsBY GENOTYPE GENDER
/METHOD=SSTYPE(3)
/INTERCEPT=INCLUDE
/EMMEANS=TABLES(GENOTYPE)
/EMMEANS=TABLES(GENDER)
/PRINT ETASQ
/CRITERIA=ALPHA(.05)
/DESIGN=GENOTYPE GENDER.
```

Univariate Analysis of Variance

Notes

Output Created		19-AUG-2022 12:13:...
Comments		
Input	Data	/Users/jeffneul/Jeff's Research/Neul Manuscripts/FoxG1/June 2022 stat sex and effect size/SPSS files/SPSS Data Files/June 2022 OFA sex EF data.sav
	Active Dataset	DataSet2
	Filter	<none>
	Weight	<none>
	Split File	<none>
	N of Rows in Working Data File	57
Missing Value Handling	Definition of Missing	User-defined missing values are treated as missing.
	Cases Used	Statistics are based on all cases with valid data for all variables in the model.
Syntax		UNIANOVA TotalVerticleCounts BY GENOTYPE GENDER /METHOD=SSTYPE(3) /INTERCEPT=INCLUDE /EMMEANS=TABLES (GENOTYPE) /EMMEANS=TABLES (GENDER) /PRINT ETASQ /CRITERIA=ALPHA(.05) /DESIGN=GENOTYPE GENDER.
Resources	Processor Time	00:00:00.01
	Elapsed Time	00:00:00.00

Between-Subjects Factors

		N
GENOTYPE	KI	31
	WT	26
GENDER	F	28
	M	29

Tests of Between-Subjects Effects

Dependent Variable: Total Verticle Counts

Source	Type III Sum of Squares	df	Mean Square	F	Sig.	Partial Eta Squared
Corrected Model	125521.36 [a]	2	62760.680	6.315	.003	.190
Intercept	4863646.57	1	4863646.57	489.376	<.001	.901
GENOTYPE	65515.959	1	65515.959	6.592	.013	.109
GENDER	70856.500	1	70856.500	7.130	.010	.117
Error	536676.780	54	9938.459			
Total	5484942.00	57				
Corrected Total	662198.140	56				

a. R Squared = .190 (Adjusted R Squared = .160)

Estimated Marginal Means

1. GENOTYPE

Dependent Variable: Total Verticle Counts

GENOTYPE	Mean	Std. Error	95% Confidence Interval Lower Bound	95% Confidence Interval Upper Bound
KI	259.091	17.951	223.101	295.080
WT	327.415	19.578	288.164	366.666

2. GENDER

Dependent Variable: Total Verticle Counts

GENDER	Mean	Std. Error	95% Confidence Interval Lower Bound	95% Confidence Interval Upper Bound
F	257.857	18.840	220.085	295.629
M	328.649	18.654	291.250	366.047

Elevated Zero Maze

```
UNIANOVA DistanceTotal BY GENOTYPE SEX
/METHOD=SSTYPE(3)
/INTERCEPT=INCLUDE
/CRITERIA=ALPHA(0.05)
/DESIGN=GENOTYPE SEX GENOTYPE*SEX.
```

Univariate Analysis of Variance

Notes

Output Created		22-AUG-2022 14:39:...
Comments		
Input	Data	/Users/jeffneul/Jeff's Research/Neul Manuscripts/FoxG1/June 2022 stat sex and effect size/SPSS files/SPSS Data Files/June 2022 EZM sex EF data.sav
	Active Dataset	DataSet3
	Filter	<none>
	Weight	<none>
	Split File	<none>
	N of Rows in Working Data File	25
Missing Value Handling	Definition of Missing	User-defined missing values are treated as missing.
	Cases Used	Statistics are based on all cases with valid data for all variables in the model.
Syntax		UNIANOVA DistanceTotal BY GENOTYPE SEX /METHOD=SSTYPE(3) /INTERCEPT=INCLUDE /CRITERIA=ALPHA(0.05) /DESIGN=GENOTYPE SEX GENOTYPE*SEX.
Resources	Processor Time	00:00:00.01
	Elapsed Time	00:00:00.00

```
[DataSet3] /Users/jeffneul/Jeff's Research/Neul Manuscripts/FoxG1/June 2022 st
at sex and effect size/SPSS files/SPSS Data Files/June 2022 EZM sex EF data.sa
v
```

Between-Subjects Factors

		N
GENOTYPE	KI	14
	WT	11
SEX	F	16
	M	9

Tests of Between-Subjects Effects

Dependent Variable: Distance (Total)

Source	Type III Sum of Squares	df	Mean Square	F	Sig.
Corrected Model	22.276[a]	3	7.425	.252	.859
Intercept	8155.817	1	8155.817	277.014	<.001
GENOTYPE	2.968	1	2.968	.101	.754
SEX	.353	1	.353	.012	.914
GENOTYPE * SEX	12.086	1	12.086	.410	.529
Error	618.279	21	29.442		
Total	10188.816	25			
Corrected Total	640.556	24			

a. R Squared = .035 (Adjusted R Squared = -.103)

```
UNIANOVA TimeOpen BY GENOTYPE SEX
/METHOD=SSTYPE(3)
/INTERCEPT=INCLUDE
/CRITERIA=ALPHA(0.05)
/DESIGN=GENOTYPE SEX GENOTYPE*SEX.
```

Univariate Analysis of Variance

Notes

Output Created		22-AUG-2022 14:40:...
Comments		
Input	Data	/Users/jeffneul/Jeff's Research/Neul Manuscripts/FoxG1/June 2022 stat sex and effect size/SPSS files/SPSS Data Files/June 2022 EZM sex EF data.sav
	Active Dataset	DataSet3
	Filter	<none>
	Weight	<none>
	Split File	<none>
	N of Rows in Working Data File	25
Missing Value Handling	Definition of Missing	User-defined missing values are treated as missing.
	Cases Used	Statistics are based on all cases with valid data for all variables in the model.
Syntax		UNIANOVA TimeOpen BY GENOTYPE SEX /METHOD=SSTYPE(3) /INTERCEPT=INCLUDE /CRITERIA=ALPHA (0.05) /DESIGN=GENOTYPE SEX GENOTYPE*SEX.
Resources	Processor Time	00:00:00.01
	Elapsed Time	00:00:00.00

Between-Subjects Factors

		N
GENOTYPE	KI	14
	WT	11
SEX	F	16
	M	9

Tests of Between-Subjects Effects

Dependent Variable: % Time Open

Source	Type III Sum of Squares	df	Mean Square	F	Sig.
Corrected Model	173.520[a]	3	57.840	.380	.769
Intercept	48738.508	1	48738.508	320.040	<.001
GENOTYPE	101.012	1	101.012	.663	.425
SEX	.477	1	.477	.003	.956
GENOTYPE * SEX	20.439	1	20.439	.134	.718
Error	3198.067	21	152.289		
Total	60730.510	25			
Corrected Total	3371.588	24			

a. R Squared = .051 (Adjusted R Squared = -.084)

```
UNIANOVA DistanceOpen BY GENOTYPE SEX
/METHOD=SSTYPE(3)
/INTERCEPT=INCLUDE
/CRITERIA=ALPHA(0.05)
/DESIGN=GENOTYPE SEX GENOTYPE*SEX.
```

Univariate Analysis of Variance

Notes

Output Created		22-AUG-2022 14:40:...
Comments		
Input	Data	/Users/jeffneul/Jeff's Research/Neul Manuscripts/FoxG1/June 2022 stat sex and effect size/SPSS files/SPSS Data Files/June 2022 EZM sex EF data.sav
	Active Dataset	DataSet3
	Filter	<none>
	Weight	<none>
	Split File	<none>
	N of Rows in Working Data File	25
Missing Value Handling	Definition of Missing	User-defined missing values are treated as missing.
	Cases Used	Statistics are based on all cases with valid data for all variables in the model.
Syntax		UNIANOVA DistanceOpen BY GENOTYPE SEX /METHOD=SSTYPE(3) /INTERCEPT=INCLUDE /CRITERIA=ALPHA (0.05) /DESIGN=GENOTYPE SEX GENOTYPE*SEX.
Resources	Processor Time	00:00:00.01
	Elapsed Time	00:00:00.00

Between-Subjects Factors

		N
GENOTYPE	KI	14
	WT	11
SEX	F	16
	M	9

Tests of Between-Subjects Effects

Dependent Variable: % Distance Open

Source	Type III Sum of Squares	df	Mean Square	F	Sig.
Corrected Model	503.264[a]	3	167.755	3.849	.024
Intercept	41539.772	1	41539.772	953.202	<.001
GENOTYPE	294.039	1	294.039	6.747	.017
SEX	3.118	1	3.118	.072	.792
GENOTYPE * SEX	56.483	1	56.483	1.296	.268
Error	915.163	21	43.579		
Total	50414.320	25			
Corrected Total	1418.427	24			

a. R Squared = .355 (Adjusted R Squared = .263)

```
UNIANOVA DistanceTotal BY GENOTYPE SEX
/METHOD=SSTYPE(3)
/INTERCEPT=INCLUDE
/EMMEANS=TABLES(GENOTYPE)
/PRINT ETASQ
/CRITERIA=ALPHA(.05)
/DESIGN=GENOTYPE SEX.
```

Univariate Analysis of Variance

Notes

Output Created		22-AUG-2022 14:42:...
Comments		
Input	Data	/Users/jeffneul/Jeff's Research/Neul Manuscripts/FoxG1/June 2022 stat sex and effect size/SPSS files/SPSS Data Files/June 2022 EZM sex EF data.sav
	Active Dataset	DataSet3
	Filter	<none>
	Weight	<none>
	Split File	<none>
	N of Rows in Working Data File	25
Missing Value Handling	Definition of Missing	User-defined missing values are treated as missing.
	Cases Used	Statistics are based on all cases with valid data for all variables in the model.
Syntax		UNIANOVA DistanceTotal BY GENOTYPE SEX<br>/METHOD=SSTYPE(3)<br>/INTERCEPT=INCLUDE<br>/EMMEANS=TABLES (GENOTYPE)<br>/PRINT ETASQ<br>/CRITERIA=ALPHA(.05)<br>/DESIGN=GENOTYPE SEX.
Resources	Processor Time	00:00:00.01
	Elapsed Time	00:00:00.00

Between-Subjects Factors

		N
GENOTYPE	KI	14
	WT	11
SEX	F	16
	M	9

Tests of Between-Subjects Effects

Dependent Variable: Distance (Total)

Source	Type III Sum of Squares	df	Mean Square	F	Sig.	Partial Eta Squared
Corrected Model	10.190[a]	2	5.095	.178	.838	.016
Intercept	8433.338	1	8433.338	294.327	<.001	.930
GENOTYPE	9.342	1	9.342	.326	.574	.015
SEX	1.964	1	1.964	.069	.796	.003
Error	630.365	22	28.653			
Total	10188.816	25				
Corrected Total	640.556	24				

a. R Squared = .016 (Adjusted R Squared = -.074)

Estimated Marginal Means

GENOTYPE

Dependent Variable: Distance (Total)

GENOTYPE	Mean	Std. Error	95% Confidence Interval	
			Lower Bound	Upper Bound
KI	20.009	1.440	17.024	22.995
WT	18.761	1.694	15.249	22.274

```
UNIANOVA TimeOpen BY GENOTYPE SEX
/METHOD=SSTYPE(3)
/INTERCEPT=INCLUDE
/EMMEANS=TABLES(GENOTYPE)
/PRINT ETASQ
/CRITERIA=ALPHA(.05)
/DESIGN=GENOTYPE SEX.
```

Univariate Analysis of Variance

Notes

Output Created		22-AUG-2022 14:42:...
Comments		
Input	Data	/Users/jeffneul/Jeff's Research/Neul Manuscripts/FoxG1/June 2022 stat sex and effect size/SPSS files/SPSS Data Files/June 2022 EZM sex EF data.sav
	Active Dataset	DataSet3
	Filter	<none>
	Weight	<none>
	Split File	<none>
	N of Rows in Working Data File	25
Missing Value Handling	Definition of Missing	User-defined missing values are treated as missing.
	Cases Used	Statistics are based on all cases with valid data for all variables in the model.
Syntax		UNIANOVA TimeOpen BY GENOTYPE SEX<br>/METHOD=SSTYPE(3)<br>/INTERCEPT=INCLUDE<br>/EMMEANS=TABLES (GENOTYPE)<br>/PRINT ETASQ<br>/CRITERIA=ALPHA(.05)<br>/DESIGN=GENOTYPE SEX.
Resources	Processor Time	00:00:00.02
	Elapsed Time	00:00:00.00

Between-Subjects Factors

		N
GENOTYPE	KI	14
	WT	11
SEX	F	16
	M	9

Tests of Between-Subjects Effects

Dependent Variable: % Time Open

Source	Type III Sum of Squares	df	Mean Square	F	Sig.	Partial Eta Squared
Corrected Model	153.081[a]	2	76.540	.523	.600	.045
Intercept	50804.801	1	50804.801	347.275	<.001	.940
GENOTYPE	150.272	1	150.272	1.027	.322	.045
SEX	.104	1	.104	.001	.979	.000
Error	3218.507	22	146.296			
Total	60730.510	25				
Corrected Total	3371.588	24				

a. R Squared = .045 (Adjusted R Squared = -.041)

Estimated Marginal Means

GENOTYPE

Dependent Variable: % Time Open

GENOTYPE	Mean	Std. Error	95% Confidence Interval	
			Lower Bound	Upper Bound
KI	50.082	3.253	43.336	56.829
WT	45.078	3.827	37.141	53.015

```
UNIANOVA DistanceOpen BY GENOTYPE SEX
/METHOD=SSTYPE(3)
/INTERCEPT=INCLUDE
/EMMEANS=TABLES(GENOTYPE)
/PRINT ETASQ
/CRITERIA=ALPHA(.05)
/DESIGN=GENOTYPE SEX.
```

Univariate Analysis of Variance

Notes

Output Created		22-AUG-2022 14:43:...
Comments		
Input	Data	/Users/jeffneul/Jeff's Research/Neul Manuscripts/FoxG1/June 2022 stat sex and effect size/SPSS files/SPSS Data Files/June 2022 EZM sex EF data.sav
	Active Dataset	DataSet3
	Filter	<none>
	Weight	<none>
	Split File	<none>
	N of Rows in Working Data File	25
Missing Value Handling	Definition of Missing	User-defined missing values are treated as missing.
	Cases Used	Statistics are based on all cases with valid data for all variables in the model.
Syntax		UNIANOVA DistanceOpen BY GENOTYPE SEX<br>/METHOD=SSTYPE(3)<br>/INTERCEPT=INCLUDE<br>/EMMEANS=TABLES (GENOTYPE)<br>/PRINT ETASQ<br>/CRITERIA=ALPHA(.05)<br>/DESIGN=GENOTYPE SEX.
Resources	Processor Time	00:00:00.02
	Elapsed Time	00:00:00.00

Between-Subjects Factors

		N
GENOTYPE	KI	14
	WT	11
SEX	F	16
	M	9

Tests of Between-Subjects Effects

Dependent Variable: % Distance Open

Source	Type III Sum of Squares	df	Mean Square	F	Sig.	Partial Eta Squared
Corrected Model	446.782[a]	2	223.391	5.058	.016	.315
Intercept	42984.470	1	42984.470	973.254	<.001	.978
GENOTYPE	434.508	1	434.508	9.838	.005	.309
SEX	.010	1	.010	.000	.988	.000
Error	971.646	22	44.166			
Total	50414.320	25				
Corrected Total	1418.427	24				

a. R Squared = .315 (Adjusted R Squared = .253)

Estimated Marginal Means

GENOTYPE

Dependent Variable: % Distance Open

GENOTYPE	Mean	Std. Error	95% Confidence Interval	
			Lower Bound	Upper Bound
KI	48.020	1.787	44.313	51.727
WT	39.510	2.103	35.149	43.871

Gait

```
UNIANOVA ForepawStridems BY GENOTYPE SEX
  /METHOD=SSTYPE(3)
  /INTERCEPT=INCLUDE
  /CRITERIA=ALPHA(.05)
  /DESIGN=GENOTYPE SEX GENOTYPE*SEX.
```

Univariate Analysis of Variance

Notes

Output Created		22-AUG-2022 14:52:...
Comments		
Input	Data	/Users/jeffneul/Jeff's Research/Neul Manuscripts/FoxG1/June 2022 stat sex and effect size/SPSS files/SPSS Data Files/June 2022 Gait sex EF data.sav
	Active Dataset	DataSet4
	Filter	<none>
	Weight	<none>
	Split File	<none>
	N of Rows in Working Data File	56
Missing Value Handling	Definition of Missing	User-defined missing values are treated as missing.
	Cases Used	Statistics are based on all cases with valid data for all variables in the model.
Syntax		UNIANOVA ForepawStridems BY GENOTYPE SEX /METHOD=SSTYPE(3) /INTERCEPT=INCLUDE /CRITERIA=ALPHA(.05) /DESIGN=GENOTYPE SEX GENOTYPE*SEX.
Resources	Processor Time	00:00:00.01
	Elapsed Time	00:00:00.00

Between-Subjects Factors

		N
GENOTYPE	KI	30
	WT	26
SEX	F	27
	M	29

Tests of Between-Subjects Effects

Dependent Variable: ForepawStride (ms)

Source	Type III Sum of Squares	df	Mean Square	F	Sig.
Corrected Model	3561.621 [a]	3	1187.207	1.379	.259
Intercept	4144921.88	1	4144921.88	4815.880	<.001
GENOTYPE	2376.838	1	2376.838	2.762	.103
SEX	1084.747	1	1084.747	1.260	.267
GENOTYPE * SEX	431.261	1	431.261	.501	.482
Error	44755.258	52	860.678		
Total	4244051.19	56			
Corrected Total	48316.879	55			

a. R Squared = .074 (Adjusted R Squared = .020)

```
UNIANOVA ForepawPercofSwing BY GENOTYPE SEX
/METHOD=SSTYPE(3)
/INTERCEPT=INCLUDE
/CRITERIA=ALPHA(.05)
/DESIGN=GENOTYPE SEX GENOTYPE*SEX.
```

Univariate Analysis of Variance

Notes

Output Created		22-AUG-2022 14:53:...
Comments		
Input	Data	/Users/jeffneul/Jeff's Research/Neul Manuscripts/FoxG1/June 2022 stat sex and effect size/SPSS files/SPSS Data Files/June 2022 Gait sex EF data.sav
	Active Dataset	DataSet4
	Filter	<none>
	Weight	<none>
	Split File	<none>
	N of Rows in Working Data File	56
Missing Value Handling	Definition of Missing	User-defined missing values are treated as missing.
	Cases Used	Statistics are based on all cases with valid data for all variables in the model.
Syntax		UNIANOVA ForepawPercofSwing BY GENOTYPE SEX /METHOD=SSTYPE(3) /INTERCEPT=INCLUDE /CRITERIA=ALPHA(.05) /DESIGN=GENOTYPE SEX GENOTYPE*SEX.
Resources	Processor Time	00:00:00.01
	Elapsed Time	00:00:00.00

Between-Subjects Factors

		N
GENOTYPE	KI	30
	WT	26
SEX	F	27
	M	29

Tests of Between-Subjects Effects

Dependent Variable: ForepawPercofSwing

Source	Type III Sum of Squares	df	Mean Square	F	Sig.
Corrected Model	.008[a]	3	.003	2.463	.073
Intercept	14.066	1	14.066	12499.957	<.001
GENOTYPE	.008	1	.008	7.036	.011
SEX	.001	1	.001	.735	.395
GENOTYPE * SEX	6.835E-6	1	6.835E-6	.006	.938
Error	.059	52	.001		
Total	14.412	56			
Corrected Total	.067	55			

a. R Squared = .124 (Adjusted R Squared = .074)

```
UNIANOVA ForepawStrideLenmmBY GENOTYPE SEX
/METHOD=SSTYPE(3)
/INTERCEPT=INCLUDE
/CRITERIA=ALPHA(.05)
/DESIGN=GENOTYPE SEX GENOTYPE*SEX.
```

Univariate Analysis of Variance

Notes

Output Created		22-AUG-2022 14:53:...
Comments		
Input	Data	/Users/jeffneul/Jeff's Research/Neul Manuscripts/FoxG1/June 2022 stat sex and effect size/SPSS files/SPSS Data Files/June 2022 Gait sex EF data.sav
	Active Dataset	DataSet4
	Filter	<none>
	Weight	<none>
	Split File	<none>
	N of Rows in Working Data File	56
Missing Value Handling	Definition of Missing	User-defined missing values are treated as missing.
	Cases Used	Statistics are based on all cases with valid data for all variables in the model.
Syntax		UNIANOVA ForepawStrideLenmm BY GENOTYPE SEX /METHOD=SSTYPE(3) /INTERCEPT=INCLUDE /CRITERIA=ALPHA(.05) /DESIGN=GENOTYPE SEX GENOTYPE*SEX.
Resources	Processor Time	00:00:00.01
	Elapsed Time	00:00:01.00

Between-Subjects Factors

		N
GENOTYPE	KI	30
	WT	26
SEX	F	27
	M	29

Tests of Between-Subjects Effects

Dependent Variable: ForepawStrideLen (mm)

Source	Type III Sum of Squares	df	Mean Square	F	Sig.
Corrected Model	68.917[a]	3	22.972	1.776	.163
Intercept	195002.616	1	195002.616	15072.242	<.001
GENOTYPE	24.871	1	24.871	1.922	.172
SEX	27.970	1	27.970	2.162	.147
GENOTYPE * SEX	13.376	1	13.376	1.034	.314
Error	672.769	52	12.938		
Total	199154.050	56			
Corrected Total	741.686	55			

a. R Squared = .093 (Adjusted R Squared = .041)

```
UNIANOVA ForepawAvPrintAreaBY GENOTYPE SEX
/METHOD=SSTYPE(3)
/INTERCEPT=INCLUDE
/CRITERIA=ALPHA(.05)
/DESIGN=GENOTYPE SEX GENOTYPE*SEX.
```

Univariate Analysis of Variance

Notes

Output Created		22-AUG-2022 14:53:...
Comments		
Input	Data	/Users/jeffneul/Jeff's Research/Neul Manuscripts/FoxG1/June 2022 stat sex and effect size/SPSS files/SPSS Data Files/June 2022 Gait sex EF data.sav
	Active Dataset	DataSet4
	Filter	<none>
	Weight	<none>
	Split File	<none>
	N of Rows in Working Data File	56
Missing Value Handling	Definition of Missing	User-defined missing values are treated as missing.
	Cases Used	Statistics are based on all cases with valid data for all variables in the model.
Syntax		UNIANOVA ForepawAvPrintArea BY GENOTYPE SEX /METHOD=SSTYPE(3) /INTERCEPT=INCLUDE /CRITERIA=ALPHA(.05) /DESIGN=GENOTYPE SEX GENOTYPE*SEX.
Resources	Processor Time	00:00:00.01
	Elapsed Time	00:00:00.00

Between-Subjects Factors

		N
GENOTYPE	KI	30
	WT	26
SEX	F	27
	M	29

Tests of Between-Subjects Effects

Dependent Variable: ForepawAvPrintArea

Source	Type III Sum of Squares	df	Mean Square	F	Sig.
Corrected Model	8712.657[a]	3	2904.219	3.665	.018
Intercept	1131707.78	1	1131707.78	1428.281	<.001
GENOTYPE	5160.165	1	5160.165	6.512	.014
SEX	4448.738	1	4448.738	5.615	.022
GENOTYPE * SEX	3.242	1	3.242	.004	.949
Error	41202.552	52	792.357		
Total	1194257.43	56			
Corrected Total	49915.208	55			

a. R Squared = .175 (Adjusted R Squared = .127)

```
UNIANOVA HindpawStridems BY GENOTYPE SEX
/METHOD=SSTYPE(3)
/INTERCEPT=INCLUDE
/CRITERIA=ALPHA(.05)
/DESIGN=GENOTYPE SEX GENOTYPE*SEX.
```

Univariate Analysis of Variance

Notes

Output Created		22-AUG-2022 14:54:...
Comments		
Input	Data	/Users/jeffneul/Jeff's Research/Neul Manuscripts/FoxG1/June 2022 stat sex and effect size/SPSS files/SPSS Data Files/June 2022 Gait sex EF data.sav
	Active Dataset	DataSet4
	Filter	<none>
	Weight	<none>
	Split File	<none>
	N of Rows in Working Data File	56
Missing Value Handling	Definition of Missing	User-defined missing values are treated as missing.
	Cases Used	Statistics are based on all cases with valid data for all variables in the model.
Syntax		UNIANOVA HindpawStridems BY GENOTYPE SEX /METHOD=SSTYPE(3) /INTERCEPT=INCLUDE /CRITERIA=ALPHA(.05) /DESIGN=GENOTYPE SEX GENOTYPE*SEX.
Resources	Processor Time	00:00:00.01
	Elapsed Time	00:00:00.00

Between-Subjects Factors

		N
GENOTYPE	KI	30
	WT	26
SEX	F	27
	M	29

Tests of Between-Subjects Effects

Dependent Variable: HindpawStride (ms)

Source	Type III Sum of Squares	df	Mean Square	F	Sig.
Corrected Model	942.235[a]	3	314.078	.354	.787
Intercept	4435616.86	1	4435616.86	4997.572	<.001
GENOTYPE	21.808	1	21.808	.025	.876
SEX	198.873	1	198.873	.224	.638
GENOTYPE * SEX	644.920	1	644.920	.727	.398
Error	46152.831	52	887.554		
Total	4541914.50	56			
Corrected Total	47095.066	55			

a. R Squared = .020 (Adjusted R Squared = -.037)

```
UNIANOVA HindpawPercofSwingBY GENOTYPE SEX
/METHOD=SSTYPE(3)
/INTERCEPT=INCLUDE
/CRITERIA=ALPHA(.05)
/DESIGN=GENOTYPE SEX GENOTYPE*SEX.
```

Univariate Analysis of Variance

Notes

Output Created		22-AUG-2022 14:54:...
Comments		
Input	Data	/Users/jeffneul/Jeff's Research/Neul Manuscripts/FoxG1/June 2022 stat sex and effect size/SPSS files/SPSS Data Files/June 2022 Gait sex EF data.sav
	Active Dataset	DataSet4
	Filter	<none>
	Weight	<none>
	Split File	<none>
	N of Rows in Working Data File	56
Missing Value Handling	Definition of Missing	User-defined missing values are treated as missing.
	Cases Used	Statistics are based on all cases with valid data for all variables in the model.
Syntax		UNIANOVA HindpawPercofSwing BY GENOTYPE SEX /METHOD=SSTYPE(3) /INTERCEPT=INCLUDE /CRITERIA=ALPHA(.05) /DESIGN=GENOTYPE SEX GENOTYPE*SEX.
Resources	Processor Time	00:00:00.01
	Elapsed Time	00:00:00.00

Between-Subjects Factors

		N
GENOTYPE	KI	30
	WT	26
SEX	F	27
	M	29

Tests of Between-Subjects Effects

Dependent Variable: HindpawPercofSwing

Source	Type III Sum of Squares	df	Mean Square	F	Sig.
Corrected Model	.026[a]	3	.009	4.644	.006
Intercept	16.573	1	16.573	8775.500	<.001
GENOTYPE	.022	1	.022	11.629	.001
SEX	.004	1	.004	2.130	.150
GENOTYPE * SEX	.002	1	.002	1.064	.307
Error	.098	52	.002		
Total	17.084	56			
Corrected Total	.125	55			

a. R Squared = .211 (Adjusted R Squared = .166)

```
UNIANOVA HindpawStrideLenmmBY GENOTYPE SEX
/METHOD=SSTYPE(3)
/INTERCEPT=INCLUDE
/CRITERIA=ALPHA(.05)
/DESIGN=GENOTYPE SEX GENOTYPE*SEX.
```

Univariate Analysis of Variance

Notes

Output Created		22-AUG-2022 14:55:...
Comments		
Input	Data	/Users/jeffneul/Jeff's Research/Neul Manuscripts/FoxG1/June 2022 stat sex and effect size/SPSS files/SPSS Data Files/June 2022 Gait sex EF data.sav
	Active Dataset	DataSet4
	Filter	<none>
	Weight	<none>
	Split File	<none>
	N of Rows in Working Data File	56
Missing Value Handling	Definition of Missing	User-defined missing values are treated as missing.
	Cases Used	Statistics are based on all cases with valid data for all variables in the model.
Syntax		UNIANOVA HindpawStrideLenmm BY GENOTYPE SEX /METHOD=SSTYPE(3) /INTERCEPT=INCLUDE /CRITERIA=ALPHA(.05) /DESIGN=GENOTYPE SEX GENOTYPE*SEX.
Resources	Processor Time	00:00:00.01
	Elapsed Time	00:00:00.00

Between-Subjects Factors

		N
GENOTYPE	KI	30
	WT	26
SEX	F	27
	M	29

Tests of Between-Subjects Effects

Dependent Variable: HindpawStrideLen (mm)

Source	Type III Sum of Squares	df	Mean Square	F	Sig.
Corrected Model	181.084[a]	3	60.361	2.207	.098
Intercept	211971.182	1	211971.182	7749.294	<.001
GENOTYPE	152.475	1	152.475	5.574	.022
SEX	8.348	1	8.348	.305	.583
GENOTYPE * SEX	27.423	1	27.423	1.003	.321
Error	1422.388	52	27.354		
Total	217335.402	56			
Corrected Total	1603.472	55			

a. R Squared = .113 (Adjusted R Squared = .062)

```
UNIANOVA HindpawAvPrintAreaBY GENOTYPE SEX
/METHOD=SSTYPE(3)
/INTERCEPT=INCLUDE
/CRITERIA=ALPHA(.05)
/DESIGN=GENOTYPE SEX GENOTYPE*SEX.
```

Univariate Analysis of Variance

Notes

Output Created		22-AUG-2022 14:55:...
Comments		
Input	Data	/Users/jeffneul/Jeff's Research/Neul Manuscripts/FoxG1/June 2022 stat sex and effect size/SPSS files/SPSS Data Files/June 2022 Gait sex EF data.sav
	Active Dataset	DataSet4
	Filter	<none>
	Weight	<none>
	Split File	<none>
	N of Rows in Working Data File	56
Missing Value Handling	Definition of Missing	User-defined missing values are treated as missing.
	Cases Used	Statistics are based on all cases with valid data for all variables in the model.
Syntax		UNIANOVA HindpawAvPrintArea BY GENOTYPE SEX /METHOD=SSTYPE(3) /INTERCEPT=INCLUDE /CRITERIA=ALPHA(.05) /DESIGN=GENOTYPE SEX GENOTYPE*SEX.
Resources	Processor Time	00:00:00.01
	Elapsed Time	00:00:00.00

Between-Subjects Factors

		N
GENOTYPE	KI	30
	WT	26
SEX	F	27
	M	29

Tests of Between-Subjects Effects

Dependent Variable: HindpawAvPrintArea

Source	Type III Sum of Squares	df	Mean Square	F	Sig.
Corrected Model	11364.791 [a]	3	3788.264	1.402	.253
Intercept	2894901.83	1	2894901.83	1071.487	<.001
GENOTYPE	11332.251	1	11332.251	4.194	.046
SEX	31.538	1	31.538	.012	.914
GENOTYPE * SEX	19.792	1	19.792	.007	.932
Error	140491.555	52	2701.761		
Total	3071417.03	56			
Corrected Total	151856.346	55			

a. R Squared = .075 (Adjusted R Squared = .021)

```
UNIANOVA FrTkWdmm BY GENOTYPE SEX
/METHOD=SSTYPE(3)
/INTERCEPT=INCLUDE
/CRITERIA=ALPHA(.05)
/DESIGN=GENOTYPE SEX GENOTYPE*SEX.
```

Univariate Analysis of Variance

Notes

Output Created		22-AUG-2022 14:55:...
Comments		
Input	Data	/Users/jeffneul/Jeff's Research/Neul Manuscripts/FoxG1/June 2022 stat sex and effect size/SPSS files/SPSS Data Files/June 2022 Gait sex EF data.sav
	Active Dataset	DataSet4
	Filter	<none>
	Weight	<none>
	Split File	<none>
	N of Rows in Working Data File	56
Missing Value Handling	Definition of Missing	User-defined missing values are treated as missing.
	Cases Used	Statistics are based on all cases with valid data for all variables in the model.
Syntax		UNIANOVA FrTkWdmm BY GENOTYPE SEX /METHOD=SSTYPE(3) /INTERCEPT=INCLUDE /CRITERIA=ALPHA(.05) /DESIGN=GENOTYPE SEX GENOTYPE*SEX.
Resources	Processor Time	00:00:00.01
	Elapsed Time	00:00:00.00

Between-Subjects Factors

		N
GENOTYPE	KI	30
	WT	26
SEX	F	27
	M	29

Tests of Between-Subjects Effects

Dependent Variable: FrTkWd (mm)

Source	Type III Sum of Squares	df	Mean Square	F	Sig.
Corrected Model	1.272[a]	3	.424	.457	.713
Intercept	7796.063	1	7796.063	8407.829	<.001
GENOTYPE	.041	1	.041	.044	.835
SEX	1.053	1	1.053	1.135	.292
GENOTYPE * SEX	.154	1	.154	.166	.685
Error	48.216	52	.927		
Total	7987.913	56			
Corrected Total	49.488	55			

a. R Squared = .026 (Adjusted R Squared = -.031)

```
UNIANOVA RrTkWdmm BY GENOTYPE SEX
/METHOD=SSTYPE(3)
/INTERCEPT=INCLUDE
/CRITERIA=ALPHA(.05)
/DESIGN=GENOTYPE SEX GENOTYPE*SEX.
```

Univariate Analysis of Variance

Notes

Output Created		22-AUG-2022 14:56:...
Comments		
Input	Data	/Users/jeffneul/Jeff's Research/Neul Manuscripts/FoxG1/June 2022 stat sex and effect size/SPSS files/SPSS Data Files/June 2022 Gait sex EF data.sav
	Active Dataset	DataSet4
	Filter	<none>
	Weight	<none>
	Split File	<none>
	N of Rows in Working Data File	56
Missing Value Handling	Definition of Missing	User-defined missing values are treated as missing.
	Cases Used	Statistics are based on all cases with valid data for all variables in the model.
Syntax		UNIANOVA RrTkWdmm BY GENOTYPE SEX /METHOD=SSTYPE(3) /INTERCEPT=INCLUDE /CRITERIA=ALPHA(.05) /DESIGN=GENOTYPE SEX GENOTYPE*SEX.
Resources	Processor Time	00:00:00.01
	Elapsed Time	00:00:00.00

Between-Subjects Factors

		N
GENOTYPE	KI	30
	WT	26
SEX	F	27
	M	29

Tests of Between-Subjects Effects

Dependent Variable: RrTkWd (mm)

Source	Type III Sum of Squares	df	Mean Square	F	Sig.
Corrected Model	14.817[a]	3	4.939	2.935	.042
Intercept	23374.497	1	23374.497	13889.489	<.001
GENOTYPE	7.276	1	7.276	4.323	.043
SEX	7.606	1	7.606	4.519	.038
GENOTYPE * SEX	1.592	1	1.592	.946	.335
Error	87.510	52	1.683		
Total	23797.790	56			
Corrected Total	102.327	55			

a. R Squared = .145 (Adjusted R Squared = .095)

```
UNIANOVA OverallAvgRunSpeedmmsBY GENOTYPE SEX
/METHOD=SSTYPE(3)
/INTERCEPT=INCLUDE
/CRITERIA=ALPHA(.05)
/DESIGN=GENOTYPE SEX GENOTYPE*SEX.
```

Univariate Analysis of Variance

Notes

Output Created		22-AUG-2022 14:56:...
Comments		
Input	Data	/Users/jeffneul/Jeff's Research/Neul Manuscripts/FoxG1/June 2022 stat sex and effect size/SPSS files/SPSS Data Files/June 2022 Gait sex EF data.sav
	Active Dataset	DataSet4
	Filter	<none>
	Weight	<none>
	Split File	<none>
	N of Rows in Working Data File	56
Missing Value Handling	Definition of Missing	User-defined missing values are treated as missing.
	Cases Used	Statistics are based on all cases with valid data for all variables in the model.
Syntax		UNIANOVA OverallAvgRunSpeedmms BY GENOTYPE SEX /METHOD=SSTYPE(3) /INTERCEPT=INCLUDE /CRITERIA=ALPHA(.05) /DESIGN=GENOTYPE SEX GENOTYPE*SEX.
Resources	Processor Time	00:00:00.01
	Elapsed Time	00:00:00.00

Between-Subjects Factors

		N
GENOTYPE	KI	30
	WT	26
SEX	F	27
	M	29

Tests of Between-Subjects Effects

Dependent Variable: Overall Avg Run Speed (mm/s):

Source	Type III Sum of Squares	df	Mean Square	F	Sig.
Corrected Model	581.380[a]	3	193.793	.530	.663
Intercept	1813166.52	1	1813166.52	4962.516	<.001
GENOTYPE	432.628	1	432.628	1.184	.282
SEX	128.013	1	128.013	.350	.556
GENOTYPE * SEX	66.461	1	66.461	.182	.672
Error	18999.367	52	365.372		
Total	1868529.23	56			
Corrected Total	19580.747	55			

a. R Squared = .030 (Adjusted R Squared = -.026)

```
UNIANOVA ForepawHomolatCplBY GENOTYPE SEX
/METHOD=SSTYPE(3)
/INTERCEPT=INCLUDE
/CRITERIA=ALPHA(.05)
/DESIGN=GENOTYPE SEX GENOTYPE*SEX.
```

Univariate Analysis of Variance

Notes

Output Created		22-AUG-2022 14:56:...
Comments		
Input	Data	/Users/jeffneul/Jeff's Research/Neul Manuscripts/FoxG1/June 2022 stat sex and effect size/SPSS files/SPSS Data Files/June 2022 Gait sex EF data.sav
	Active Dataset	DataSet4
	Filter	<none>
	Weight	<none>
	Split File	<none>
	N of Rows in Working Data File	56
Missing Value Handling	Definition of Missing	User-defined missing values are treated as missing.
	Cases Used	Statistics are based on all cases with valid data for all variables in the model.
Syntax		UNIANOVA ForepawHomolatCpl BY GENOTYPE SEX /METHOD=SSTYPE(3) /INTERCEPT=INCLUDE /CRITERIA=ALPHA(.05) /DESIGN=GENOTYPE SEX GENOTYPE*SEX.
Resources	Processor Time	00:00:00.01
	Elapsed Time	00:00:00.00

Between-Subjects Factors

		N
GENOTYPE	KI	30
	WT	26
SEX	F	27
	M	29

Tests of Between-Subjects Effects

Dependent Variable: ForepawHomolatCpl

Source	Type III Sum of Squares	df	Mean Square	F	Sig.
Corrected Model	.006[a]	3	.002	1.612	.198
Intercept	15.735	1	15.735	13353.892	<.001
GENOTYPE	.004	1	.004	3.518	.066
SEX	.002	1	.002	1.502	.226
GENOTYPE * SEX	.000	1	.000	.272	.604
Error	.061	52	.001		
Total	16.106	56			
Corrected Total	.067	55			

a. R Squared = .085 (Adjusted R Squared = .032)

```
UNIANOVA ForepawHomologCplBY GENOTYPE SEX
/METHOD=SSTYPE(3)
/INTERCEPT=INCLUDE
/CRITERIA=ALPHA(.05)
/DESIGN=GENOTYPE SEX GENOTYPE*SEX.
```

Univariate Analysis of Variance

Notes

Output Created		22-AUG-2022 14:57:...
Comments		
Input	Data	/Users/jeffneul/Jeff's Research/Neul Manuscripts/FoxG1/June 2022 stat sex and effect size/SPSS files/SPSS Data Files/June 2022 Gait sex EF data.sav
	Active Dataset	DataSet4
	Filter	<none>
	Weight	<none>
	Split File	<none>
	N of Rows in Working Data File	56
Missing Value Handling	Definition of Missing	User-defined missing values are treated as missing.
	Cases Used	Statistics are based on all cases with valid data for all variables in the model.
Syntax		UNIANOVA ForepawHomologCpl BY GENOTYPE SEX /METHOD=SSTYPE(3) /INTERCEPT=INCLUDE /CRITERIA=ALPHA(.05) /DESIGN=GENOTYPE SEX GENOTYPE*SEX.
Resources	Processor Time	00:00:00.01
	Elapsed Time	00:00:00.00

Between-Subjects Factors

		N
GENOTYPE	KI	30
	WT	26
SEX	F	27
	M	29

Tests of Between-Subjects Effects

Dependent Variable: ForepawHomologCpl

Source	Type III Sum of Squares	df	Mean Square	F	Sig.
Corrected Model	.000[a]	3	8.407E-5	1.088	.362
Intercept	13.500	1	13.500	174730.403	<.001
GENOTYPE	3.616E-5	1	3.616E-5	.468	.497
SEX	.000	1	.000	2.292	.136
GENOTYPE * SEX	1.137E-5	1	1.137E-5	.147	.703
Error	.004	52	7.726E-5		
Total	13.721	56			
Corrected Total	.004	55			

a. R Squared = .059 (Adjusted R Squared = .005)

```
UNIANOVA ForepawDiagonalCplBY GENOTYPE SEX
/METHOD=SSTYPE(3)
/INTERCEPT=INCLUDE
/CRITERIA=ALPHA(.05)
/DESIGN=GENOTYPE SEX GENOTYPE*SEX.
```

Univariate Analysis of Variance

Notes

Output Created		22-AUG-2022 14:57:...
Comments		
Input	Data	/Users/jeffneul/Jeff's Research/Neul Manuscripts/FoxG1/June 2022 stat sex and effect size/SPSS files/SPSS Data Files/June 2022 Gait sex EF data.sav
	Active Dataset	DataSet4
	Filter	<none>
	Weight	<none>
	Split File	<none>
	N of Rows in Working Data File	56
Missing Value Handling	Definition of Missing	User-defined missing values are treated as missing.
	Cases Used	Statistics are based on all cases with valid data for all variables in the model.
Syntax		UNIANOVA ForepawDiagonalCpl BY GENOTYPE SEX /METHOD=SSTYPE(3) /INTERCEPT=INCLUDE /CRITERIA=ALPHA(.05) /DESIGN=GENOTYPE SEX GENOTYPE*SEX.
Resources	Processor Time	00:00:00.01
	Elapsed Time	00:00:00.00

Between-Subjects Factors

		N
GENOTYPE	KI	30
	WT	26
SEX	F	27
	M	29

Tests of Between-Subjects Effects

Dependent Variable: ForepawDiagonalCpl

Source	Type III Sum of Squares	df	Mean Square	F	Sig.
Corrected Model	.002[a]	3	.001	.671	.574
Intercept	.234	1	.234	222.835	<.001
GENOTYPE	.002	1	.002	1.561	.217
SEX	.001	1	.001	.597	.443
GENOTYPE * SEX	4.217E-5	1	4.217E-5	.040	.842
Error	.055	52	.001		
Total	.297	56			
Corrected Total	.057	55			

a. R Squared = .037 (Adjusted R Squared = -.018)

```
UNIANOVA HindpawHomolatCplBY GENOTYPE SEX
/METHOD=SSTYPE(3)
/INTERCEPT=INCLUDE
/CRITERIA=ALPHA(.05)
/DESIGN=GENOTYPE SEX GENOTYPE*SEX.
```

Univariate Analysis of Variance

Notes

Output Created		22-AUG-2022 14:57:...
Comments		
Input	Data	/Users/jeffneul/Jeff's Research/Neul Manuscripts/FoxG1/June 2022 stat sex and effect size/SPSS files/SPSS Data Files/June 2022 Gait sex EF data.sav
	Active Dataset	DataSet4
	Filter	<none>
	Weight	<none>
	Split File	<none>
	N of Rows in Working Data File	56
Missing Value Handling	Definition of Missing	User-defined missing values are treated as missing.
	Cases Used	Statistics are based on all cases with valid data for all variables in the model.
Syntax		UNIANOVA HindpawHomolatCpl BY GENOTYPE SEX /METHOD=SSTYPE(3) /INTERCEPT=INCLUDE /CRITERIA=ALPHA(.05) /DESIGN=GENOTYPE SEX GENOTYPE*SEX.
Resources	Processor Time	00:00:00.01
	Elapsed Time	00:00:00.00

Between-Subjects Factors

		N
GENOTYPE	KI	30
	WT	26
SEX	F	27
	M	29

Tests of Between-Subjects Effects

Dependent Variable: HindpawHomolatCpl

Source	Type III Sum of Squares	df	Mean Square	F	Sig.
Corrected Model	.009[a]	3	.003	2.295	.089
Intercept	10.962	1	10.962	7959.130	<.001
GENOTYPE	.009	1	.009	6.803	.012
SEX	.000	1	.000	.216	.644
GENOTYPE * SEX	3.743E-5	1	3.743E-5	.027	.870
Error	.072	52	.001		
Total	11.181	56			
Corrected Total	.081	55			

a. R Squared = .117 (Adjusted R Squared = .066)

```
UNIANOVA HindpawHomologCplBY GENOTYPE SEX
/METHOD=SSTYPE(3)
/INTERCEPT=INCLUDE
/CRITERIA=ALPHA(.05)
/DESIGN=GENOTYPE SEX GENOTYPE*SEX.
```

Univariate Analysis of Variance

Notes

Output Created		22-AUG-2022 14:58:...
Comments		
Input	Data	/Users/jeffneul/Jeff's Research/Neul Manuscripts/FoxG1/June 2022 stat sex and effect size/SPSS files/SPSS Data Files/June 2022 Gait sex EF data.sav
	Active Dataset	DataSet4
	Filter	<none>
	Weight	<none>
	Split File	<none>
	N of Rows in Working Data File	56
Missing Value Handling	Definition of Missing	User-defined missing values are treated as missing.
	Cases Used	Statistics are based on all cases with valid data for all variables in the model.
Syntax		UNIANOVA HindpawHomologCpl BY GENOTYPE SEX /METHOD=SSTYPE(3) /INTERCEPT=INCLUDE /CRITERIA=ALPHA(.05) /DESIGN=GENOTYPE SEX GENOTYPE*SEX.
Resources	Processor Time	00:00:00.01
	Elapsed Time	00:00:00.00

Between-Subjects Factors

		N
GENOTYPE	KI	30
	WT	26
SEX	F	27
	M	29

Tests of Between-Subjects Effects

Dependent Variable: HindpawHomologCpl

Source	Type III Sum of Squares	df	Mean Square	F	Sig.
Corrected Model	.002[a]	3	.001	1.912	.139
Intercept	13.026	1	13.026	49222.401	<.001
GENOTYPE	.001	1	.001	5.380	.024
SEX	5.734E-5	1	5.734E-5	.217	.644
GENOTYPE * SEX	8.998E-5	1	8.998E-5	.340	.562
Error	.014	52	.000		
Total	13.250	56			
Corrected Total	.015	55			

a. R Squared = .099 (Adjusted R Squared = .047)

```
UNIANOVA HindpawDiagonalCplBY GENOTYPE SEX
/METHOD=SSTYPE(3)
/INTERCEPT=INCLUDE
/CRITERIA=ALPHA(.05)
/DESIGN=GENOTYPE SEX GENOTYPE*SEX.
```

Univariate Analysis of Variance

Notes

Output Created		22-AUG-2022 14:58:...
Comments		
Input	Data	/Users/jeffneul/Jeff's Research/Neul Manuscripts/FoxG1/June 2022 stat sex and effect size/SPSS files/SPSS Data Files/June 2022 Gait sex EF data.sav
	Active Dataset	DataSet4
	Filter	<none>
	Weight	<none>
	Split File	<none>
	N of Rows in Working Data File	56
Missing Value Handling	Definition of Missing	User-defined missing values are treated as missing.
	Cases Used	Statistics are based on all cases with valid data for all variables in the model.
Syntax		UNIANOVA HindpawDiagonalCpl BY GENOTYPE SEX /METHOD=SSTYPE(3) /INTERCEPT=INCLUDE /CRITERIA=ALPHA(.05) /DESIGN=GENOTYPE SEX GENOTYPE*SEX.
Resources	Processor Time	00:00:00.01
	Elapsed Time	00:00:00.00

Between-Subjects Factors

		N
GENOTYPE	KI	30
	WT	26
SEX	F	27
	M	29

Tests of Between-Subjects Effects

Dependent Variable: HindpawDiagonalCpl

Source	Type III Sum of Squares	df	Mean Square	F	Sig.
Corrected Model	.003[a]	3	.001	.608	.613
Intercept	.031	1	.031	22.781	<.001
GENOTYPE	.001	1	.001	.515	.476
SEX	7.100E-5	1	7.100E-5	.052	.821
GENOTYPE * SEX	.002	1	.002	1.132	.292
Error	.072	52	.001		
Total	.108	56			
Corrected Total	.074	55			

a. R Squared = .034 (Adjusted R Squared = -.022)

```
UNIANOVA ForepawGaitAngle BY GENOTYPE SEX
/METHOD=SSTYPE(3)
/INTERCEPT=INCLUDE
/CRITERIA=ALPHA(.05)
/DESIGN=GENOTYPE SEX GENOTYPE*SEX.
```

Univariate Analysis of Variance

Notes

Output Created		22-AUG-2022 14:58:...
Comments		
Input	Data	/Users/jeffneul/Jeff's Research/Neul Manuscripts/FoxG1/June 2022 stat sex and effect size/SPSS files/SPSS Data Files/June 2022 Gait sex EF data.sav
	Active Dataset	DataSet4
	Filter	<none>
	Weight	<none>
	Split File	<none>
	N of Rows in Working Data File	56
Missing Value Handling	Definition of Missing	User-defined missing values are treated as missing.
	Cases Used	Statistics are based on all cases with valid data for all variables in the model.
Syntax		UNIANOVA ForepawGaitAngle BY GENOTYPE SEX /METHOD=SSTYPE(3) /INTERCEPT=INCLUDE /CRITERIA=ALPHA(.05) /DESIGN=GENOTYPE SEX GENOTYPE*SEX.
Resources	Processor Time	00:00:00.01
	Elapsed Time	00:00:00.00

Between-Subjects Factors

		N
GENOTYPE	KI	30
	WT	26
SEX	F	27
	M	29

Tests of Between-Subjects Effects

Dependent Variable: ForepawGaitAngle

Source	Type III Sum of Squares	df	Mean Square	F	Sig.
Corrected Model	97.727[a]	3	32.576	.986	.407
Intercept	150746.109	1	150746.109	4561.306	<.001
GENOTYPE	5.887	1	5.887	.178	.675
SEX	19.297	1	19.297	.584	.448
GENOTYPE * SEX	78.158	1	78.158	2.365	.130
Error	1718.543	52	33.049		
Total	155853.653	56			
Corrected Total	1816.269	55			

a. R Squared = .054 (Adjusted R Squared = -.001)

```
UNIANOVA HindpawGaitAngle BY GENOTYPE SEX
/METHOD=SSTYPE(3)
/INTERCEPT=INCLUDE
/CRITERIA=ALPHA(.05)
/DESIGN=GENOTYPE SEX GENOTYPE*SEX.
```

Univariate Analysis of Variance

Notes

Output Created		22-AUG-2022 14:58:...
Comments		
Input	Data	/Users/jeffneul/Jeff's Research/Neul Manuscripts/FoxG1/June 2022 stat sex and effect size/SPSS files/SPSS Data Files/June 2022 Gait sex EF data.sav
	Active Dataset	DataSet4
	Filter	<none>
	Weight	<none>
	Split File	<none>
	N of Rows in Working Data File	56
Missing Value Handling	Definition of Missing	User-defined missing values are treated as missing.
	Cases Used	Statistics are based on all cases with valid data for all variables in the model.
Syntax		UNIANOVA HindpawGaitAngle BY GENOTYPE SEX /METHOD=SSTYPE(3) /INTERCEPT=INCLUDE /CRITERIA=ALPHA(.05) /DESIGN=GENOTYPE SEX GENOTYPE*SEX.
Resources	Processor Time	00:00:00.01
	Elapsed Time	00:00:00.00

Between-Subjects Factors

		N
GENOTYPE	KI	30
	WT	26
SEX	F	27
	M	29

Tests of Between-Subjects Effects

Dependent Variable: HindpawGaitAngle

Source	Type III Sum of Squares	df	Mean Square	F	Sig.
Corrected Model	37.753[a]	3	12.584	.296	.828
Intercept	226237.050	1	226237.050	5318.746	<.001
GENOTYPE	15.749	1	15.749	.370	.546
SEX	12.023	1	12.023	.283	.597
GENOTYPE * SEX	13.387	1	13.387	.315	.577
Error	2211.861	52	42.536		
Total	231749.603	56			
Corrected Total	2249.615	55			

a. R Squared = .017 (Adjusted R Squared = -.040)

```
UNIANOVA BdyRotdeg BY GENOTYPE SEX
/METHOD=SSTYPE(3)
/INTERCEPT=INCLUDE
/CRITERIA=ALPHA(.05)
/DESIGN=GENOTYPE SEX GENOTYPE*SEX.
```

Univariate Analysis of Variance

Notes

Output Created		22-AUG-2022 14:59:...
Comments		
Input	Data	/Users/jeffneul/Jeff's Research/Neul Manuscripts/FoxG1/June 2022 stat sex and effect size/SPSS files/SPSS Data Files/June 2022 Gait sex EF data.sav
	Active Dataset	DataSet4
	Filter	<none>
	Weight	<none>
	Split File	<none>
	N of Rows in Working Data File	56
Missing Value Handling	Definition of Missing	User-defined missing values are treated as missing.
	Cases Used	Statistics are based on all cases with valid data for all variables in the model.
Syntax		UNIANOVA BdyRotdeg BY GENOTYPE SEX /METHOD=SSTYPE(3) /INTERCEPT=INCLUDE /CRITERIA=ALPHA(.05) /DESIGN=GENOTYPE SEX GENOTYPE*SEX.
Resources	Processor Time	00:00:00.01
	Elapsed Time	00:00:00.00

Between-Subjects Factors

		N
GENOTYPE	KI	30
	WT	26
SEX	F	27
	M	29

Tests of Between-Subjects Effects

Dependent Variable: BdyRot(deg)

Source	Type III Sum of Squares	df	Mean Square	F	Sig.
Corrected Model	70.842[a]	3	23.614	3.726	.017
Intercept	151.435	1	151.435	23.892	<.001
GENOTYPE	67.057	1	67.057	10.580	.002
SEX	.207	1	.207	.033	.857
GENOTYPE * SEX	4.418	1	4.418	.697	.408
Error	329.596	52	6.338		
Total	563.129	56			
Corrected Total	400.438	55			

a. R Squared = .177 (Adjusted R Squared = .129)

```
UNIANOVA LongitdPosmm BY GENOTYPE SEX
/METHOD=SSTYPE(3)
/INTERCEPT=INCLUDE
/CRITERIA=ALPHA(.05)
/DESIGN=GENOTYPE SEX GENOTYPE*SEX.
```

Univariate Analysis of Variance

Notes

Output Created		22-AUG-2022 14:59:...
Comments		
Input	Data	/Users/jeffneul/Jeff's Research/Neul Manuscripts/FoxG1/June 2022 stat sex and effect size/SPSS files/SPSS Data Files/June 2022 Gait sex EF data.sav
	Active Dataset	DataSet4
	Filter	<none>
	Weight	<none>
	Split File	<none>
	N of Rows in Working Data File	56
Missing Value Handling	Definition of Missing	User-defined missing values are treated as missing.
	Cases Used	Statistics are based on all cases with valid data for all variables in the model.
Syntax		UNIANOVA LongitdPosmm BY GENOTYPE SEX /METHOD=SSTYPE(3) /INTERCEPT=INCLUDE /CRITERIA=ALPHA(.05) /DESIGN=GENOTYPE SEX GENOTYPE*SEX.
Resources	Processor Time	00:00:00.01
	Elapsed Time	00:00:00.00

Between-Subjects Factors

		N
GENOTYPE	KI	30
	WT	26
SEX	F	27
	M	29

Tests of Between-Subjects Effects

Dependent Variable: Longitd Pos(mm)

Source	Type III Sum of Squares	df	Mean Square	F	Sig.
Corrected Model	491.782[a]	3	163.927	.587	.626
Intercept	438615.947	1	438615.947	1570.882	<.001
GENOTYPE	124.790	1	124.790	.447	.507
SEX	171.791	1	171.791	.615	.436
GENOTYPE * SEX	192.843	1	192.843	.691	.410
Error	14519.253	52	279.216		
Total	460688.138	56			
Corrected Total	15011.036	55			

a. R Squared = .033 (Adjusted R Squared = -.023)

```
UNIANOVA LateralPosmm BY GENOTYPE SEX
/METHOD=SSTYPE(3)
/INTERCEPT=INCLUDE
/CRITERIA=ALPHA(.05)
/DESIGN=GENOTYPE SEX GENOTYPE*SEX.
```

Univariate Analysis of Variance

Notes

Output Created		22-AUG-2022 14:59:...
Comments		
Input	Data	/Users/jeffneul/Jeff's Research/Neul Manuscripts/FoxG1/June 2022 stat sex and effect size/SPSS files/SPSS Data Files/June 2022 Gait sex EF data.sav
	Active Dataset	DataSet4
	Filter	<none>
	Weight	<none>
	Split File	<none>
	N of Rows in Working Data File	56
Missing Value Handling	Definition of Missing	User-defined missing values are treated as missing.
	Cases Used	Statistics are based on all cases with valid data for all variables in the model.
Syntax		UNIANOVA LateralPosmm BY GENOTYPE SEX /METHOD=SSTYPE(3) /INTERCEPT=INCLUDE /CRITERIA=ALPHA(.05) /DESIGN=GENOTYPE SEX GENOTYPE*SEX.
Resources	Processor Time	00:00:00.01
	Elapsed Time	00:00:00.00

Between-Subjects Factors

		N
GENOTYPE	KI	30
	WT	26
SEX	F	27
	M	29

Tests of Between-Subjects Effects

Dependent Variable: Lateral Pos(mm)

Source	Type III Sum of Squares	df	Mean Square	F	Sig.
Corrected Model	1.339[a]	3	.446	.094	.963
Intercept	375670.385	1	375670.385	78882.673	<.001
GENOTYPE	.581	1	.581	.122	.728
SEX	.702	1	.702	.147	.703
GENOTYPE * SEX	.206	1	.206	.043	.836
Error	247.644	52	4.762		
Total	382131.935	56			
Corrected Total	248.983	55			

a. R Squared = .005 (Adjusted R Squared = -.052)

```
UNIANOVA ForepawStridems BY GENOTYPE SEX
/METHOD=SSTYPE(3)
/INTERCEPT=INCLUDE
/EMMEANS=TABLES(GENOTYPE)
/EMMEANS=TABLES(SEX)
/PRINT ETASQ
/CRITERIA=ALPHA(.05)
/DESIGN=GENOTYPE SEX.
```

Univariate Analysis of Variance

Notes

Output Created		22-AUG-2022 15:00:...
Comments		
Input	Data	/Users/jeffneul/Jeff's Research/Neul Manuscripts/FoxG1/June 2022 stat sex and effect size/SPSS files/SPSS Data Files/June 2022 Gait sex EF data.sav
	Active Dataset	DataSet4
	Filter	<none>
	Weight	<none>
	Split File	<none>
	N of Rows in Working Data File	56
Missing Value Handling	Definition of Missing	User-defined missing values are treated as missing.
	Cases Used	Statistics are based on all cases with valid data for all variables in the model.
Syntax		UNIANOVA ForepawStridems BY GENOTYPE SEX /METHOD=SSTYPE(3) /INTERCEPT=INCLUDE /EMMEANS=TABLES (GENOTYPE) /EMMEANS=TABLES (SEX) /PRINT ETASQ /CRITERIA=ALPHA(.05) /DESIGN=GENOTYPE SEX.
Resources	Processor Time	00:00:00.01
	Elapsed Time	00:00:00.00

Between-Subjects Factors

		N
GENOTYPE	KI	30
	WT	26
SEX	F	27
	M	29

Tests of Between-Subjects Effects

Dependent Variable: ForepawStride (ms)

Source	Type III Sum of Squares	df	Mean Square	F	Sig.	Partial Eta Squared
Corrected Model	3130.361[a]	2	1565.180	1.836	.169	.065
Intercept	4180790.32	1	4180790.32	4903.717	<.001	.989
GENOTYPE	2421.313	1	2421.313	2.840	.098	.051
SEX	1001.347	1	1001.347	1.174	.283	.022
Error	45186.519	53	852.576			
Total	4244051.19	56				
Corrected Total	48316.879	55				

a. R Squared = .065 (Adjusted R Squared = .029)

Estimated Marginal Means

1. GENOTYPE

Dependent Variable: ForepawStride (ms)

GENOTYPE	Mean	Std. Error	95% Confidence Interval Lower Bound	95% Confidence Interval Upper Bound
KI	267.415	5.357	256.671	278.159
WT	280.673	5.734	269.171	292.174

2. SEX

Dependent Variable: ForepawStride (ms)

SEX	Mean	Std. Error	95% Confidence Interval Lower Bound	95% Confidence Interval Upper Bound
F	269.789	5.621	258.514	281.064
M	278.299	5.464	267.338	289.259

```
UNIANOVA ForepawPercofSwingBY GENOTYPE SEX
/METHOD=SSTYPE(3)
/INTERCEPT=INCLUDE
/EMMEANS=TABLES(GENOTYPE)
/EMMEANS=TABLES(SEX)
/PRINT ETASQ
/CRITERIA=ALPHA(.05)
/DESIGN=GENOTYPE SEX.
```

Univariate Analysis of Variance

Notes

Output Created		22-AUG-2022 15:00:...
Comments		
Input	Data	/Users/jeffneul/Jeff's Research/Neul Manuscripts/FoxG1/June 2022 stat sex and effect size/SPSS files/SPSS Data Files/June 2022 Gait sex EF data.sav
	Active Dataset	DataSet4
	Filter	<none>
	Weight	<none>
	Split File	<none>
	N of Rows in Working Data File	56
Missing Value Handling	Definition of Missing	User-defined missing values are treated as missing.
	Cases Used	Statistics are based on all cases with valid data for all variables in the model.
Syntax		UNIANOVA ForepawPercofSwing BY GENOTYPE SEX<br>/METHOD=SSTYPE(3)<br>/INTERCEPT=INCLUDE<br>/EMMEANS=TABLES (GENOTYPE)<br>/EMMEANS=TABLES (SEX)<br>/PRINT ETASQ<br>/CRITERIA=ALPHA(.05)<br>/DESIGN=GENOTYPE SEX.
Resources	Processor Time	00:00:00.02
	Elapsed Time	00:00:00.00

Between-Subjects Factors

		N
GENOTYPE	KI	30
	WT	26
SEX	F	27
	M	29

Tests of Between-Subjects Effects

Dependent Variable: ForepawPercofSwing

Source	Type III Sum of Squares	df	Mean Square	F	Sig.
Corrected Model	.008[a]	2	.004	3.762	.030
Intercept	14.220	1	14.220	12878.060	<.001
GENOTYPE	.008	1	.008	7.183	.010
SEX	.001	1	.001	.743	.393
Error	.059	53	.001		
Total	14.412	56			
Corrected Total	.067	55			

Tests of Between-Subjects Effects

Dependent Variable: ForepawPercofSwing

Source	Partial Eta Squared
Corrected Model	.124
Intercept	.996
GENOTYPE	.119
SEX	.014
Error	
Total	
Corrected Total	

a. R Squared = .124 (Adjusted R Squared = .091)

Estimated Marginal Means

1. GENOTYPE

Dependent Variable: ForepawPercofSwing

GENOTYPE	Mean	Std. Error	95% Confidence Interval Lower Bound	95% Confidence Interval Upper Bound
KI	.517	.006	.505	.530
WT	.493	.007	.480	.506

2. SEX

Dependent Variable: ForepawPercofSwing

SEX	Mean	Std. Error	95% Confidence Interval Lower Bound	Upper Bound
F	.509	.006	.496	.522
M	.502	.006	.489	.514

```
UNIANOVA ForepawStrideLenmmBY GENOTYPE SEX
/METHOD=SSTYPE(3)
/INTERCEPT=INCLUDE
/EMMEANS=TABLES(GENOTYPE)
/EMMEANS=TABLES(SEX)
/PRINT ETASQ
/CRITERIA=ALPHA(.05)
/DESIGN=GENOTYPE SEX.
```

Univariate Analysis of Variance

Notes

Output Created		22-AUG-2022 15:01:...
Comments		
Input	Data	/Users/jeffneul/Jeff's Research/Neul Manuscripts/FoxG1/June 2022 stat sex and effect size/SPSS files/SPSS Data Files/June 2022 Gait sex EF data.sav
	Active Dataset	DataSet4
	Filter	<none>
	Weight	<none>
	Split File	<none>
	N of Rows in Working Data File	56
Missing Value Handling	Definition of Missing	User-defined missing values are treated as missing.
	Cases Used	Statistics are based on all cases with valid data for all variables in the model.

Notes

Syntax		UNIANOVA ForepawStrideLenmm BY GENOTYPE SEX<br>/METHOD=SSTYPE(3)<br>/INTERCEPT=INCLUDE<br>/EMMEANS=TABLES (GENOTYPE)<br>/EMMEANS=TABLES (SEX)<br>/PRINT ETASQ<br>/CRITERIA=ALPHA(.05)<br>/DESIGN=GENOTYPE SEX.
Resources	Processor Time	00:00:00.02
	Elapsed Time	00:00:00.00

Between-Subjects Factors

		N
GENOTYPE	KI	30
	WT	26
SEX	F	27
	M	29

Tests of Between-Subjects Effects

Dependent Variable: ForepawStrideLen (mm)

Source	Type III Sum of Squares	df	Mean Square	F	Sig.
Corrected Model	55.541[a]	2	27.771	2.145	.127
Intercept	196768.115	1	196768.115	15199.000	<.001
GENOTYPE	24.110	1	24.110	1.862	.178
SEX	25.605	1	25.605	1.978	.165
Error	686.145	53	12.946		
Total	199154.050	56			
Corrected Total	741.686	55			

Tests of Between-Subjects Effects

Dependent Variable: ForepawStrideLen (mm)

Source	Partial Eta Squared
Corrected Model	.075
Intercept	.997
GENOTYPE	.034
SEX	.036
Error	
Total	
Corrected Total	

a. R Squared = .075 (Adjusted R Squared = .040)

Estimated Marginal Means

1. GENOTYPE

Dependent Variable: ForepawStrideLen (mm)

GENOTYPE	Mean	Std. Error	95% Confidence Interval	
			Lower Bound	Upper Bound
KI	60.114	.660	58.790	61.438
WT	58.791	.707	57.373	60.208

2. SEX

Dependent Variable: ForepawStrideLen (mm)

SEX	Mean	Std. Error	95% Confidence Interval	
			Lower Bound	Upper Bound
F	58.772	.693	57.382	60.161
M	60.133	.673	58.782	61.483

```
UNIANOVA ForepawAvPrintAreaBY GENOTYPE SEX
/METHOD=SSTYPE(3)
/INTERCEPT=INCLUDE
/EMMEANS=TABLES(GENOTYPE)
/EMMEANS=TABLES(SEX)
/PRINT ETASQ
/CRITERIA=ALPHA(.05)
/DESIGN=GENOTYPE SEX.
```

Univariate Analysis of Variance

Notes

Output Created		22-AUG-2022 15:01:...
Comments		
Input	Data	/Users/jeffneul/Jeff's Research/Neul Manuscripts/FoxG1/June 2022 stat sex and effect size/SPSS files/SPSS Data Files/June 2022 Gait sex EF data.sav
	Active Dataset	DataSet4
	Filter	<none>
	Weight	<none>
	Split File	<none>
	N of Rows in Working Data File	56
Missing Value Handling	Definition of Missing	User-defined missing values are treated as missing.
	Cases Used	Statistics are based on all cases with valid data for all variables in the model.
Syntax		UNIANOVA ForepawAvPrintArea BY GENOTYPE SEX<br>/METHOD=SSTYPE(3)<br>/INTERCEPT=INCLUDE<br>/EMMEANS=TABLES (GENOTYPE)<br>/EMMEANS=TABLES (SEX)<br>/PRINT ETASQ<br>/CRITERIA=ALPHA(.05)<br>/DESIGN=GENOTYPE SEX.
Resources	Processor Time	00:00:00.02
	Elapsed Time	00:00:00.00

Between-Subjects Factors

		N
GENOTYPE	KI	30
	WT	26
SEX	F	27
	M	29

Tests of Between-Subjects Effects

Dependent Variable: ForepawAvPrintArea

Source	Type III Sum of Squares	df	Mean Square	F	Sig.	Partial Eta Squared
Corrected Model	8709.415 [a]	2	4354.707	5.601	.006	.174
Intercept	1143510.18	1	1143510.18	1470.814	<.001	.965
GENOTYPE	5168.032	1	5168.032	6.647	.013	.111
SEX	4452.105	1	4452.105	5.726	.020	.098
Error	41205.794	53	777.468			
Total	1194257.43	56				
Corrected Total	49915.208	55				

a. R Squared = .174 (Adjusted R Squared = .143)

Estimated Marginal Means

1. GENOTYPE

Dependent Variable: ForepawAvPrintArea

GENOTYPE	Mean	Std. Error	95% Confidence Interval Lower Bound	95% Confidence Interval Upper Bound
KI	133.637	5.115	123.377	143.896
WT	153.006	5.476	142.023	163.989

2. SEX

Dependent Variable: ForepawAvPrintArea

SEX	Mean	Std. Error	95% Confidence Interval Lower Bound	95% Confidence Interval Upper Bound
F	134.350	5.368	123.583	145.116
M	152.293	5.218	141.827	162.759

```
UNIANOVA HindpawStridems BY GENOTYPE SEX
/METHOD=SSTYPE(3)
/INTERCEPT=INCLUDE
/EMMEANS=TABLES(GENOTYPE)
/EMMEANS=TABLES(SEX)
/PRINT ETASQ
/CRITERIA=ALPHA(.05)
/DESIGN=GENOTYPE SEX.
```

Univariate Analysis of Variance

Notes

Output Created		22-AUG-2022 15:01:...
Comments		
Input	Data	/Users/jeffneul/Jeff's Research/Neul Manuscripts/FoxG1/June 2022 stat sex and effect size/SPSS files/SPSS Data Files/June 2022 Gait sex EF data.sav
	Active Dataset	DataSet4
	Filter	<none>
	Weight	<none>
	Split File	<none>
	N of Rows in Working Data File	56
Missing Value Handling	Definition of Missing	User-defined missing values are treated as missing.
	Cases Used	Statistics are based on all cases with valid data for all variables in the model.
Syntax		UNIANOVA HindpawStridems BY GENOTYPE SEX<br>/METHOD=SSTYPE(3)<br>/INTERCEPT=INCLUDE<br>/EMMEANS=TABLES (GENOTYPE)<br>/EMMEANS=TABLES (SEX)<br>/PRINT ETASQ<br>/CRITERIA=ALPHA(.05)<br>/DESIGN=GENOTYPE SEX.
Resources	Processor Time	00:00:00.01
	Elapsed Time	00:00:00.00

Between-Subjects Factors

		N
GENOTYPE	KI	30
	WT	26
SEX	F	27
	M	29

Tests of Between-Subjects Effects

Dependent Variable: HindpawStride (ms)

Source	Type III Sum of Squares	df	Mean Square	F	Sig.	Partial Eta Squared
Corrected Model	297.315[a]	2	148.658	.168	.845	.006
Intercept	4472282.35	1	4472282.35	5065.008	<.001	.990
GENOTYPE	27.172	1	27.172	.031	.861	.001
SEX	249.589	1	249.589	.283	.597	.005
Error	46797.750	53	882.976			
Total	4541914.50	56				
Corrected Total	47095.066	55				

a. R Squared = .006 (Adjusted R Squared = -.031)

Estimated Marginal Means

1. GENOTYPE

Dependent Variable: HindpawStride (ms)

GENOTYPE	Mean	Std. Error	95% Confidence Interval	
			Lower Bound	Upper Bound
KI	282.734	5.451	271.800	293.668
WT	284.138	5.836	272.434	295.843

2. SEX

Dependent Variable: HindpawStride (ms)

SEX	Mean	Std. Error	95% Confidence Interval	
			Lower Bound	Upper Bound
F	285.560	5.721	274.086	297.034
M	281.312	5.561	270.158	292.466

```
UNIANOVA HindpawPercofSwingBY GENOTYPE SEX
/METHOD=SSTYPE(3)
/INTERCEPT=INCLUDE
/EMMEANS=TABLES(GENOTYPE)
/EMMEANS=TABLES(SEX)
/PRINT ETASQ
/CRITERIA=ALPHA(.05)
/DESIGN=GENOTYPE SEX.
```

Univariate Analysis of Variance

Notes

Output Created		22-AUG-2022 15:01:...
Comments		
Input	Data	/Users/jeffneul/Jeff's Research/Neul Manuscripts/FoxG1/June 2022 stat sex and effect size/SPSS files/SPSS Data Files/June 2022 Gait sex EF data.sav
	Active Dataset	DataSet4
	Filter	<none>
	Weight	<none>
	Split File	<none>
	N of Rows in Working Data File	56
Missing Value Handling	Definition of Missing	User-defined missing values are treated as missing.
	Cases Used	Statistics are based on all cases with valid data for all variables in the model.
Syntax		UNIANOVA HindpawPercofSwing BY GENOTYPE SEX<br>/METHOD=SSTYPE(3)<br>/INTERCEPT=INCLUDE<br>/EMMEANS=TABLES (GENOTYPE)<br>/EMMEANS=TABLES (SEX)<br>/PRINT ETASQ<br>/CRITERIA=ALPHA(.05)<br>/DESIGN=GENOTYPE SEX.
Resources	Processor Time	00:00:00.02
	Elapsed Time	00:00:00.00

Between-Subjects Factors

		N
GENOTYPE	KI	30
	WT	26
SEX	F	27
	M	29

Tests of Between-Subjects Effects

Dependent Variable: HindpawPercofSwing

Source	Type III Sum of Squares	df	Mean Square	F	Sig.	Partial Eta Squared
Corrected Model	.024[a]	2	.012	6.426	.003	.195
Intercept	16.789	1	16.789	8879.548	<.001	.994
GENOTYPE	.022	1	.022	11.770	.001	.182
SEX	.004	1	.004	1.943	.169	.035
Error	.100	53	.002			
Total	17.084	56				
Corrected Total	.125	55				

a. R Squared = .195 (Adjusted R Squared = .165)

Estimated Marginal Means

1. GENOTYPE

Dependent Variable: HindpawPercofSwing

GENOTYPE	Mean	Std. Error	95% Confidence Interval	
			Lower Bound	Upper Bound
KI	.569	.008	.553	.585
WT	.529	.009	.512	.546

2. SEX

Dependent Variable: HindpawPercofSwing

SEX	Mean	Std. Error	95% Confidence Interval	
			Lower Bound	Upper Bound
F	.557	.008	.541	.574
M	.541	.008	.525	.557

```
UNIANOVA HindpawStrideLenmmBY GENOTYPE SEX
/METHOD=SSTYPE(3)
/INTERCEPT=INCLUDE
/EMMEANS=TABLES(GENOTYPE)
/EMMEANS=TABLES(SEX)
/PRINT ETASQ
/CRITERIA=ALPHA(.05)
/DESIGN=GENOTYPE SEX.
```

Univariate Analysis of Variance

Notes

Output Created		22-AUG-2022 15:02:...
Comments		
Input	Data	/Users/jeffneul/Jeff's Research/Neul Manuscripts/FoxG1/June 2022 stat sex and effect size/SPSS files/SPSS Data Files/June 2022 Gait sex EF data.sav
	Active Dataset	DataSet4
	Filter	<none>
	Weight	<none>
	Split File	<none>
	N of Rows in Working Data File	56
Missing Value Handling	Definition of Missing	User-defined missing values are treated as missing.
	Cases Used	Statistics are based on all cases with valid data for all variables in the model.
Syntax		UNIANOVA HindpawStrideLenmm BY GENOTYPE SEX /METHOD=SSTYPE(3) /INTERCEPT=INCLUDE /EMMEANS=TABLES (GENOTYPE) /EMMEANS=TABLES (SEX) /PRINT ETASQ /CRITERIA=ALPHA(.05) /DESIGN=GENOTYPE SEX.
Resources	Processor Time	00:00:00.02
	Elapsed Time	00:00:00.00

Between-Subjects Factors

		N
GENOTYPE	KI	30
	WT	26
SEX	F	27
	M	29

Tests of Between-Subjects Effects

Dependent Variable: HindpawStrideLen (mm)

Source	Type III Sum of Squares	df	Mean Square	F	Sig.	Partial Eta Squared
Corrected Model	153.661[a]	2	76.831	2.809	.069	.096
Intercept	213753.601	1	213753.601	7814.084	<.001	.993
GENOTYPE	149.798	1	149.798	5.476	.023	.094
SEX	10.491	1	10.491	.384	.538	.007
Error	1449.810	53	27.355			
Total	217335.402	56				
Corrected Total	1603.472	55				

a. R Squared = .096 (Adjusted R Squared = .062)

Estimated Marginal Means

1. GENOTYPE

Dependent Variable: HindpawStrideLen (mm)

GENOTYPE	Mean	Std. Error	95% Confidence Interval Lower Bound	Upper Bound
KI	63.614	.959	61.689	65.538
WT	60.316	1.027	58.256	62.377

2. SEX

Dependent Variable: HindpawStrideLen (mm)

SEX	Mean	Std. Error	95% Confidence Interval Lower Bound	Upper Bound
F	62.401	1.007	60.381	64.420
M	61.530	.979	59.566	63.493

```
UNIANOVA HindpawAvPrintAreaBY GENOTYPE SEX
/METHOD=SSTYPE(3)
/INTERCEPT=INCLUDE
/EMMEANS=TABLES(GENOTYPE)
/EMMEANS=TABLES(SEX)
/PRINT ETASQ
/CRITERIA=ALPHA(.05)
/DESIGN=GENOTYPE SEX.
```

Univariate Analysis of Variance

Notes

Output Created		22-AUG-2022 15:02:...
Comments		
Input	Data	/Users/jeffneul/Jeff's Research/Neul Manuscripts/FoxG1/June 2022 stat sex and effect size/SPSS files/SPSS Data Files/June 2022 Gait sex EF data.sav
	Active Dataset	DataSet4
	Filter	<none>
	Weight	<none>
	Split File	<none>
	N of Rows in Working Data File	56
Missing Value Handling	Definition of Missing	User-defined missing values are treated as missing.
	Cases Used	Statistics are based on all cases with valid data for all variables in the model.
Syntax		UNIANOVA HindpawAvPrintArea BY GENOTYPE SEX<br>/METHOD=SSTYPE(3)<br>/INTERCEPT=INCLUDE<br>/EMMEANS=TABLES (GENOTYPE)<br>/EMMEANS=TABLES (SEX)<br>/PRINT ETASQ<br>/CRITERIA=ALPHA(.05)<br>/DESIGN=GENOTYPE SEX.
Resources	Processor Time	00:00:00.02
	Elapsed Time	00:00:00.00

Between-Subjects Factors

		N
GENOTYPE	KI	30
	WT	26
SEX	F	27
	M	29

Tests of Between-Subjects Effects

Dependent Variable: HindpawAvPrintArea

Source	Type III Sum of Squares	df	Mean Square	F	Sig.	Partial Eta Squared
Corrected Model	11344.999 [a]	2	5672.499	2.140	.128	.075
Intercept	2927695.87	1	2927695.87	1104.309	<.001	.954
GENOTYPE	11317.208	1	11317.208	4.269	.044	.075
SEX	35.044	1	35.044	.013	.909	.000
Error	140511.347	53	2651.157			
Total	3071417.03	56				
Corrected Total	151856.346	55				

a. R Squared = .075 (Adjusted R Squared = .040)

Estimated Marginal Means

1. GENOTYPE

Dependent Variable: HindpawAvPrintArea

GENOTYPE	Mean	Std. Error	95% Confidence Interval	
			Lower Bound	Upper Bound
KI	214.995	9.446	196.049	233.941
WT	243.658	10.112	223.376	263.940

2. SEX

Dependent Variable: HindpawAvPrintArea

SEX	Mean	Std. Error	95% Confidence Interval	
			Lower Bound	Upper Bound
F	228.530	9.912	208.648	248.412
M	230.122	9.636	210.795	249.449

```
UNIANOVA FrTkWdmm BY GENOTYPE SEX
/METHOD=SSTYPE(3)
/INTERCEPT=INCLUDE
/EMMEANS=TABLES(GENOTYPE)
/EMMEANS=TABLES(SEX)
/PRINT ETASQ
/CRITERIA=ALPHA(.05)
/DESIGN=GENOTYPE SEX.
```

Univariate Analysis of Variance

Notes

Output Created		22-AUG-2022 15:02:...
Comments		
Input	Data	/Users/jeffneul/Jeff's Research/Neul Manuscripts/FoxG1/June 2022 stat sex and effect size/SPSS files/SPSS Data Files/June 2022 Gait sex EF data.sav
	Active Dataset	DataSet4
	Filter	<none>
	Weight	<none>
	Split File	<none>
	N of Rows in Working Data File	56
Missing Value Handling	Definition of Missing	User-defined missing values are treated as missing.
	Cases Used	Statistics are based on all cases with valid data for all variables in the model.
Syntax		UNIANOVA FrTkWdmm BY GENOTYPE SEX<br>/METHOD=SSTYPE(3)<br>/INTERCEPT=INCLUDE<br>/EMMEANS=TABLES (GENOTYPE)<br>/EMMEANS=TABLES (SEX)<br>/PRINT ETASQ<br>/CRITERIA=ALPHA(.05)<br>/DESIGN=GENOTYPE SEX.
Resources	Processor Time	00:00:00.02
	Elapsed Time	00:00:00.00

Between-Subjects Factors

		N
GENOTYPE	KI	30
	WT	26
SEX	F	27
	M	29

Tests of Between-Subjects Effects

Dependent Variable: FrTkWd (mm)

Source	Type III Sum of Squares	df	Mean Square	F	Sig.	Partial Eta Squared
Corrected Model	1.118[a]	2	.559	.612	.546	.023
Intercept	7887.363	1	7887.363	8642.236	<.001	.994
GENOTYPE	.038	1	.038	.041	.840	.001
SEX	1.111	1	1.111	1.217	.275	.022
Error	48.371	53	.913			
Total	7987.913	56				
Corrected Total	49.488	55				

a. R Squared = .023 (Adjusted R Squared = -.014)

Estimated Marginal Means

1. GENOTYPE

Dependent Variable: FrTkWd (mm)

GENOTYPE	Mean	Std. Error	95% Confidence Interval	
			Lower Bound	Upper Bound
KI	11.877	.175	11.525	12.228
WT	11.929	.188	11.553	12.305

2. SEX

Dependent Variable: FrTkWd (mm)

SEX	Mean	Std. Error	95% Confidence Interval	
			Lower Bound	Upper Bound
F	11.761	.184	11.392	12.130
M	12.045	.179	11.686	12.403

```
UNIANOVA RrTkWdmm BY GENOTYPE SEX
/METHOD=SSTYPE(3)
/INTERCEPT=INCLUDE
/EMMEANS=TABLES(GENOTYPE)
/EMMEANS=TABLES(SEX)
/PRINT ETASQ
/CRITERIA=ALPHA(.05)
/DESIGN=GENOTYPE SEX.
```

Univariate Analysis of Variance

Notes

Output Created		22-AUG-2022 15:02:...
Comments		
Input	Data	/Users/jeffneul/Jeff's Research/Neul Manuscripts/FoxG1/June 2022 stat sex and effect size/SPSS files/SPSS Data Files/June 2022 Gait sex EF data.sav
	Active Dataset	DataSet4
	Filter	<none>
	Weight	<none>
	Split File	<none>
	N of Rows in Working Data File	56
Missing Value Handling	Definition of Missing	User-defined missing values are treated as missing.
	Cases Used	Statistics are based on all cases with valid data for all variables in the model.
Syntax		UNIANOVA RrTkWdmm BY GENOTYPE SEX<br>/METHOD=SSTYPE(3)<br>/INTERCEPT=INCLUDE<br>/EMMEANS=TABLES (GENOTYPE)<br>/EMMEANS=TABLES (SEX)<br>/PRINT ETASQ<br>/CRITERIA=ALPHA(.05)<br>/DESIGN=GENOTYPE SEX.
Resources	Processor Time	00:00:00.01
	Elapsed Time	00:00:00.00

Between-Subjects Factors

		N
GENOTYPE	KI	30
	WT	26
SEX	F	27
	M	29

Tests of Between-Subjects Effects

Dependent Variable: RrTkWd (mm)

Source	Type III Sum of Squares	df	Mean Square	F	Sig.
Corrected Model	13.225[a]	2	6.613	3.933	.026
Intercept	23586.268	1	23586.268	14029.658	<.001
GENOTYPE	7.425	1	7.425	4.417	.040
SEX	7.188	1	7.188	4.275	.044
Error	89.102	53	1.681		
Total	23797.790	56			
Corrected Total	102.327	55			

Tests of Between-Subjects Effects

Dependent Variable: RrTkWd (mm)

Source	Partial Eta Squared
Corrected Model	.129
Intercept	.996
GENOTYPE	.077
SEX	.075
Error	
Total	
Corrected Total	

a. R Squared = .129 (Adjusted R Squared = .096)

Estimated Marginal Means

1. GENOTYPE

Dependent Variable: RrTkWd (mm)

GENOTYPE	Mean	Std. Error	95% Confidence Interval Lower Bound	95% Confidence Interval Upper Bound
KI	20.216	.238	19.739	20.694
WT	20.951	.255	20.440	21.461

2. SEX

Dependent Variable: RrTkWd (mm)

SEX	Mean	Std. Error	95% Confidence Interval	
			Lower Bound	Upper Bound
F	20.223	.250	19.722	20.724
M	20.944	.243	20.457	21.431

```
UNIANOVA OverallAvgRunSpeedmmsBY GENOTYPE SEX
/METHOD=SSTYPE(3)
/INTERCEPT=INCLUDE
/EMMEANS=TABLES(GENOTYPE)
/EMMEANS=TABLES(SEX)
/PRINT ETASQ
/CRITERIA=ALPHA(.05)
/DESIGN=GENOTYPE SEX.
```

Univariate Analysis of Variance

Notes

Output Created		22-AUG-2022 15:02:...
Comments		
Input	Data	/Users/jeffneul/Jeff's Research/Neul Manuscripts/FoxG1/June 2022 stat sex and effect size/SPSS files/SPSS Data Files/June 2022 Gait sex EF data.sav
	Active Dataset	DataSet4
	Filter	<none>
	Weight	<none>
	Split File	<none>
	N of Rows in Working Data File	56
Missing Value Handling	Definition of Missing	User-defined missing values are treated as missing.
	Cases Used	Statistics are based on all cases with valid data for all variables in the model.

Notes

Syntax		UNIANOVA OverallAvgRunSpeedmms BY GENOTYPE SEX<br>/METHOD=SSTYPE(3)<br>/INTERCEPT=INCLUDE<br>/EMMEANS=TABLES (GENOTYPE)<br>/EMMEANS=TABLES (SEX)<br>/PRINT ETASQ<br>/CRITERIA=ALPHA(.05)<br>/DESIGN=GENOTYPE SEX.
Resources	Processor Time	00:00:00.02
	Elapsed Time	00:00:00.00

Between-Subjects Factors

		N
GENOTYPE	KI	30
	WT	26
SEX	F	27
	M	29

Tests of Between-Subjects Effects

Dependent Variable: Overall Avg Run Speed (mm/s):

Source	Type III Sum of Squares	df	Mean Square	F	Sig.	Partial Eta Squared
Corrected Model	514.919[a]	2	257.460	.716	.494	.026
Intercept	1835009.15	1	1835009.15	5101.037	<.001	.990
GENOTYPE	440.090	1	440.090	1.223	.274	.023
SEX	116.723	1	116.723	.324	.571	.006
Error	19065.828	53	359.733			
Total	1868529.23	56				
Corrected Total	19580.747	55				

a. R Squared = .026 (Adjusted R Squared = -.010)

Estimated Marginal Means

1. GENOTYPE

Dependent Variable: Overall Avg Run Speed (mm/s):

GENOTYPE	Mean	Std. Error	95% Confidence Interval	
			Lower Bound	Upper Bound
KI	184.382	3.479	177.403	191.361
WT	178.730	3.725	171.258	186.201

2. SEX

Dependent Variable: Overall Avg Run Speed (mm/s):

SEX	Mean	Std. Error	95% Confidence Interval	
			Lower Bound	Upper Bound
F	183.008	3.651	175.685	190.332
M	180.103	3.549	172.984	187.222

```
UNIANOVA ForepawHomolatCplBY GENOTYPE SEX
/METHOD=SSTYPE(3)
/INTERCEPT=INCLUDE
/EMMEANS=TABLES(GENOTYPE)
/EMMEANS=TABLES(SEX)
/PRINT ETASQ
/CRITERIA=ALPHA(.05)
/DESIGN=GENOTYPE SEX.
```

Univariate Analysis of Variance

Notes

Output Created		22-AUG-2022 15:03:...
Comments		
Input	Data	/Users/jeffneul/Jeff's Research/Neul Manuscripts/FoxG1/June 2022 stat sex and effect size/SPSS files/SPSS Data Files/June 2022 Gait sex EF data.sav
	Active Dataset	DataSet4
	Filter	<none>
	Weight	<none>
	Split File	<none>
	N of Rows in Working Data File	56
Missing Value Handling	Definition of Missing	User-defined missing values are treated as missing.
	Cases Used	Statistics are based on all cases with valid data for all variables in the model.
Syntax		UNIANOVA ForepawHomolatCpl BY GENOTYPE SEX<br>/METHOD=SSTYPE(3)<br>/INTERCEPT=INCLUDE<br>/EMMEANS=TABLES (GENOTYPE)<br>/EMMEANS=TABLES (SEX)<br>/PRINT ETASQ<br>/CRITERIA=ALPHA(.05)<br>/DESIGN=GENOTYPE SEX.
Resources	Processor Time	00:00:00.01
	Elapsed Time	00:00:00.00

Between-Subjects Factors

		N
GENOTYPE	KI	30
	WT	26
SEX	F	27
	M	29

Tests of Between-Subjects Effects

Dependent Variable: ForepawHomolatCpl

Source	Type III Sum of Squares	df	Mean Square	F	Sig.
Corrected Model	.005[a]	2	.003	2.314	.109
Intercept	15.919	1	15.919	13698.520	<.001
GENOTYPE	.004	1	.004	3.611	.063
SEX	.002	1	.002	1.446	.235
Error	.062	53	.001		
Total	16.106	56			
Corrected Total	.067	55			

Tests of Between-Subjects Effects

Dependent Variable: ForepawHomolatCpl

Source	Partial Eta Squared
Corrected Model	.080
Intercept	.996
GENOTYPE	.064
SEX	.027
Error	
Total	
Corrected Total	

a. R Squared = .080 (Adjusted R Squared = .046)

Estimated Marginal Means

1. GENOTYPE

Dependent Variable: ForepawHomolatCpl

GENOTYPE	Mean	Std. Error	95% Confidence Interval Lower Bound	95% Confidence Interval Upper Bound
KI	.543	.006	.531	.556
WT	.526	.007	.513	.539

2. SEX

Dependent Variable: ForepawHomolatCpl

SEX	Mean	Std. Error	95% Confidence Interval Lower Bound	Upper Bound
F	.540	.007	.527	.553
M	.529	.006	.516	.542

```
UNIANOVA ForepawHomologCplBY GENOTYPE SEX
/METHOD=SSTYPE(3)
/INTERCEPT=INCLUDE
/EMMEANS=TABLES(GENOTYPE)
/EMMEANS=TABLES(SEX)
/PRINT ETASQ
/CRITERIA=ALPHA(.05)
/DESIGN=GENOTYPE SEX.
```

Univariate Analysis of Variance

Notes

Output Created		22-AUG-2022 15:03:...
Comments		
Input	Data	/Users/jeffneul/Jeff's Research/Neul Manuscripts/FoxG1/June 2022 stat sex and effect size/SPSS files/SPSS Data Files/June 2022 Gait sex EF data.sav
	Active Dataset	DataSet4
	Filter	<none>
	Weight	<none>
	Split File	<none>
	N of Rows in Working Data File	56
Missing Value Handling	Definition of Missing	User-defined missing values are treated as missing.
	Cases Used	Statistics are based on all cases with valid data for all variables in the model.

Notes

Syntax		UNIANOVA ForepawHomologCpl BY GENOTYPE SEX<br>/METHOD=SSTYPE(3)<br>/INTERCEPT=INCLUDE<br>/EMMEANS=TABLES (GENOTYPE)<br>/EMMEANS=TABLES (SEX)<br>/PRINT ETASQ<br>/CRITERIA=ALPHA(.05)<br>/DESIGN=GENOTYPE SEX.
Resources	Processor Time	00:00:00.02
	Elapsed Time	00:00:00.00

Between-Subjects Factors

		N
GENOTYPE	KI	30
	WT	26
SEX	F	27
	M	29

Tests of Between-Subjects Effects

Dependent Variable: ForepawHomologCpl

Source	Type III Sum of Squares	df	Mean Square	F	Sig.
Corrected Model	.000[a]	2	.000	1.584	.215
Intercept	13.643	1	13.643	179468.999	<.001
GENOTYPE	3.705E-5	1	3.705E-5	.487	.488
SEX	.000	1	.000	2.418	.126
Error	.004	53	7.602E-5		
Total	13.721	56			
Corrected Total	.004	55			

Tests of Between-Subjects Effects

Dependent Variable: ForepawHomologCpl

Source	Partial Eta Squared
Corrected Model	.056
Intercept	1.000
GENOTYPE	.009
SEX	.044
Error	
Total	
Corrected Total	

a. R Squared = .056 (Adjusted R Squared = .021)

Estimated Marginal Means

1. GENOTYPE

Dependent Variable: ForepawHomologCpl

GENOTYPE	Mean	Std. Error	95% Confidence Interval	
			Lower Bound	Upper Bound
KI	.494	.002	.491	.497
WT	.496	.002	.492	.499

2. SEX

Dependent Variable: ForepawHomologCpl

SEX	Mean	Std. Error	95% Confidence Interval	
			Lower Bound	Upper Bound
F	.497	.002	.493	.500
M	.493	.002	.490	.496

```
UNIANOVA ForepawDiagonalCplBY GENOTYPE SEX
/METHOD=SSTYPE(3)
/INTERCEPT=INCLUDE
/EMMEANS=TABLES(GENOTYPE)
/EMMEANS=TABLES(SEX)
/PRINT ETASQ
/CRITERIA=ALPHA(.05)
/DESIGN=GENOTYPE SEX.
```

Univariate Analysis of Variance

Notes

Output Created		22-AUG-2022 15:03:...
Comments		
Input	Data	/Users/jeffneul/Jeff's Research/Neul Manuscripts/FoxG1/June 2022 stat sex and effect size/SPSS files/SPSS Data Files/June 2022 Gait sex EF data.sav
	Active Dataset	DataSet4
	Filter	<none>
	Weight	<none>
	Split File	<none>
	N of Rows in Working Data File	56
Missing Value Handling	Definition of Missing	User-defined missing values are treated as missing.
	Cases Used	Statistics are based on all cases with valid data for all variables in the model.
Syntax		UNIANOVA ForepawDiagonalCpl BY GENOTYPE SEX /METHOD=SSTYPE(3) /INTERCEPT=INCLUDE /EMMEANS=TABLES (GENOTYPE) /EMMEANS=TABLES (SEX) /PRINT ETASQ /CRITERIA=ALPHA(.05) /DESIGN=GENOTYPE SEX.
Resources	Processor Time	00:00:00.02
	Elapsed Time	00:00:00.00

Between-Subjects Factors

		N
GENOTYPE	KI	30
	WT	26
SEX	F	27
	M	29

Tests of Between-Subjects Effects

Dependent Variable: ForepawDiagonalCpl

Source	Type III Sum of Squares	df	Mean Square	F	Sig.	Partial Eta Squared
Corrected Model	.002[a]	2	.001	1.005	.373	.037
Intercept	.237	1	.237	230.029	<.001	.813
GENOTYPE	.002	1	.002	1.601	.211	.029
SEX	.001	1	.001	.590	.446	.011
Error	.055	53	.001			
Total	.297	56				
Corrected Total	.057	55				

a. R Squared = .037 (Adjusted R Squared = .000)

Estimated Marginal Means

1. GENOTYPE

Dependent Variable: ForepawDiagonalCpl

GENOTYPE	Mean	Std. Error	95% Confidence Interval Lower Bound	95% Confidence Interval Upper Bound
KI	.071	.006	.059	.083
WT	.060	.006	.047	.072

2. SEX

Dependent Variable: ForepawDiagonalCpl

SEX	Mean	Std. Error	95% Confidence Interval Lower Bound	95% Confidence Interval Upper Bound
F	.069	.006	.056	.081
M	.062	.006	.050	.074

```
UNIANOVA HindpawHomolatCplBY GENOTYPE SEX
/METHOD=SSTYPE(3)
/INTERCEPT=INCLUDE
/EMMEANS=TABLES(GENOTYPE)
/EMMEANS=TABLES(SEX)
/PRINT ETASQ
/CRITERIA=ALPHA(.05)
/DESIGN=GENOTYPE SEX.
```

Univariate Analysis of Variance

Notes

Output Created		22-AUG-2022 15:03:...
Comments		
Input	Data	/Users/jeffneul/Jeff's Research/Neul Manuscripts/FoxG1/June 2022 stat sex and effect size/SPSS files/SPSS Data Files/June 2022 Gait sex EF data.sav
	Active Dataset	DataSet4
	Filter	<none>
	Weight	<none>
	Split File	<none>
	N of Rows in Working Data File	56
Missing Value Handling	Definition of Missing	User-defined missing values are treated as missing.
	Cases Used	Statistics are based on all cases with valid data for all variables in the model.
Syntax		UNIANOVA HindpawHomolatCpl BY GENOTYPE SEX /METHOD=SSTYPE(3) /INTERCEPT=INCLUDE /EMMEANS=TABLES (GENOTYPE) /EMMEANS=TABLES (SEX) /PRINT ETASQ /CRITERIA=ALPHA(.05) /DESIGN=GENOTYPE SEX.
Resources	Processor Time	00:00:00.01
	Elapsed Time	00:00:00.00

Between-Subjects Factors

		N
GENOTYPE	KI	30
	WT	26
SEX	F	27
	M	29

Tests of Between-Subjects Effects

Dependent Variable: HindpawHomolatCpl

Source	Type III Sum of Squares	df	Mean Square	F	Sig.	Partial Eta Squared
Corrected Model	.009[a]	2	.005	3.493	.038	.116
Intercept	11.076	1	11.076	8192.246	<.001	.994
GENOTYPE	.009	1	.009	6.952	.011	.116
SEX	.000	1	.000	.211	.648	.004
Error	.072	53	.001			
Total	11.181	56				
Corrected Total	.081	55				

a. R Squared = .116 (Adjusted R Squared = .083)

Estimated Marginal Means

1. GENOTYPE

Dependent Variable: HindpawHomolatCpl

GENOTYPE	Mean	Std. Error	95% Confidence Interval Lower Bound	95% Confidence Interval Upper Bound
KI	.433	.007	.419	.447
WT	.459	.007	.445	.474

2. SEX

Dependent Variable: HindpawHomolatCpl

SEX	Mean	Std. Error	95% Confidence Interval Lower Bound	95% Confidence Interval Upper Bound
F	.444	.007	.430	.458
M	.448	.007	.435	.462

```
UNIANOVA HindpawHomologCplBY GENOTYPE SEX
/METHOD=SSTYPE(3)
/INTERCEPT=INCLUDE
/EMMEANS=TABLES(GENOTYPE)
/EMMEANS=TABLES(SEX)
/PRINT ETASQ
/CRITERIA=ALPHA(.05)
/DESIGN=GENOTYPE SEX.
```

Univariate Analysis of Variance

Notes

Output Created		22-AUG-2022 15:04:...
Comments		
Input	Data	/Users/jeffneul/Jeff's Research/Neul Manuscripts/FoxG1/June 2022 stat sex and effect size/SPSS files/SPSS Data Files/June 2022 Gait sex EF data.sav
	Active Dataset	DataSet4
	Filter	<none>
	Weight	<none>
	Split File	<none>
	N of Rows in Working Data File	56
Missing Value Handling	Definition of Missing	User-defined missing values are treated as missing.
	Cases Used	Statistics are based on all cases with valid data for all variables in the model.
Syntax		UNIANOVA HindpawHomologCpl BY GENOTYPE SEX /METHOD=SSTYPE(3) /INTERCEPT=INCLUDE /EMMEANS=TABLES (GENOTYPE) /EMMEANS=TABLES (SEX) /PRINT ETASQ /CRITERIA=ALPHA(.05) /DESIGN=GENOTYPE SEX.
Resources	Processor Time	00:00:00.02
	Elapsed Time	00:00:00.00

Between-Subjects Factors

		N
GENOTYPE	KI	30
	WT	26
SEX	F	27
	M	29

Tests of Between-Subjects Effects

Dependent Variable: HindpawHomologCpl

Source	Type III Sum of Squares	df	Mean Square	F	Sig.
Corrected Model	.001[a]	2	.001	2.732	.074
Intercept	13.174	1	13.174	50407.863	<.001
GENOTYPE	.001	1	.001	5.392	.024
SEX	6.741E-5	1	6.741E-5	.258	.614
Error	.014	53	.000		
Total	13.250	56			
Corrected Total	.015	55			

Tests of Between-Subjects Effects

Dependent Variable: HindpawHomologCpl

Source	Partial Eta Squared
Corrected Model	.093
Intercept	.999
GENOTYPE	.092
SEX	.005
Error	
Total	
Corrected Total	

a. R Squared = .093 (Adjusted R Squared = .059)

Estimated Marginal Means

1. GENOTYPE

Dependent Variable: HindpawHomologCpl

GENOTYPE	Mean	Std. Error	95% Confidence Interval Lower Bound	95% Confidence Interval Upper Bound
KI	.481	.003	.475	.487
WT	.492	.003	.485	.498

2. SEX

Dependent Variable: HindpawHomologCpl

SEX	Mean	Std. Error	95% Confidence Interval	
			Lower Bound	Upper Bound
F	.485	.003	.479	.492
M	.488	.003	.481	.494

```
UNIANOVA HindpawDiagonalCplBY GENOTYPE SEX
/METHOD=SSTYPE(3)
/INTERCEPT=INCLUDE
/EMMEANS=TABLES(GENOTYPE)
/EMMEANS=TABLES(SEX)
/PRINT ETASQ
/CRITERIA=ALPHA(.05)
/DESIGN=GENOTYPE SEX.
```

Univariate Analysis of Variance

Notes

Output Created		22-AUG-2022 15:04:...
Comments		
Input	Data	/Users/jeffneul/Jeff's Research/Neul Manuscripts/FoxG1/June 2022 stat sex and effect size/SPSS files/SPSS Data Files/June 2022 Gait sex EF data.sav
	Active Dataset	DataSet4
	Filter	<none>
	Weight	<none>
	Split File	<none>
	N of Rows in Working Data File	56
Missing Value Handling	Definition of Missing	User-defined missing values are treated as missing.
	Cases Used	Statistics are based on all cases with valid data for all variables in the model.

Notes

Syntax		UNIANOVA HindpawDiagonalCpl BY GENOTYPE SEX<br>/METHOD=SSTYPE(3)<br>/INTERCEPT=INCLUDE<br>/EMMEANS=TABLES (GENOTYPE)<br>/EMMEANS=TABLES (SEX)<br>/PRINT ETASQ<br>/CRITERIA=ALPHA(.05)<br>/DESIGN=GENOTYPE SEX.
Resources	Processor Time	00:00:00.02
	Elapsed Time	00:00:00.00

Between-Subjects Factors

		N
GENOTYPE	KI	30
	WT	26
SEX	F	27
	M	29

Tests of Between-Subjects Effects

Dependent Variable: HindpawDiagonalCpl

Source	Type III Sum of Squares	df	Mean Square	F	Sig.	Partial Eta Squared
Corrected Model	.001[a]	2	.000	.345	.710	.013
Intercept	.033	1	.033	24.039	<.001	.312
GENOTYPE	.001	1	.001	.547	.463	.010
SEX	.000	1	.000	.088	.768	.002
Error	.073	53	.001			
Total	.108	56				
Corrected Total	.074	55				

a. R Squared = .013 (Adjusted R Squared = -.024)

Estimated Marginal Means

1. GENOTYPE

Dependent Variable: HindpawDiagonalCpl

GENOTYPE	Mean	Std. Error	95% Confidence Interval Lower Bound	95% Confidence Interval Upper Bound
KI	-.028	.007	-.042	-.014
WT	-.021	.007	-.035	-.006

2. SEX

Dependent Variable: HindpawDiagonalCpl

SEX	Mean	Std. Error	95% Confidence Interval Lower Bound	95% Confidence Interval Upper Bound
F	-.023	.007	-.037	-.009
M	-.026	.007	-.040	-.012

```
UNIANOVA ForepawGaitAngle BY GENOTYPE SEX
/METHOD=SSTYPE(3)
/INTERCEPT=INCLUDE
/EMMEANS=TABLES(GENOTYPE)
/EMMEANS=TABLES(SEX)
/PRINT ETASQ
/CRITERIA=ALPHA(.05)
/DESIGN=GENOTYPE SEX.
```

Univariate Analysis of Variance

Notes

Output Created		22-AUG-2022 15:04:...
Comments		
Input	Data	/Users/jeffneul/Jeff's Research/Neul Manuscripts/FoxG1/June 2022 stat sex and effect size/SPSS files/SPSS Data Files/June 2022 Gait sex EF data.sav
	Active Dataset	DataSet4
	Filter	<none>
	Weight	<none>
	Split File	<none>
	N of Rows in Working Data File	56
Missing Value Handling	Definition of Missing	User-defined missing values are treated as missing.
	Cases Used	Statistics are based on all cases with valid data for all variables in the model.
Syntax		UNIANOVA ForepawGaitAngle BY GENOTYPE SEX /METHOD=SSTYPE(3) /INTERCEPT=INCLUDE /EMMEANS=TABLES (GENOTYPE) /EMMEANS=TABLES (SEX) /PRINT ETASQ /CRITERIA=ALPHA(.05) /DESIGN=GENOTYPE SEX.
Resources	Processor Time	00:00:00.02
	Elapsed Time	00:00:00.00

Between-Subjects Factors

		N
GENOTYPE	KI	30
	WT	26
SEX	F	27
	M	29

Tests of Between-Subjects Effects

Dependent Variable: ForepawGaitAngle

Source	Type III Sum of Squares	df	Mean Square	F	Sig.	Partial Eta Squared
Corrected Model	19.569[a]	2	9.784	.289	.750	.011
Intercept	153088.999	1	153088.999	4515.899	<.001	.988
GENOTYPE	6.841	1	6.841	.202	.655	.004
SEX	14.611	1	14.611	.431	.514	.008
Error	1796.700	53	33.900			
Total	155853.653	56				
Corrected Total	1816.269	55				

a. R Squared = .011 (Adjusted R Squared = -.027)

Estimated Marginal Means

1. GENOTYPE

Dependent Variable: ForepawGaitAngle

GENOTYPE	Mean	Std. Error	95% Confidence Interval	
			Lower Bound	Upper Bound
KI	52.792	1.068	50.650	54.935
WT	52.088	1.143	49.794	54.381

2. SEX

Dependent Variable: ForepawGaitAngle

SEX	Mean	Std. Error	95% Confidence Interval	
			Lower Bound	Upper Bound
F	52.954	1.121	50.706	55.202
M	51.926	1.090	49.741	54.112

```
UNIANOVA HindpawGaitAngle BY GENOTYPE SEX
/METHOD=SSTYPE(3)
/INTERCEPT=INCLUDE
/EMMEANS=TABLES(GENOTYPE)
/EMMEANS=TABLES(SEX)
/PRINT ETASQ
/CRITERIA=ALPHA(.05)
/DESIGN=GENOTYPE SEX.
```

Univariate Analysis of Variance

Notes

Output Created		22-AUG-2022 15:04:...
Comments		
Input	Data	/Users/jeffneul/Jeff's Research/Neul Manuscripts/FoxG1/June 2022 stat sex and effect size/SPSS files/SPSS Data Files/June 2022 Gait sex EF data.sav
	Active Dataset	DataSet4
	Filter	<none>
	Weight	<none>
	Split File	<none>
	N of Rows in Working Data File	56
Missing Value Handling	Definition of Missing	User-defined missing values are treated as missing.
	Cases Used	Statistics are based on all cases with valid data for all variables in the model.
Syntax		UNIANOVA HindpawGaitAngle BY GENOTYPE SEX<br>/METHOD=SSTYPE(3)<br>/INTERCEPT=INCLUDE<br>/EMMEANS=TABLES (GENOTYPE)<br>/EMMEANS=TABLES (SEX)<br>/PRINT ETASQ<br>/CRITERIA=ALPHA(.05)<br>/DESIGN=GENOTYPE SEX.
Resources	Processor Time	00:00:00.02
	Elapsed Time	00:00:00.00

Between-Subjects Factors

		N
GENOTYPE	KI	30
	WT	26
SEX	F	27
	M	29

Tests of Between-Subjects Effects

Dependent Variable: HindpawGaitAngle

Source	Type III Sum of Squares	df	Mean Square	F	Sig.	Partial Eta Squared
Corrected Model	24.366[a]	2	12.183	.290	.749	.011
Intercept	228313.165	1	228313.165	5437.864	<.001	.990
GENOTYPE	16.382	1	16.382	.390	.535	.007
SEX	10.464	1	10.464	.249	.620	.005
Error	2225.248	53	41.986			
Total	231749.603	56				
Corrected Total	2249.615	55				

a. R Squared = .011 (Adjusted R Squared = -.026)

Estimated Marginal Means

1. GENOTYPE

Dependent Variable: HindpawGaitAngle

GENOTYPE	Mean	Std. Error	95% Confidence Interval	
			Lower Bound	Upper Bound
KI	63.495	1.189	61.111	65.880
WT	64.586	1.273	62.034	67.138

2. SEX

Dependent Variable: HindpawGaitAngle

SEX	Mean	Std. Error	95% Confidence Interval	
			Lower Bound	Upper Bound
F	63.606	1.247	61.104	66.108
M	64.476	1.213	62.043	66.908

```
UNIANOVA BdyRotdeg BY GENOTYPE SEX
/METHOD=SSTYPE(3)
/INTERCEPT=INCLUDE
/EMMEANS=TABLES(GENOTYPE)
/EMMEANS=TABLES(SEX)
/PRINT ETASQ
/CRITERIA=ALPHA(.05)
/DESIGN=GENOTYPE SEX.
```

Univariate Analysis of Variance

Notes

Output Created		22-AUG-2022 15:04:...
Comments		
Input	Data	/Users/jeffneul/Jeff's Research/Neul Manuscripts/FoxG1/June 2022 stat sex and effect size/SPSS files/SPSS Data Files/June 2022 Gait sex EF data.sav
	Active Dataset	DataSet4
	Filter	<none>
	Weight	<none>
	Split File	<none>
	N of Rows in Working Data File	56
Missing Value Handling	Definition of Missing	User-defined missing values are treated as missing.
	Cases Used	Statistics are based on all cases with valid data for all variables in the model.
Syntax		UNIANOVA BdyRotdeg BY GENOTYPE SEX<br>/METHOD=SSTYPE(3)<br>/INTERCEPT=INCLUDE<br>/EMMEANS=TABLES (GENOTYPE)<br>/EMMEANS=TABLES (SEX)<br>/PRINT ETASQ<br>/CRITERIA=ALPHA(.05)<br>/DESIGN=GENOTYPE SEX.
Resources	Processor Time	00:00:00.02
	Elapsed Time	00:00:00.00

Between-Subjects Factors

		N
GENOTYPE	KI	30
	WT	26
SEX	F	27
	M	29

Tests of Between-Subjects Effects

Dependent Variable: BdyRot(deg)

Source	Type III Sum of Squares	df	Mean Square	F	Sig.	Partial Eta Squared
Corrected Model	66.424 [a]	2	33.212	5.270	.008	.166
Intercept	147.715	1	147.715	23.439	<.001	.307
GENOTYPE	66.355	1	66.355	10.529	.002	.166
SEX	.353	1	.353	.056	.814	.001
Error	334.014	53	6.302			
Total	563.129	56				
Corrected Total	400.438	55				

a. R Squared = .166 (Adjusted R Squared = .134)

Estimated Marginal Means

1. GENOTYPE

Dependent Variable: BdyRot(deg)

GENOTYPE	Mean	Std. Error	95% Confidence Interval	
			Lower Bound	Upper Bound
KI	-2.726	.461	-3.650	-1.803
WT	-.532	.493	-1.520	.457

2. SEX

Dependent Variable: BdyRot(deg)

SEX	Mean	Std. Error	95% Confidence Interval	
			Lower Bound	Upper Bound
F	-1.709	.483	-2.678	-.739
M	-1.549	.470	-2.491	-.607

```
UNIANOVA LongitdPosmm BY GENOTYPE SEX
/METHOD=SSTYPE(3)
/INTERCEPT=INCLUDE
/EMMEANS=TABLES(GENOTYPE)
/EMMEANS=TABLES(SEX)
/PRINT ETASQ
/CRITERIA=ALPHA(.05)
/DESIGN=GENOTYPE SEX.
```

Univariate Analysis of Variance

Notes

Output Created		22-AUG-2022 15:04:...
Comments		
Input	Data	/Users/jeffneul/Jeff's Research/Neul Manuscripts/FoxG1/June 2022 stat sex and effect size/SPSS files/SPSS Data Files/June 2022 Gait sex EF data.sav
	Active Dataset	DataSet4
	Filter	<none>
	Weight	<none>
	Split File	<none>
	N of Rows in Working Data File	56
Missing Value Handling	Definition of Missing	User-defined missing values are treated as missing.
	Cases Used	Statistics are based on all cases with valid data for all variables in the model.
Syntax		UNIANOVA LongitdPosmm BY GENOTYPE SEX<br>/METHOD=SSTYPE(3)<br>/INTERCEPT=INCLUDE<br>/EMMEANS=TABLES (GENOTYPE)<br>/EMMEANS=TABLES (SEX)<br>/PRINT ETASQ<br>/CRITERIA=ALPHA(.05)<br>/DESIGN=GENOTYPE SEX.
Resources	Processor Time	00:00:00.01
	Elapsed Time	00:00:00.00

Between-Subjects Factors

		N
GENOTYPE	KI	30
	WT	26
SEX	F	27
	M	29

Tests of Between-Subjects Effects

Dependent Variable: Longitd Pos(mm)

Source	Type III Sum of Squares	df	Mean Square	F	Sig.	Partial Eta Squared
Corrected Model	298.939[a]	2	149.470	.538	.587	.020
Intercept	441427.023	1	441427.023	1590.231	<.001	.968
GENOTYPE	118.315	1	118.315	.426	.517	.008
SEX	149.430	1	149.430	.538	.466	.010
Error	14712.096	53	277.587			
Total	460688.138	56				
Corrected Total	15011.036	55				

a. R Squared = .020 (Adjusted R Squared = -.017)

Estimated Marginal Means

1. GENOTYPE

Dependent Variable: Longitd Pos(mm)

GENOTYPE	Mean	Std. Error	95% Confidence Interval Lower Bound	Upper Bound
KI	90.513	3.056	84.382	96.643
WT	87.582	3.272	81.019	94.145

2. SEX

Dependent Variable: Longitd Pos(mm)

SEX	Mean	Std. Error	95% Confidence Interval Lower Bound	Upper Bound
F	87.404	3.207	80.970	93.837
M	90.691	3.118	84.437	96.945

```
UNIANOVA LateralPosmm BY GENOTYPE SEX
/METHOD=SSTYPE(3)
/INTERCEPT=INCLUDE
/EMMEANS=TABLES(GENOTYPE)
/EMMEANS=TABLES(SEX)
/PRINT ETASQ
/CRITERIA=ALPHA(.05)
/DESIGN=GENOTYPE SEX.
```

Univariate Analysis of Variance

Notes

Output Created		22-AUG-2022 15:05:...
Comments		
Input	Data	/Users/jeffneul/Jeff's Research/Neul Manuscripts/FoxG1/June 2022 stat sex and effect size/SPSS files/SPSS Data Files/June 2022 Gait sex EF data.sav
	Active Dataset	DataSet4
	Filter	<none>
	Weight	<none>
	Split File	<none>
	N of Rows in Working Data File	56
Missing Value Handling	Definition of Missing	User-defined missing values are treated as missing.
	Cases Used	Statistics are based on all cases with valid data for all variables in the model.
Syntax		UNIANOVA LateralPosmm BY GENOTYPE SEX<br>/METHOD=SSTYPE(3)<br>/INTERCEPT=INCLUDE<br>/EMMEANS=TABLES (GENOTYPE)<br>/EMMEANS=TABLES (SEX)<br>/PRINT ETASQ<br>/CRITERIA=ALPHA(.05)<br>/DESIGN=GENOTYPE SEX.
Resources	Processor Time	00:00:00.02
	Elapsed Time	00:00:00.00

Between-Subjects Factors

		N
GENOTYPE	KI	30
	WT	26
SEX	F	27
	M	29

Tests of Between-Subjects Effects

Dependent Variable: Lateral Pos(mm)

Source	Type III Sum of Squares	df	Mean Square	F	Sig.
Corrected Model	1.133[a]	2	.567	.121	.886
Intercept	379662.921	1	379662.921	81186.734	<.001
GENOTYPE	.596	1	.596	.127	.722
SEX	.656	1	.656	.140	.710
Error	247.850	53	4.676		
Total	382131.935	56			
Corrected Total	248.983	55			

Tests of Between-Subjects Effects

Dependent Variable: Lateral Pos(mm)

Source	Partial Eta Squared
Corrected Model	.005
Intercept	.999
GENOTYPE	.002
SEX	.003
Error	
Total	
Corrected Total	

a. R Squared = .005 (Adjusted R Squared = -.033)

Estimated Marginal Means

1. GENOTYPE

Dependent Variable: Lateral Pos(mm)

GENOTYPE	Mean	Std. Error	95% Confidence Interval Lower Bound	95% Confidence Interval Upper Bound
KI	82.479	.397	81.683	83.275
WT	82.687	.425	81.835	83.539

2. SEX

Dependent Variable: Lateral Pos(mm)

SEX	Mean	Std. Error	95% Confidence Interval	
			Lower Bound	Upper Bound
F	82.474	.416	81.639	83.309
M	82.692	.405	81.880	83.503

Fear Conditioning

```
UNIANOVA Prestim BY GENOTYPE SEX
/METHOD=SSTYPE(3)
/INTERCEPT=INCLUDE
/CRITERIA=ALPHA(.05)
/DESIGN=GENOTYPE SEX GENOTYPE*SEX.
```

Univariate Analysis of Variance

Notes

Output Created		22-AUG-2022 16:01:...
Comments		
Input	Data	/Users/jeffneul/Jeff's Research/Neul Manuscripts/FoxG1/June 2022 stat sex and effect size/SPSS files/SPSS Data Files/June 2022 CF sex EF data.sav
	Active Dataset	DataSet5
	Filter	<none>
	Weight	<none>
	Split File	<none>
	N of Rows in Working Data File	30
Missing Value Handling	Definition of Missing	User-defined missing values are treated as missing.
	Cases Used	Statistics are based on all cases with valid data for all variables in the model.
Syntax		UNIANOVA Prestim BY GENOTYPE SEX /METHOD=SSTYPE(3) /INTERCEPT=INCLUDE /CRITERIA=ALPHA(.05) /DESIGN=GENOTYPE SEX GENOTYPE*SEX.
Resources	Processor Time	00:00:00.01
	Elapsed Time	00:00:00.00

Between-Subjects Factors

		N
GENOTYPE	KI	15
	WT	15
SEX	F	14
	M	16

Tests of Between-Subjects Effects

Dependent Variable: Pre-stim

Source	Type III Sum of Squares	df	Mean Square	F	Sig.
Corrected Model	.093[a]	3	.031	4.811	.009
Intercept	2.695	1	2.695	417.729	<.001
GENOTYPE	.083	1	.083	12.801	.001
SEX	.010	1	.010	1.561	.223
GENOTYPE * SEX	4.409E-6	1	4.409E-6	.001	.979
Error	.168	26	.006		
Total	2.990	30			
Corrected Total	.261	29			

a. R Squared = .357 (Adjusted R Squared = .283)

```
UNIANOVA ContextPrestim BY GENOTYPE SEX
/METHOD=SSTYPE(3)
/INTERCEPT=INCLUDE
/CRITERIA=ALPHA(.05)
/DESIGN=GENOTYPE SEX GENOTYPE*SEX.
```

Univariate Analysis of Variance

Notes

Output Created		22-AUG-2022 16:01:...
Comments		
Input	Data	/Users/jeffneul/Jeff's Research/Neul Manuscripts/FoxG1/June 2022 stat sex and effect size/SPSS files/SPSS Data Files/June 2022 CF sex EF data.sav
	Active Dataset	DataSet5
	Filter	<none>
	Weight	<none>
	Split File	<none>
	N of Rows in Working Data File	30
Missing Value Handling	Definition of Missing	User-defined missing values are treated as missing.
	Cases Used	Statistics are based on all cases with valid data for all variables in the model.
Syntax		UNIANOVA ContextPrestim BY GENOTYPE SEX<br>/METHOD=SSTYPE(3)<br>/INTERCEPT=INCLUDE<br>/CRITERIA=ALPHA(.05)<br>/DESIGN=GENOTYPE SEX GENOTYPE*SEX.
Resources	Processor Time	00:00:00.01
	Elapsed Time	00:00:00.00

Between-Subjects Factors

		N
GENOTYPE	KI	15
	WT	15
SEX	F	14
	M	16

Tests of Between-Subjects Effects

Dependent Variable: Context-Prestim

Source	Type III Sum of Squares	df	Mean Square	F	Sig.
Corrected Model	.383[a]	3	.128	8.295	<.001
Intercept	4.547	1	4.547	295.587	<.001
GENOTYPE	.376	1	.376	24.417	<.001
SEX	.003	1	.003	.195	.663
GENOTYPE * SEX	.001	1	.001	.037	.848
Error	.400	26	.015		
Total	5.335	30			
Corrected Total	.783	29			

a. R Squared = .489 (Adjusted R Squared = .430)

```
UNIANOVA CuePrestim BY GENOTYPE SEX
/METHOD=SSTYPE(3)
/INTERCEPT=INCLUDE
/CRITERIA=ALPHA(.05)
/DESIGN=GENOTYPE SEX GENOTYPE*SEX.
```

Univariate Analysis of Variance

Notes

Output Created		22-AUG-2022 16:01:...
Comments		
Input	Data	/Users/jeffneul/Jeff's Research/Neul Manuscripts/FoxG1/June 2022 stat sex and effect size/SPSS files/SPSS Data Files/June 2022 CF sex EF data.sav
	Active Dataset	DataSet5
	Filter	<none>
	Weight	<none>
	Split File	<none>
	N of Rows in Working Data File	30
Missing Value Handling	Definition of Missing	User-defined missing values are treated as missing.
	Cases Used	Statistics are based on all cases with valid data for all variables in the model.
Syntax		UNIANOVA CuePrestim BY GENOTYPE SEX /METHOD=SSTYPE(3) /INTERCEPT=INCLUDE /CRITERIA=ALPHA(.05) /DESIGN=GENOTYPE SEX GENOTYPE*SEX.
Resources	Processor Time	00:00:00.01
	Elapsed Time	00:00:00.00

Between-Subjects Factors

		N
GENOTYPE	KI	15
	WT	15
SEX	F	14
	M	16

Tests of Between-Subjects Effects

Dependent Variable: Cue-Prestim

Source	Type III Sum of Squares	df	Mean Square	F	Sig.
Corrected Model	.323[a]	3	.108	9.118	<.001
Intercept	7.643	1	7.643	647.674	<.001
GENOTYPE	.300	1	.300	25.389	<.001
SEX	.016	1	.016	1.316	.262
GENOTYPE * SEX	.003	1	.003	.219	.644
Error	.307	26	.012		
Total	8.261	30			
Corrected Total	.630	29			

a. R Squared = .513 (Adjusted R Squared = .456)

```
UNIANOVA Prestim BY GENOTYPE SEX
/METHOD=SSTYPE(3)
/INTERCEPT=INCLUDE
/EMMEANS=TABLES(GENOTYPE)
/EMMEANS=TABLES(SEX)
/PRINT ETASQ
/CRITERIA=ALPHA(.05)
/DESIGN=GENOTYPE SEX.
```

Univariate Analysis of Variance

Notes

Output Created		22-AUG-2022 16:03:...
Comments		
Input	Data	/Users/jeffneul/Jeff's Research/Neul Manuscripts/FoxG1/June 2022 stat sex and effect size/SPSS files/SPSS Data Files/June 2022 CF sex EF data.sav
	Active Dataset	DataSet5
	Filter	<none>
	Weight	<none>
	Split File	<none>
	N of Rows in Working Data File	30
Missing Value Handling	Definition of Missing	User-defined missing values are treated as missing.
	Cases Used	Statistics are based on all cases with valid data for all variables in the model.
Syntax		UNIANOVA Prestim BY GENOTYPE SEX /METHOD=SSTYPE(3) /INTERCEPT=INCLUDE /EMMEANS=TABLES (GENOTYPE) /EMMEANS=TABLES (SEX) /PRINT ETASQ /CRITERIA=ALPHA(.05) /DESIGN=GENOTYPE SEX.
Resources	Processor Time	00:00:00.01
	Elapsed Time	00:00:00.00

Between-Subjects Factors

		N
GENOTYPE	KI	15
	WT	15
SEX	F	14
	M	16

Tests of Between-Subjects Effects

Dependent Variable: Pre-stim

Source	Type III Sum of Squares	df	Mean Square	F	Sig.	Partial Eta Squared
Corrected Model	.093[a]	2	.047	7.493	.003	.357
Intercept	2.695	1	2.695	433.784	<.001	.941
GENOTYPE	.083	1	.083	13.365	.001	.331
SEX	.010	1	.010	1.621	.214	.057
Error	.168	27	.006			
Total	2.990	30				
Corrected Total	.261	29				

a. R Squared = .357 (Adjusted R Squared = .309)

Estimated Marginal Means

1. GENOTYPE

Dependent Variable: Pre-stim

GENOTYPE	Mean	Std. Error	95% Confidence Interval	
			Lower Bound	Upper Bound
KI	.353	.020	.311	.395
WT	.248	.020	.206	.290

2. SEX

Dependent Variable: Pre-stim

SEX	Mean	Std. Error	95% Confidence Interval	
			Lower Bound	Upper Bound
F	.282	.021	.239	.325
M	.319	.020	.278	.359

```
UNIANOVA ContextPrestim BY GENOTYPE SEX
/METHOD=SSTYPE(3)
/INTERCEPT=INCLUDE
/EMMEANS=TABLES(GENOTYPE)
/EMMEANS=TABLES(SEX)
/PRINT ETASQ
/CRITERIA=ALPHA(.05)
/DESIGN=GENOTYPE SEX.
```

Univariate Analysis of Variance

Notes

Output Created		22-AUG-2022 16:04:...
Comments		
Input	Data	/Users/jeffneul/Jeff's Research/Neul Manuscripts/FoxG1/June 2022 stat sex and effect size/SPSS files/SPSS Data Files/June 2022 CF sex EF data.sav
	Active Dataset	DataSet5
	Filter	<none>
	Weight	<none>
	Split File	<none>
	N of Rows in Working Data File	30
Missing Value Handling	Definition of Missing	User-defined missing values are treated as missing.
	Cases Used	Statistics are based on all cases with valid data for all variables in the model.
Syntax		UNIANOVA ContextPrestim BY GENOTYPE SEX /METHOD=SSTYPE(3) /INTERCEPT=INCLUDE /EMMEANS=TABLES (GENOTYPE) /EMMEANS=TABLES (SEX) /PRINT ETASQ /CRITERIA=ALPHA(.05) /DESIGN=GENOTYPE SEX.
Resources	Processor Time	00:00:00.02
	Elapsed Time	00:00:00.00

Between-Subjects Factors

		N
GENOTYPE	KI	15
	WT	15
SEX	F	14
	M	16

Tests of Between-Subjects Effects

Dependent Variable: Context-Prestim

Source	Type III Sum of Squares	df	Mean Square	F	Sig.	Partial Eta Squared
Corrected Model	.382[a]	2	.191	12.884	<.001	.488
Intercept	4.547	1	4.547	306.515	<.001	.919
GENOTYPE	.379	1	.379	25.566	<.001	.486
SEX	.003	1	.003	.202	.657	.007
Error	.401	27	.015			
Total	5.335	30				
Corrected Total	.783	29				

a. R Squared = .488 (Adjusted R Squared = .450)

Estimated Marginal Means

1. GENOTYPE

Dependent Variable: Context-Prestim

GENOTYPE	Mean	Std. Error	95% Confidence Interval	
			Lower Bound	Upper Bound
KI	.278	.031	.213	.342
WT	.503	.031	.438	.567

2. SEX

Dependent Variable: Context-Prestim

SEX	Mean	Std. Error	95% Confidence Interval	
			Lower Bound	Upper Bound
F	.400	.033	.333	.467
M	.380	.030	.318	.443

```
UNIANOVA CuePrestim BY GENOTYPE SEX
/METHOD=SSTYPE(3)
/INTERCEPT=INCLUDE
/EMMEANS=TABLES(GENOTYPE)
/EMMEANS=TABLES(SEX)
/PRINT ETASQ
/CRITERIA=ALPHA(.05)
/DESIGN=GENOTYPE SEX.
```

Univariate Analysis of Variance

Notes

Output Created		22-AUG-2022 16:04:...
Comments		
Input	Data	/Users/jeffneul/Jeff's Research/Neul Manuscripts/FoxG1/June 2022 stat sex and effect size/SPSS files/SPSS Data Files/June 2022 CF sex EF data.sav
	Active Dataset	DataSet5
	Filter	<none>
	Weight	<none>
	Split File	<none>
	N of Rows in Working Data File	30
Missing Value Handling	Definition of Missing	User-defined missing values are treated as missing.
	Cases Used	Statistics are based on all cases with valid data for all variables in the model.
Syntax		UNIANOVA CuePrestim BY GENOTYPE SEX<br>/METHOD=SSTYPE(3)<br>/INTERCEPT=INCLUDE<br>/EMMEANS=TABLES (GENOTYPE)<br>/EMMEANS=TABLES (SEX)<br>/PRINT ETASQ<br>/CRITERIA=ALPHA(.05)<br>/DESIGN=GENOTYPE SEX.
Resources	Processor Time	00:00:00.02
	Elapsed Time	00:00:00.00

Between-Subjects Factors

		N
GENOTYPE	KI	15
	WT	15
SEX	F	14
	M	16

Tests of Between-Subjects Effects

Dependent Variable: Cue-Prestim

Source	Type III Sum of Squares	df	Mean Square	F	Sig.	Partial Eta Squared
Corrected Model	.320[a]	2	.160	13.972	<.001	.509
Intercept	7.643	1	7.643	666.962	<.001	.961
GENOTYPE	.305	1	.305	26.588	<.001	.496
SEX	.016	1	.016	1.355	.255	.048
Error	.309	27	.011			
Total	8.261	30				
Corrected Total	.630	29				

a. R Squared = .509 (Adjusted R Squared = .472)

Estimated Marginal Means

1. GENOTYPE

Dependent Variable: Cue-Prestim

GENOTYPE	Mean	Std. Error	95% Confidence Interval	
			Lower Bound	Upper Bound
KI	.405	.028	.348	.462
WT	.607	.028	.550	.663

2. SEX

Dependent Variable: Cue-Prestim

SEX	Mean	Std. Error	95% Confidence Interval	
			Lower Bound	Upper Bound
F	.529	.029	.470	.587
M	.483	.027	.428	.538

Marble Burying

```
UNIANOVA BURRIED BY GENOTYPE SEX
/METHOD=SSTYPE(3)
/INTERCEPT=INCLUDE
/CRITERIA=ALPHA(0.05)
/DESIGN=GENOTYPE SEX GENOTYPE*SEX.
```

Univariate Analysis of Variance

Notes

Output Created		22-AUG-2022 16:28:...
Comments		
Input	Data	/Users/jeffneul/Jeff's Research/Neul Manuscripts/FoxG1/June 2022 stat sex and effect size/SPSS files/SPSS Data Files/June 2022 marble sex EF data.sav
	Active Dataset	DataSet6
	Filter	<none>
	Weight	<none>
	Split File	<none>
	N of Rows in Working Data File	30
Missing Value Handling	Definition of Missing	User-defined missing values are treated as missing.
	Cases Used	Statistics are based on all cases with valid data for all variables in the model.
Syntax		UNIANOVA BURRIED BY GENOTYPE SEX /METHOD=SSTYPE(3) /INTERCEPT=INCLUDE /CRITERIA=ALPHA (0.05) /DESIGN=GENOTYPE SEX GENOTYPE*SEX.
Resources	Processor Time	00:00:00.01
	Elapsed Time	00:00:00.00

Between-Subjects Factors

		N
GENOTYPE	KI	15
	WT	15
SEX	F	14
	M	16

Tests of Between-Subjects Effects

Dependent Variable: %BURRIED

Source	Type III Sum of Squares	df	Mean Square	F	Sig.
Corrected Model	2612.649 [a]	3	870.883	1.723	.187
Intercept	71696.801	1	71696.801	141.849	<.001
GENOTYPE	2275.015	1	2275.015	4.501	.044
SEX	196.801	1	196.801	.389	.538
GENOTYPE * SEX	75.015	1	75.015	.148	.703
Error	13141.518	26	505.443		
Total	88275.000	30			
Corrected Total	15754.167	29			

a. R Squared = .166 (Adjusted R Squared = .070)

```
UNIANOVA BURRIED BY GENOTYPE SEX
/METHOD=SSTYPE(3)
/INTERCEPT=INCLUDE
/EMMEANS=TABLES(GENOTYPE)
/EMMEANS=TABLES(SEX)
/PRINT ETASQ
/CRITERIA=ALPHA(.05)
/DESIGN=GENOTYPE SEX.
```

Univariate Analysis of Variance

Notes

Output Created		22-AUG-2022 16:28:...
Comments		
Input	Data	/Users/jeffneul/Jeff's Research/Neul Manuscripts/FoxG1/June 2022 stat sex and effect size/SPSS files/SPSS Data Files/June 2022 marble sex EF data.sav
	Active Dataset	DataSet6
	Filter	<none>
	Weight	<none>
	Split File	<none>
	N of Rows in Working Data File	30
Missing Value Handling	Definition of Missing	User-defined missing values are treated as missing.
	Cases Used	Statistics are based on all cases with valid data for all variables in the model.
Syntax		UNIANOVA BURRIED BY GENOTYPE SEX /METHOD=SSTYPE(3) /INTERCEPT=INCLUDE /EMMEANS=TABLES (GENOTYPE) /EMMEANS=TABLES (SEX) /PRINT ETASQ /CRITERIA=ALPHA(.05) /DESIGN=GENOTYPE SEX.
Resources	Processor Time	00:00:00.01
	Elapsed Time	00:00:00.00

Between-Subjects Factors

		N
GENOTYPE	KI	15
	WT	15
SEX	F	14
	M	16

Tests of Between-Subjects Effects

Dependent Variable: %BURRIED

Source	Type III Sum of Squares	df	Mean Square	F	Sig.	Partial Eta Squared
Corrected Model	2537.634[a]	2	1268.817	2.592	.093	.161
Intercept	71696.801	1	71696.801	146.469	<.001	.844
GENOTYPE	2340.833	1	2340.833	4.782	.038	.150
SEX	196.801	1	196.801	.402	.531	.015
Error	13216.533	27	489.501			
Total	88275.000	30				
Corrected Total	15754.167	29				

a. R Squared = .161 (Adjusted R Squared = .099)

Estimated Marginal Means

1. GENOTYPE

Dependent Variable: %BURRIED

GENOTYPE	Mean	Std. Error	95% Confidence Interval	
			Lower Bound	Upper Bound
KI	40.162	5.719	28.428	51.896
WT	57.829	5.719	46.095	69.563

2. SEX

Dependent Variable: %BURRIED

SEX	Mean	Std. Error	95% Confidence Interval	
			Lower Bound	Upper Bound
F	46.429	5.913	34.296	58.561
M	51.563	5.531	40.213	62.912

Tube Test

```
UNIANOVA Average BY Genotype Sex
/METHOD=SSTYPE(3)
/INTERCEPT=INCLUDE
/CRITERIA=ALPHA(0.05)
/DESIGN=Genotype Sex Genotype*Sex.
```

Univariate Analysis of Variance

Notes

Output Created		22-AUG-2022 16:41:...
Comments		
Input	Data	/Users/jeffneul/Jeff's Research/Neul Manuscripts/FoxG1/June 2022 stat sex and effect size/SPSS files/SPSS Data Files/June 2022 Tubesex EF data.sav
	Active Dataset	DataSet7
	Filter	<none>
	Weight	<none>
	Split File	<none>
	N of Rows in Working Data File	30
Missing Value Handling	Definition of Missing	User-defined missing values are treated as missing.
	Cases Used	Statistics are based on all cases with valid data for all variables in the model.
Syntax		UNIANOVA Average BY Genotype Sex /METHOD=SSTYPE(3) /INTERCEPT=INCLUDE /CRITERIA=ALPHA (0.05) /DESIGN=Genotype Sex Genotype*Sex.
Resources	Processor Time	00:00:00.01
	Elapsed Time	00:00:00.00

Between-Subjects Factors

		N
Genotype	KI	15
	WT	15
Sex	F	14
	M	16

Tests of Between-Subjects Effects

Dependent Variable: Average

Source	Type III Sum of Squares	df	Mean Square	F	Sig.
Corrected Model	1.694[a]	3	.565	3.627	.026
Intercept	6.649	1	6.649	42.712	<.001
Genotype	1.064	1	1.064	6.834	.015
Sex	.337	1	.337	2.162	.153
Genotype * Sex	.337	1	.337	2.162	.153
Error	4.048	26	.156		
Total	12.750	30			
Corrected Total	5.742	29			

a. R Squared = .295 (Adjusted R Squared = .214)

```
UNIANOVA Average BY Genotype Sex
/METHOD=SSTYPE(3)
/INTERCEPT=INCLUDE
/EMMEANS=TABLES(Genotype)
/EMMEANS=TABLES(Sex)
/PRINT ETASQ
/CRITERIA=ALPHA(.05)
/DESIGN=Genotype Sex.
```

Univariate Analysis of Variance

Notes

Output Created		22-AUG-2022 16:41:...
Comments		
Input	Data	/Users/jeffneul/Jeff's Research/Neul Manuscripts/FoxG1/June 2022 stat sex and effect size/SPSS files/SPSS Data Files/June 2022 Tubesex EF data.sav
	Active Dataset	DataSet7
	Filter	<none>
	Weight	<none>
	Split File	<none>
	N of Rows in Working Data File	30
Missing Value Handling	Definition of Missing	User-defined missing values are treated as missing.
	Cases Used	Statistics are based on all cases with valid data for all variables in the model.
Syntax		UNIANOVA Average BY Genotype Sex<br>/METHOD=SSTYPE(3)<br>/INTERCEPT=INCLUDE<br>/EMMEANS=TABLES (Genotype)<br>/EMMEANS=TABLES (Sex)<br>/PRINT ETASQ<br>/CRITERIA=ALPHA(.05)<br>/DESIGN=Genotype Sex.
Resources	Processor Time	00:00:00.02
	Elapsed Time	00:00:00.00

Between-Subjects Factors

		N
Genotype	KI	15
	WT	15
Sex	F	14
	M	16

Tests of Between-Subjects Effects

Dependent Variable: Average

Source	Type III Sum of Squares	df	Mean Square	F	Sig.	Partial Eta Squared
Corrected Model	1.357[a]	2	.679	4.180	.026	.236
Intercept	7.188	1	7.188	44.268	<.001	.621
Genotype	1.154	1	1.154	7.105	.013	.208
Sex	.349	1	.349	2.150	.154	.074
Error	4.384	27	.162			
Total	12.750	30				
Corrected Total	5.742	29				

a. R Squared = .236 (Adjusted R Squared = .180)

Estimated Marginal Means

1. Genotype

Dependent Variable: Average

Genotype	Mean	Std. Error	95% Confidence Interval	
			Lower Bound	Upper Bound
KI	.688	.105	.473	.904
WT	.293	.104	.079	.506

2. Sex

Dependent Variable: Average

Sex	Mean	Std. Error	95% Confidence Interval	
			Lower Bound	Upper Bound
F	.600	.108	.378	.822
M	.382	.101	.174	.589

Nest Building

```
UNIANOVA NestScore BY Genotype Sex
/METHOD=SSTYPE(3)
/INTERCEPT=INCLUDE
/CRITERIA=ALPHA(0.05)
/DESIGN=Genotype Sex Genotype*Sex.
```

Univariate Analysis of Variance

Notes

Output Created		23-AUG-2022 14:24:...
Comments		
Input	Data	/Users/jeffneul/Jeff's Research/Neul Manuscripts/FoxG1/June_Aug 2022 stat sex and effect size/SPSS files/SPSS Data Files/June 2022 Nest sex EF data.sav
	Active Dataset	DataSet4
	Filter	<none>
	Weight	<none>
	Split File	<none>
	N of Rows in Working Data File	30
Missing Value Handling	Definition of Missing	User-defined missing values are treated as missing.
	Cases Used	Statistics are based on all cases with valid data for all variables in the model.
Syntax		UNIANOVA NestScore BY Genotype Sex /METHOD=SSTYPE(3) /INTERCEPT=INCLUDE /CRITERIA=ALPHA (0.05) /DESIGN=Genotype Sex Genotype*Sex.
Resources	Processor Time	00:00:00.01
	Elapsed Time	00:00:00.00

```
[DataSet4] /Users/jeffneul/Jeff's Research/Neul Manuscripts/FoxG1/June_Aug 202
2 stat sex and effect size/SPSS files/SPSS Data Files/June 2022 Nest sex EF da
ta.sav
```

Between-Subjects Factors

		N
Genotype	KI	15
	WT	15
Sex	F	14
	M	16

Tests of Between-Subjects Effects

Dependent Variable: Nest Score

Source	Type III Sum of Squares	df	Mean Square	F	Sig.
Corrected Model	7.094[a]	3	2.365	2.340	.097
Intercept	535.507	1	535.507	529.957	<.001
Genotype	6.254	1	6.254	6.189	.020
Sex	.007	1	.007	.007	.933
Genotype * Sex	.554	1	.554	.548	.466
Error	26.272	26	1.010		
Total	571.000	30			
Corrected Total	33.367	29			

a. R Squared = .213 (Adjusted R Squared = .122)

```
UNIANOVA shredded BY Genotype Sex
/METHOD=SSTYPE(3)
/INTERCEPT=INCLUDE
/CRITERIA=ALPHA(0.05)
/DESIGN=Genotype Sex Genotype*Sex.
```

Univariate Analysis of Variance

Notes

Output Created		23-AUG-2022 14:24:...
Comments		
Input	Data	/Users/jeffneul/Jeff's Research/Neul Manuscripts/FoxG1/June _Aug 2022 stat sex and effect size/SPSS files/SPSS Data Files/June 2022 Nest sex EF data.sav
	Active Dataset	DataSet4
	Filter	<none>
	Weight	<none>
	Split File	<none>
	N of Rows in Working Data File	30
Missing Value Handling	Definition of Missing	User-defined missing values are treated as missing.
	Cases Used	Statistics are based on all cases with valid data for all variables in the model.
Syntax		UNIANOVA shredded BY Genotype Sex /METHOD=SSTYPE(3) /INTERCEPT=INCLUDE /CRITERIA=ALPHA (0.05) /DESIGN=Genotype Sex Genotype*Sex.
Resources	Processor Time	00:00:00.01
	Elapsed Time	00:00:00.00

Between-Subjects Factors

		N
Genotype	KI	15
	WT	15
Sex	F	14
	M	16

Tests of Between-Subjects Effects

Dependent Variable: % shredded

Source	Type III Sum of Squares	df	Mean Square	F	Sig.
Corrected Model	3075.830[a]	3	1025.277	1.871	.159
Intercept	162169.074	1	162169.074	295.895	<.001
Genotype	2062.888	1	2062.888	3.764	.063
Sex	69.192	1	69.192	.126	.725
Genotype * Sex	763.098	1	763.098	1.392	.249
Error	14249.641	26	548.063		
Total	179770.198	30			
Corrected Total	17325.471	29			

a. R Squared = .178 (Adjusted R Squared = .083)

```
UNIANOVA NestScore BY Genotype Sex
/METHOD=SSTYPE(3)
/INTERCEPT=INCLUDE
/EMMEANS=TABLES(Genotype)
/EMMEANS=TABLES(Sex)
/PRINT ETASQ
/CRITERIA=ALPHA(.05)
/DESIGN=Genotype Sex.
```

Univariate Analysis of Variance

Notes

Output Created		23-AUG-2022 14:25:...
Comments		
Input	Data	/Users/jeffneul/Jeff's Research/Neul Manuscripts/FoxG1/June _Aug 2022 stat sex and effect size/SPSS files/SPSS Data Files/June 2022 Nest sex EF data.sav
	Active Dataset	DataSet4
	Filter	<none>
	Weight	<none>
	Split File	<none>
	N of Rows in Working Data File	30
Missing Value Handling	Definition of Missing	User-defined missing values are treated as missing.
	Cases Used	Statistics are based on all cases with valid data for all variables in the model.
Syntax		UNIANOVA NestScore BY Genotype Sex /METHOD=SSTYPE(3) /INTERCEPT=INCLUDE /EMMEANS=TABLES (Genotype) /EMMEANS=TABLES (Sex) /PRINT ETASQ /CRITERIA=ALPHA(.05) /DESIGN=Genotype Sex.
Resources	Processor Time	00:00:00.01
	Elapsed Time	00:00:00.00

Between-Subjects Factors

		N
Genotype	KI	15
	WT	15
Sex	F	14
	M	16

Tests of Between-Subjects Effects

Dependent Variable: Nest Score

Source	Type III Sum of Squares	df	Mean Square	F	Sig.	Partial Eta Squared
Corrected Model	6.541[a]	2	3.270	3.292	.053	.196
Intercept	535.507	1	535.507	538.980	<.001	.952
Genotype	6.533	1	6.533	6.576	.016	.196
Sex	.007	1	.007	.007	.932	.000
Error	26.826	27	.994			
Total	571.000	30				
Corrected Total	33.367	29				

a. R Squared = .196 (Adjusted R Squared = .136)

Estimated Marginal Means

1. Genotype

Dependent Variable: Nest Score

Genotype	Mean	Std. Error	95% Confidence Interval	
			Lower Bound	Upper Bound
KI	3.768	.258	3.239	4.296
WT	4.701	.258	4.172	5.230

2. Sex

Dependent Variable: Nest Score

Sex	Mean	Std. Error	95% Confidence Interval	
			Lower Bound	Upper Bound
F	4.250	.266	3.703	4.797
M	4.219	.249	3.707	4.730

```
UNIANOVA shredded BY Genotype Sex
/METHOD=SSTYPE(3)
/INTERCEPT=INCLUDE
/EMMEANS=TABLES(Genotype)
/EMMEANS=TABLES(Sex)
/PRINT ETASQ
/CRITERIA=ALPHA(.05)
/DESIGN=Genotype Sex.
```

Univariate Analysis of Variance

Notes

Output Created		23-AUG-2022 14:25:...
Comments		
Input	Data	/Users/jeffneul/Jeff's Research/Neul Manuscripts/FoxG1/June_Aug 2022 stat sex and effect size/SPSS files/SPSS Data Files/June 2022 Nest sex EF data.sav
	Active Dataset	DataSet4
	Filter	<none>
	Weight	<none>
	Split File	<none>
	N of Rows in Working Data File	30
Missing Value Handling	Definition of Missing	User-defined missing values are treated as missing.
	Cases Used	Statistics are based on all cases with valid data for all variables in the model.
Syntax		UNIANOVA shredded BY Genotype Sex /METHOD=SSTYPE(3) /INTERCEPT=INCLUDE /EMMEANS=TABLES (Genotype) /EMMEANS=TABLES (Sex) /PRINT ETASQ /CRITERIA=ALPHA(.05) /DESIGN=Genotype Sex.
Resources	Processor Time	00:00:00.02
	Elapsed Time	00:00:00.00

Between-Subjects Factors

		N
Genotype	KI	15
	WT	15
Sex	F	14
	M	16

Tests of Between-Subjects Effects

Dependent Variable: % shredded

Source	Type III Sum of Squares	df	Mean Square	F	Sig.	Partial Eta Squared
Corrected Model	2312.731[a]	2	1156.366	2.080	.145	.133
Intercept	162169.074	1	162169.074	291.657	<.001	.915
Genotype	2243.540	1	2243.540	4.035	.055	.130
Sex	69.192	1	69.192	.124	.727	.005
Error	15012.740	27	556.027			
Total	179770.198	30				
Corrected Total	17325.471	29				

a. R Squared = .133 (Adjusted R Squared = .069)

Estimated Marginal Means

1. Genotype

Dependent Variable: % shredded

Genotype	Mean	Std. Error	95% Confidence Interval	
			Lower Bound	Upper Bound
KI	65.039	6.095	52.533	77.545
WT	82.335	6.095	69.828	94.841

2. Sex

Dependent Variable: % shredded

Sex	Mean	Std. Error	95% Confidence Interval	
			Lower Bound	Upper Bound
F	75.209	6.302	62.278	88.140
M	72.165	5.895	60.069	84.261

3 Chamber

```
UNIANOVA SocialIndexCup BY Geno SEX
/METHOD=SSTYPE(3)
/INTERCEPT=INCLUDE
/CRITERIA=ALPHA(0.05)
/DESIGN=Geno SEX Geno*SEX.
```

Univariate Analysis of Variance

Notes

Output Created		22-AUG-2022 16:59:...
Comments		
Input	Data	/Users/jeffneul/Jeff's Research/Neul Manuscripts/FoxG1/June 2022 stat sex and effect size/SPSS files/SPSS Data Files/June 2022 3CH Social sex EF data.sav
	Active Dataset	DataSet9
	Filter	<none>
	Weight	<none>
	Split File	<none>
	N of Rows in Working Data File	27
Missing Value Handling	Definition of Missing	User-defined missing values are treated as missing.
	Cases Used	Statistics are based on all cases with valid data for all variables in the model.
Syntax		UNIANOVA SocialIndexCup BY Geno SEX /METHOD=SSTYPE(3) /INTERCEPT=INCLUDE /CRITERIA=ALPHA(0.05) /DESIGN=Geno SEX Geno*SEX.
Resources	Processor Time	00:00:00.01
	Elapsed Time	00:00:00.00

Between-Subjects Factors

		N
Geno	KI	16
	WT	11
SEX	F	14
	M	13

Tests of Between-Subjects Effects

Dependent Variable: Social Index Cup

Source	Type III Sum of Squares	df	Mean Square	F	Sig.
Corrected Model	.205[a]	3	.068	1.395	.269
Intercept	.727	1	.727	14.818	<.001
Geno	.191	1	.191	3.903	.060
SEX	.040	1	.040	.808	.378
Geno * SEX	.001	1	.001	.013	.910
Error	1.128	23	.049		
Total	2.310	27			
Corrected Total	1.334	26			

a. R Squared = .154 (Adjusted R Squared = .044)

```
UNIANOVA SocialIndexChamber BY Geno SEX
/METHOD=SSTYPE(3)
/INTERCEPT=INCLUDE
/CRITERIA=ALPHA(0.05)
/DESIGN=Geno SEX Geno*SEX.
```

Univariate Analysis of Variance

Notes

Output Created		22-AUG-2022 16:59:...
Comments		
Input	Data	/Users/jeffneul/Jeff's Research/Neul Manuscripts/FoxG1/June 2022 stat sex and effect size/SPSS files/SPSS Data Files/June 2022 3CH Social sex EF data.sav
	Active Dataset	DataSet9
	Filter	<none>
	Weight	<none>
	Split File	<none>
	N of Rows in Working Data File	27
Missing Value Handling	Definition of Missing	User-defined missing values are treated as missing.
	Cases Used	Statistics are based on all cases with valid data for all variables in the model.
Syntax		UNIANOVA SocialIndexChamber BY Geno SEX /METHOD=SSTYPE(3) /INTERCEPT=INCLUDE /CRITERIA=ALPHA (0.05) /DESIGN=Geno SEX Geno*SEX.
Resources	Processor Time	00:00:00.01
	Elapsed Time	00:00:00.00

Between-Subjects Factors

		N
Geno	KI	16
	WT	11
SEX	F	14
	M	13

Tests of Between-Subjects Effects

Dependent Variable: Social Index Chamber

Source	Type III Sum of Squares	df	Mean Square	F	Sig.
Corrected Model	.137[a]	3	.046	1.550	.228
Intercept	.474	1	.474	16.022	<.001
Geno	.137	1	.137	4.637	.042
SEX	.007	1	.007	.238	.630
Geno * SEX	.004	1	.004	.135	.716
Error	.680	23	.030		
Total	1.467	27			
Corrected Total	.817	26			

a. R Squared = .168 (Adjusted R Squared = .060)

```
UNIANOVA SocialIndexCup BY Geno SEX
/METHOD=SSTYPE(3)
/INTERCEPT=INCLUDE
/EMMEANS=TABLES(Geno)
/EMMEANS=TABLES(SEX)
/PRINT ETASQ
/CRITERIA=ALPHA(.05)
/DESIGN=Geno SEX.
```

Univariate Analysis of Variance

Notes

Output Created		22-AUG-2022 16:59:...
Comments		
Input	Data	/Users/jeffneul/Jeff's Research/Neul Manuscripts/FoxG1/June 2022 stat sex and effect size/SPSS files/SPSS Data Files/June 2022 3CH Social sex EF data.sav
	Active Dataset	DataSet9
	Filter	<none>
	Weight	<none>
	Split File	<none>
	N of Rows in Working Data File	27
Missing Value Handling	Definition of Missing	User-defined missing values are treated as missing.
	Cases Used	Statistics are based on all cases with valid data for all variables in the model.
Syntax		UNIANOVA SocialIndexCup BY Geno SEX<br>/METHOD=SSTYPE(3)<br>/INTERCEPT=INCLUDE<br>/EMMEANS=TABLES(Geno)<br>/EMMEANS=TABLES(SEX)<br>/PRINT ETASQ<br>/CRITERIA=ALPHA(.05)<br>/DESIGN=Geno SEX.
Resources	Processor Time	00:00:00.02
	Elapsed Time	00:00:00.00

Between-Subjects Factors

		N
Geno	KI	16
	WT	11
SEX	F	14
	M	13

Tests of Between-Subjects Effects

Dependent Variable: Social Index Cup

Source	Type III Sum of Squares	df	Mean Square	F	Sig.	Partial Eta Squared
Corrected Model	.205[a]	2	.102	2.176	.135	.154
Intercept	.772	1	.772	16.407	<.001	.406
Geno	.191	1	.191	4.071	.055	.145
SEX	.039	1	.039	.836	.370	.034
Error	1.129	24	.047			
Total	2.310	27				
Corrected Total	1.334	26				

a. R Squared = .154 (Adjusted R Squared = .083)

Estimated Marginal Means

1. Geno

Dependent Variable: Social Index Cup

Geno	Mean	Std. Error	95% Confidence Interval Lower Bound	95% Confidence Interval Upper Bound
KI	.260	.054	.147	.372
WT	.085	.066	-.052	.222

2. SEX

Dependent Variable: Social Index Cup

SEX	Mean	Std. Error	95% Confidence Interval Lower Bound	95% Confidence Interval Upper Bound
F	.211	.058	.092	.331
M	.134	.062	.005	.262

```
UNIANOVA SocialIndexChamberBY Geno SEX
/METHOD=SSTYPE(3)
/INTERCEPT=INCLUDE
/EMMEANS=TABLES(Geno)
/EMMEANS=TABLES(SEX)
/PRINT ETASQ
/CRITERIA=ALPHA(.05)
/DESIGN=Geno SEX.
```

Univariate Analysis of Variance

Notes

Output Created		22-AUG-2022 17:00:...
Comments		
Input	Data	/Users/jeffneul/Jeff's Research/Neul Manuscripts/FoxG1/June 2022 stat sex and effect size/SPSS files/SPSS Data Files/June 2022 3CH Social sex EF data.sav
	Active Dataset	DataSet9
	Filter	<none>
	Weight	<none>
	Split File	<none>
	N of Rows in Working Data File	27
Missing Value Handling	Definition of Missing	User-defined missing values are treated as missing.
	Cases Used	Statistics are based on all cases with valid data for all variables in the model.
Syntax		UNIANOVA SocialIndexChamber BY Geno SEX /METHOD=SSTYPE(3) /INTERCEPT=INCLUDE /EMMEANS=TABLES(Geno) /EMMEANS=TABLES(SEX) /PRINT ETASQ /CRITERIA=ALPHA(.05) /DESIGN=Geno SEX.
Resources	Processor Time	00:00:00.02
	Elapsed Time	00:00:00.00

Between-Subjects Factors

		N
Geno	KI	16
	WT	11
SEX	F	14
	M	13

Tests of Between-Subjects Effects

Dependent Variable: Social Index Chamber

Source	Type III Sum of Squares	df	Mean Square	F	Sig.	Partial Eta Squared
Corrected Model	.133[a]	2	.067	2.342	.118	.163
Intercept	.516	1	.516	18.119	<.001	.430
Geno	.133	1	.133	4.684	.041	.163
SEX	.005	1	.005	.182	.674	.008
Error	.684	24	.028			
Total	1.467	27				
Corrected Total	.817	26				

a. R Squared = .163 (Adjusted R Squared = .094)

Estimated Marginal Means

1. Geno

Dependent Variable: Social Index Chamber

Geno	Mean	Std. Error	95% Confidence Interval	
			Lower Bound	Upper Bound
KI	.214	.042	.127	.302
WT	.068	.052	-.039	.175

2. SEX

Dependent Variable: Social Index Chamber

SEX	Mean	Std. Error	95% Confidence Interval	
			Lower Bound	Upper Bound
F	.155	.045	.062	.248
M	.127	.049	.027	.227

```
UNIANOVA NoveltyIndexCup BY Geno SEX
/METHOD=SSTYPE(3)
/INTERCEPT=INCLUDE
/CRITERIA=ALPHA(0.05)
/DESIGN=Geno SEX Geno*SEX.
```

Univariate Analysis of Variance

Notes

Output Created		22-AUG-2022 17:11:...
Comments		
Input	Data	/Users/jeffneul/Jeff's Research/Neul Manuscripts/FoxG1/June 2022 stat sex and effect size/SPSS files/SPSS Data Files/June 2022 3CH Novelty sex EF data.sav
	Active Dataset	DataSet10
	Filter	<none>
	Weight	<none>
	Split File	<none>
	N of Rows in Working Data File	27
Missing Value Handling	Definition of Missing	User-defined missing values are treated as missing.
	Cases Used	Statistics are based on all cases with valid data for all variables in the model.
Syntax		UNIANOVA NoveltyIndexCup BY Geno SEX /METHOD=SSTYPE(3) /INTERCEPT=INCLUDE /CRITERIA=ALPHA (0.05) /DESIGN=Geno SEX Geno*SEX.
Resources	Processor Time	00:00:00.01
	Elapsed Time	00:00:00.00

Between-Subjects Factors

		N
Geno	KI	16
	WT	11
SEX	F	14
	M	13

Tests of Between-Subjects Effects

Dependent Variable: Novelty Index Cup

Source	Type III Sum of Squares	df	Mean Square	F	Sig.
Corrected Model	.394[a]	3	.131	1.485	.245
Intercept	1.275	1	1.275	14.414	<.001
Geno	.318	1	.318	3.597	.071
SEX	.001	1	.001	.013	.910
Geno * SEX	.091	1	.091	1.032	.320
Error	2.035	23	.088		
Total	3.450	27			
Corrected Total	2.429	26			

a. R Squared = .162 (Adjusted R Squared = .053)

```
UNIANOVA NoveltyIndexChamberBY Geno SEX
/METHOD=SSTYPE(3)
/INTERCEPT=INCLUDE
/CRITERIA=ALPHA(0.05)
/DESIGN=Geno SEX Geno*SEX.
```

Univariate Analysis of Variance

Notes

Output Created		22-AUG-2022 17:11:...
Comments		
Input	Data	/Users/jeffneul/Jeff's Research/Neul Manuscripts/FoxG1/June 2022 stat sex and effect size/SPSS files/SPSS Data Files/June 2022 3CH Novelty sex EF data.sav
	Active Dataset	DataSet10
	Filter	<none>
	Weight	<none>
	Split File	<none>
	N of Rows in Working Data File	27
Missing Value Handling	Definition of Missing	User-defined missing values are treated as missing.
	Cases Used	Statistics are based on all cases with valid data for all variables in the model.
Syntax		UNIANOVA NoveltyIndexChamber BY Geno SEX /METHOD=SSTYPE(3) /INTERCEPT=INCLUDE /CRITERIA=ALPHA (0.05) /DESIGN=Geno SEX Geno*SEX.
Resources	Processor Time	00:00:00.01
	Elapsed Time	00:00:00.00

Between-Subjects Factors

		N
Geno	KI	16
	WT	11
SEX	F	14
	M	13

Tests of Between-Subjects Effects

Dependent Variable: Novelty Index Chamber

Source	Type III Sum of Squares	df	Mean Square	F	Sig.
Corrected Model	.332[a]	3	.111	2.590	.077
Intercept	1.428	1	1.428	33.428	<.001
Geno	.224	1	.224	5.234	.032
SEX	.028	1	.028	.654	.427
Geno * SEX	.032	1	.032	.739	.399
Error	.982	23	.043		
Total	2.589	27			
Corrected Total	1.314	26			

a. R Squared = .253 (Adjusted R Squared = .155)

```
UNIANOVA NoveltyIndexCup BY Geno SEX
/METHOD=SSTYPE(3)
/INTERCEPT=INCLUDE
/EMMEANS=TABLES(Geno)
/EMMEANS=TABLES(SEX)
/PRINT ETASQ
/CRITERIA=ALPHA(.05)
/DESIGN=Geno SEX.
```

Univariate Analysis of Variance

Notes

Output Created		22-AUG-2022 17:12:...
Comments		
Input	Data	/Users/jeffneul/Jeff's Research/Neul Manuscripts/FoxG1/June 2022 stat sex and effect size/SPSS files/SPSS Data Files/June 2022 3CH Novelty sex EF data.sav
	Active Dataset	DataSet10
	Filter	<none>
	Weight	<none>
	Split File	<none>
	N of Rows in Working Data File	27
Missing Value Handling	Definition of Missing	User-defined missing values are treated as missing.
	Cases Used	Statistics are based on all cases with valid data for all variables in the model.
Syntax		UNIANOVA NoveltyIndexCup BY Geno SEX<br>/METHOD=SSTYPE(3)<br>/INTERCEPT=INCLUDE<br>/EMMEANS=TABLES (Geno)<br>/EMMEANS=TABLES (SEX)<br>/PRINT ETASQ<br>/CRITERIA=ALPHA(.05)<br>/DESIGN=Geno SEX.
Resources	Processor Time	00:00:00.01
	Elapsed Time	00:00:00.00

Between-Subjects Factors

		N
Geno	KI	16
	WT	11
SEX	F	14
	M	13

Tests of Between-Subjects Effects

Dependent Variable: Novelty Index Cup

Source	Type III Sum of Squares	df	Mean Square	F	Sig.	Partial Eta Squared
Corrected Model	.303[a]	2	.151	1.709	.202	.125
Intercept	1.187	1	1.187	13.406	.001	.358
Geno	.284	1	.284	3.201	.086	.118
SEX	.001	1	.001	.011	.916	.000
Error	2.126	24	.089			
Total	3.450	27				
Corrected Total	2.429	26				

a. R Squared = .125 (Adjusted R Squared = .052)

Estimated Marginal Means

1. Geno

Dependent Variable: Novelty Index Cup

Geno	Mean	Std. Error	95% Confidence Interval	
			Lower Bound	Upper Bound
KI	.108	.075	-.047	.262
WT	.320	.091	.132	.508

2. SEX

Dependent Variable: Novelty Index Cup

SEX	Mean	Std. Error	95% Confidence Interval	
			Lower Bound	Upper Bound
F	.220	.080	.056	.384
M	.208	.086	.031	.385

```
UNIANOVA NoveltyIndexChamberBY Geno SEX
/METHOD=SSTYPE(3)
/INTERCEPT=INCLUDE
/EMMEANS=TABLES(Geno)
/EMMEANS=TABLES(SEX)
/PRINT ETASQ
/CRITERIA=ALPHA(.05)
/DESIGN=Geno SEX.
```

Univariate Analysis of Variance

Notes

Output Created		22-AUG-2022 17:12:...
Comments		
Input	Data	/Users/jeffneul/Jeff's Research/Neul Manuscripts/FoxG1/June 2022 stat sex and effect size/SPSS files/SPSS Data Files/June 2022 3CH Novelty sex EF data.sav
	Active Dataset	DataSet10
	Filter	<none>
	Weight	<none>
	Split File	<none>
	N of Rows in Working Data File	27
Missing Value Handling	Definition of Missing	User-defined missing values are treated as missing.
	Cases Used	Statistics are based on all cases with valid data for all variables in the model.
Syntax		UNIANOVA NoveltyIndexChamber BY Geno SEX<br>/METHOD=SSTYPE(3)<br>/INTERCEPT=INCLUDE<br>/EMMEANS=TABLES (Geno)<br>/EMMEANS=TABLES (SEX)<br>/PRINT ETASQ<br>/CRITERIA=ALPHA(.05)<br>/DESIGN=Geno SEX.
Resources	Processor Time	00:00:00.02
	Elapsed Time	00:00:00.00

Between-Subjects Factors

		N
Geno	KI	16
	WT	11
SEX	F	14
	M	13

Tests of Between-Subjects Effects

Dependent Variable: Novelty Index Chamber

Source	Type III Sum of Squares	df	Mean Square	F	Sig.	Partial Eta Squared
Corrected Model	.300[a]	2	.150	3.554	.044	.229
Intercept	1.403	1	1.403	33.202	<.001	.580
Geno	.207	1	.207	4.905	.037	.170
SEX	.044	1	.044	1.046	.317	.042
Error	1.014	24	.042			
Total	2.589	27				
Corrected Total	1.314	26				

a. R Squared = .229 (Adjusted R Squared = .164)

Estimated Marginal Means

1. Geno

Dependent Variable: Novelty Index Chamber

Geno	Mean	Std. Error	95% Confidence Interval	
			Lower Bound	Upper Bound
KI	.142	.052	.035	.248
WT	.323	.063	.194	.453

2. SEX

Dependent Variable: Novelty Index Chamber

SEX	Mean	Std. Error	95% Confidence Interval	
			Lower Bound	Upper Bound
F	.274	.055	.160	.387
M	.191	.059	.069	.313
